# Physical Activity, Sedentary Leisure Time, Circulating Metabolic Markers, and Risk of Major Vascular Diseases

**DOI:** 10.1161/CIRCGEN.118.002527

**Published:** 2019-09-17

**Authors:** Yuanjie Pang, Christiana Kartsonaki, Huaidong Du, Iona Y. Millwood, Yu Guo, Yiping Chen, Zheng Bian, Ling Yang, Robin Walters, Fiona Bragg, Jun Lv, Canqing Yu, Junshi Chen, Richard Peto, Robert Clarke, Rory Collins, Derrick A. Bennett, Liming Li, Michael V. Holmes, Zhengming Chen

**Affiliations:** 1 Department of Epidemiology and Biostatistics, School of Public Health, Peking University Health Science Center, Beijing, China (Y.P., J.L., C.Y., L.L.).; 2 Clinical Trial Service Unit and Epidemiological Studies Unit (Y.P., C.K., H.D., I.Y.M., Y.C., L.Y., R.W., F.B., R.P., R. Clarke, R. Collins, D.A.B., L.L., M.V.H., Z.C.), Nuffield Department of Population Health, University of Oxford, United Kingdom.; 3 Medical Research Council Population Health Research Unit (C.K., H.D., I.Y.M., Y.C., L.Y., R.W., M.V.H., Z.C.), Nuffield Department of Population Health, University of Oxford, United Kingdom.; 4 Chinese Academy of Medical Sciences, Beijing, China (Y.G., Z.B., L.L.).; 5 National Center for Food Safety Risk Assessment, Beijing, China (J.C.).; 6 National Institute for Health Research Oxford Biomedical Research Centre, Oxford University Hospital, United Kingdom (M.V.H.).

**Keywords:** Asian Continental Ancestry Group, cardiovascular diseases, China, exercise, metabolomics

## Abstract

Physical inactivity and sedentary behavior are associated with higher risk of cardiovascular disease (CVD). Little is known about the relevance of circulating metabolites for these associations.

**Methods:**

A nested case-control study within the prospective China Kadoorie Biobank included 3195 incident CVD cases (2057 occlusive CVD and 1138 intracerebral hemorrhage) and 1465 controls aged 30 to 79 years without prior CVD or statin use at baseline. Nuclear magnetic resonance spectroscopy was used to measure 225 metabolic markers and derived traits in baseline plasma samples. Linear regression was used to relate self-reported physical activity and sedentary leisure time to biomarkers, adjusting for potential confounders. These were contrasted with associations of biomarkers with occlusive CVD risk.

**Results:**

Physical activity and sedentary leisure time were associated with >100 metabolic markers, with patterns of associations generally mirroring each other. Physical activity was inversely associated with very low and low-density and positively with large and very large HDL (high-density lipoprotein) particle concentrations. Physical activity was also inversely associated with alanine, glucose, lactate, acetoacetate, and the inflammatory marker glycoprotein acetyls. In general, associations of physical activity and sedentary leisure time with specific metabolic markers were directionally consistent with the associations of these metabolic markers with occlusive CVD risk. Overall, metabolic markers potentially explained ≈70% of the protective associations of physical activity and ≈50% of the positive associations of sedentary leisure time with occlusive CVD.

**Conclusions:**

Among Chinese adults, physical activity and sedentary behavior have opposing associations with a diverse range of circulating metabolites, which may partially explain their associations with CVD risk.

## Abstract 2

Supplemental Digital Content is available in the text.

Low physical activity and prolonged sedentary time, usually measured by self-report, have been independently associated with higher risk of cardiovascular disease (CVD) in diverse populations, including Chinese populations.^1–4^ Several biological mechanisms have been proposed that may partially explain these associations, including higher levels of adiposity, blood pressure, and systemic inflammation, as well as unfavorable changes in glucose homeostasis, insulin sensitivity, and lipid and lipoprotein profiles.^5^ Observational studies conducted in Western populations have also demonstrated that individuals with higher levels of self-reported physical activity have lower levels of LDL (low-density lipoprotein) cholesterol (LDL-C), blood glucose and inflammation (eg, fibrinogen and CRP [C-reactive protein]), and higher levels of HDL (high-density lipoprotein) cholesterol (HDL-C), with sedentary behaviors generally showing opposite associations.^5–7^

In addition to conventional biomarkers, there is also emerging evidence that physical activity may be associated with a range of circulating metabolites, including lipids and lipoproteins, amino acids, glycoprotein acetyls, glucose, and fatty acids.^8^ These metabolic biomarkers have been shown in a few prospective cohort studies to be associated with risk of occlusive vascular diseases.^9,10^ However, the existing evidence on these associations is still limited, and no study has yet simultaneously assessed the associations of physical activity and sedentary behaviors with circulating lipids and metabolic markers and of metabolic markers with CVD risks in the same population. If such associations could be reliably established in different populations, it might shed light on potential mechanisms by which physical activity and sedentary behaviors influence CVD risk. The new Physical Activity Guidelines for Americans provide guidance on the types and amounts of physical activity and but no quantitative guideline for sitting time, and, therefore, more evidence is needed to assess the cardiometabolic benefits of increasing physical activity and decreasing sitting time.^11^ Furthermore, reliable assessment of such associations in China is required because the levels and patterns of physical activity differ importantly from those in Western populations, with, for example, leisure-time physical activity only accounting for ≈5% of total physical activity in many parts of China,^12^ as opposed to 40% in typical Western populations.^13^ Moreover, the mean levels of many blood biomarkers in Chinese (eg, LDL-C) also differ markedly from Europeans.^14^

The aim of the present study was to examine the associations of self-reported physical activity and sedentary leisure time (as a measure of sedentary behaviors), with plasma lipoproteins, lipids, and other metabolic biomarkers in a nested case-control study in the China Kadoorie Biobank (CKB) and to explore how these associations with metabolic markers might explain associations of physical activity and sedentary leisure with vascular disease.

## Methods

The Methods are available in the Data Supplement. The CKB data are available at http://www.ckbiobank.org/site/Data+Access. The CKB study was approved by the Ethical Review Committee of the Chinese Center for Disease Control and Prevention and the Oxford Tropical Research Ethics Committee, University of Oxford. All participants eligible for this study had completed a written informed consent form. All methods were performed in accordance with relevant guidelines and regulations.

## Results

Among the 4660 participants included, the mean age (SD) was 46 (8) years, 50% were women, the mean level of physical activity was 23 metabolic equivalent of task hours/day, and mean sedentary leisure time was 3.1 hours/day (Table). The overall mean (SD) LDL-C concentration in controls was 84.9 (27.0) mg/dL. Compared with controls, individuals who subsequently developed myocardial infarction and ischemic stroke had lower levels of physical activity, and higher levels of adiposity, systolic blood pressure, and RPG at baseline, and were more likely to have a history of hypertension or diabetes mellitus, whereas individuals who developed intracerebral hemorrhage were more likely to have higher systolic blood pressure and a history of hypertension (Table). Individuals with CVD were less likely to consume fresh fruit, fish/seafood, and fresh eggs and more likely to consume red meat and wheat products (Table; Table I in the Data Supplement). Characteristics of individuals according to whether they developed CVD (combining myocardial infarction, ischemic stroke, and intracerebral hemorrhage cases) are provided in Table II in the Data Supplement.

**Table.**
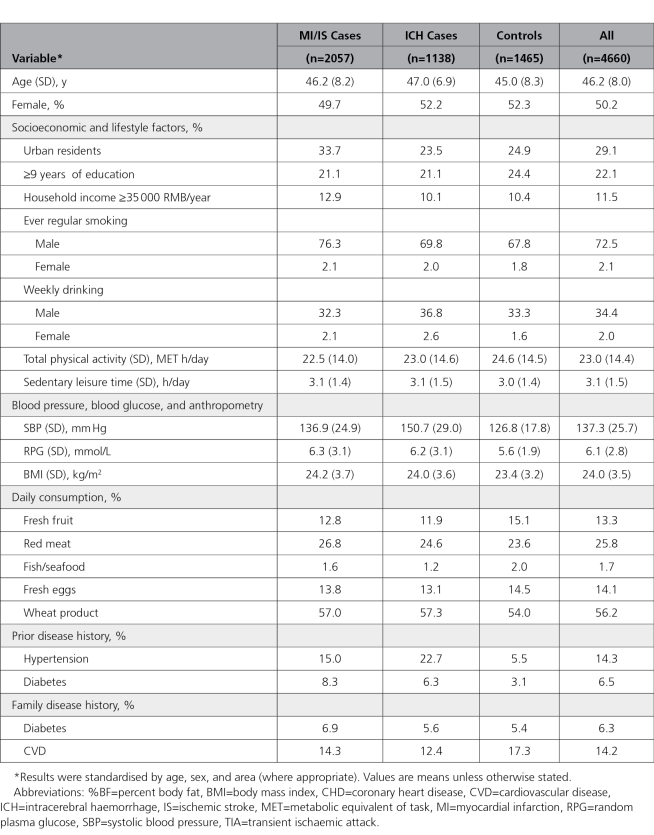
Baseline Characteristics of Participants in the Nested Case-Control Study Among Participants Without Prior CVD

**Figure 1.**
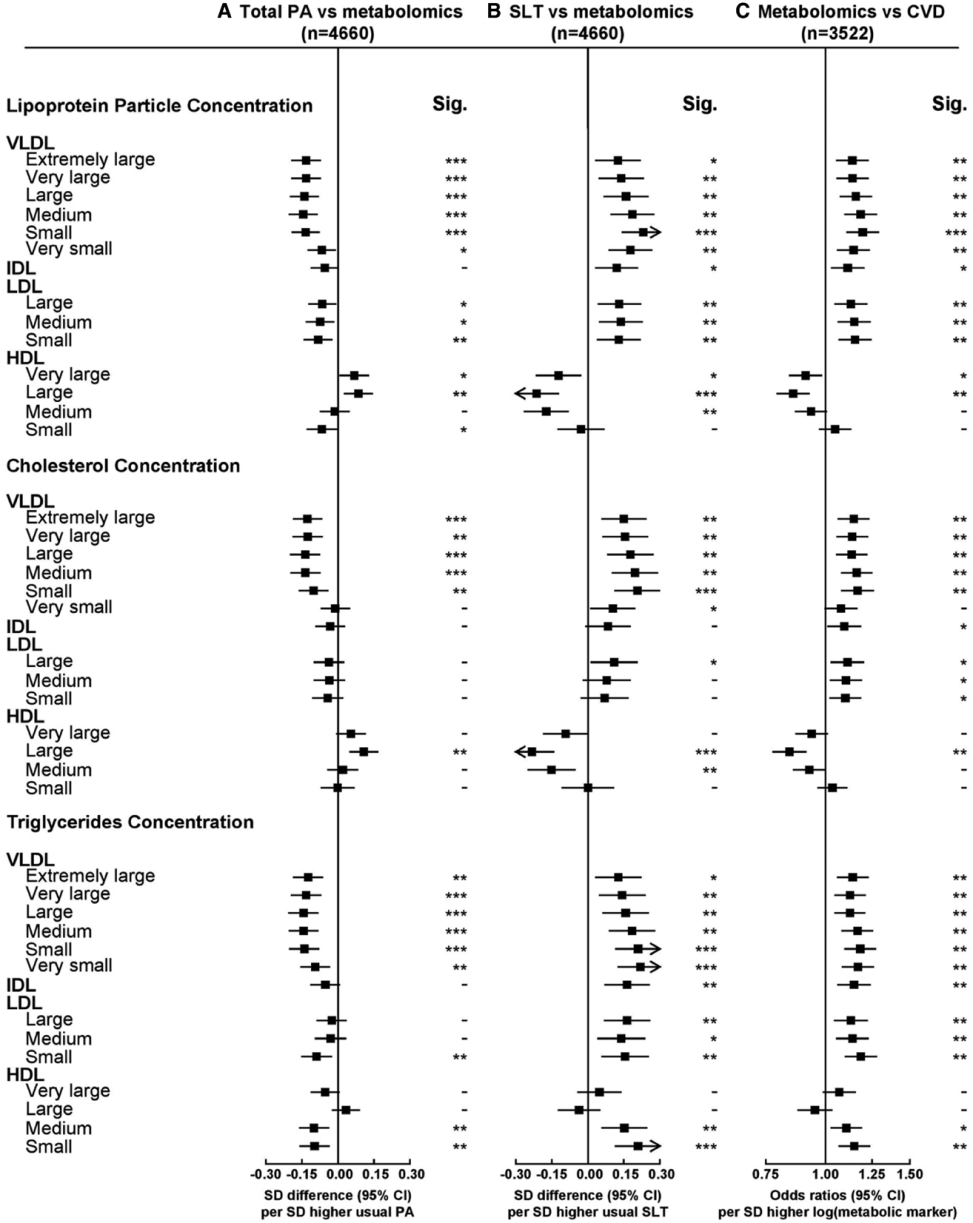
**Associations of usual total physical activity (PA) and sedentary leisure time (SLT) with lipoprotein particle concentration, cholesterol, and triglycerides, and of these metabolic markers with risks of occlusive cardiovascular disease (CVD).** Column A shows adjusted SD differences (95% CI) of log-transformed metabolic markers per 1-SD higher usual total PA, and column B shows corresponding estimates per 1-SD higher usual SLT. Column C shows adjusted odds ratios (95% CI) of CVD risk (MI and IS) per 1-SD higher log-transformed metabolic markers. Models were adjusted for age, sex, fasting time, region, smoking status, education, income, self-rated health, intake of fruit and meat, SLT (for total PA), and total PA (for SLT). The SD was 14 MET h/d for PA and 1.5 h/d for SLT. The regression dilution ratio was 0.52 for PA and 0.34 for SLT. Significance (Sig.): *P<0.05, **P<0.01, ***P<0.001 (false discovery rate [FDR]–adjusted *P* using the Benjamini-Hochberg method). HDL indicates high-density lipoprotein; IDL, intermediate-density lipoprotein; LDL, low-density lipoprotein; and VLDL, very-low-density lipoprotein.

**Figure 2.**
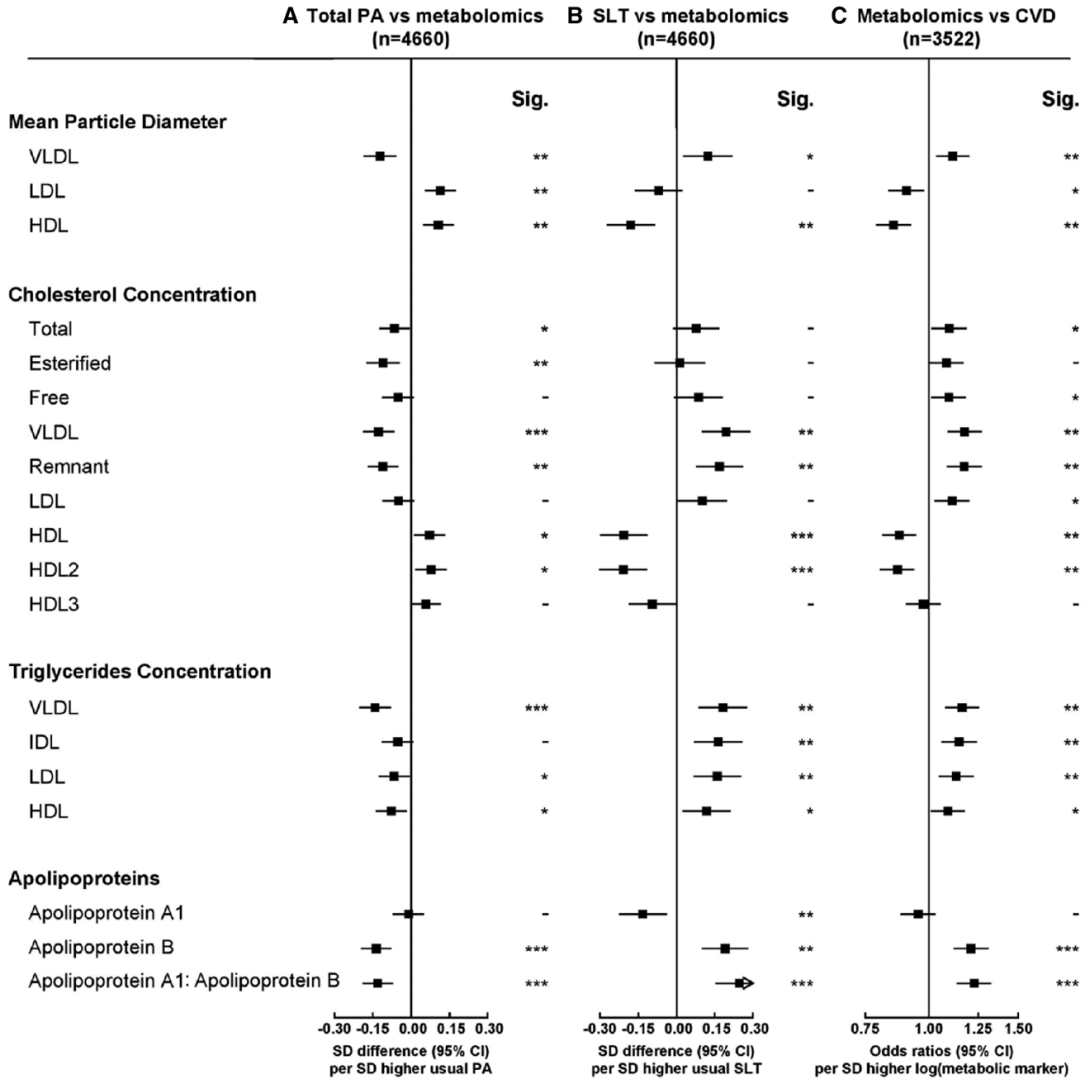
**Associations of usual total physical activity (PA) and sedentary leisure time (SLT) with mean particle diameter, cholesterol, and triglycerides and of these metabolic markers with risks of occlusive cardiovascular disease (CVD).** Conventions as in Figure 1. HDL indicates high-density lipoprotein; IDL, intermediate-density lipoprotein; LDL, low-density lipoprotein; Sig., significance; and VLDL, very-low-density lipoprotein.

Overall higher total physical activity and sedentary leisure time were associated with >100 metabolic markers at false discovery rate ≤5%, with patterns of associations generally mirroring each other (Figures 1 through 3). The associations for all 225 traits are shown in Table III in the Data Supplement. For lipoprotein particle concentrations, physical activity was inversely associated with concentrations of VLDL (very-low-density lipoprotein) and more weakly with LDL subclasses (Figure 1). For HDL particles, physical activity was positively associated with very large and large HDL and inversely with small HDL (Figure 1). Cholesterol within specific lipoprotein particles showed similar associations as the lipoprotein particles (Figure 1). There was an inverse association with remnant cholesterol but positive associations with cholesterol in HDL particles (Figure 2). Total physical activity was associated with lower triglyceride concentrations in the majority of lipoproteins (Figure 1). Higher physical activity was also associated with smaller VLDL diameter and larger LDL and HDL diameter (Figure 2).

**Figure 3.**
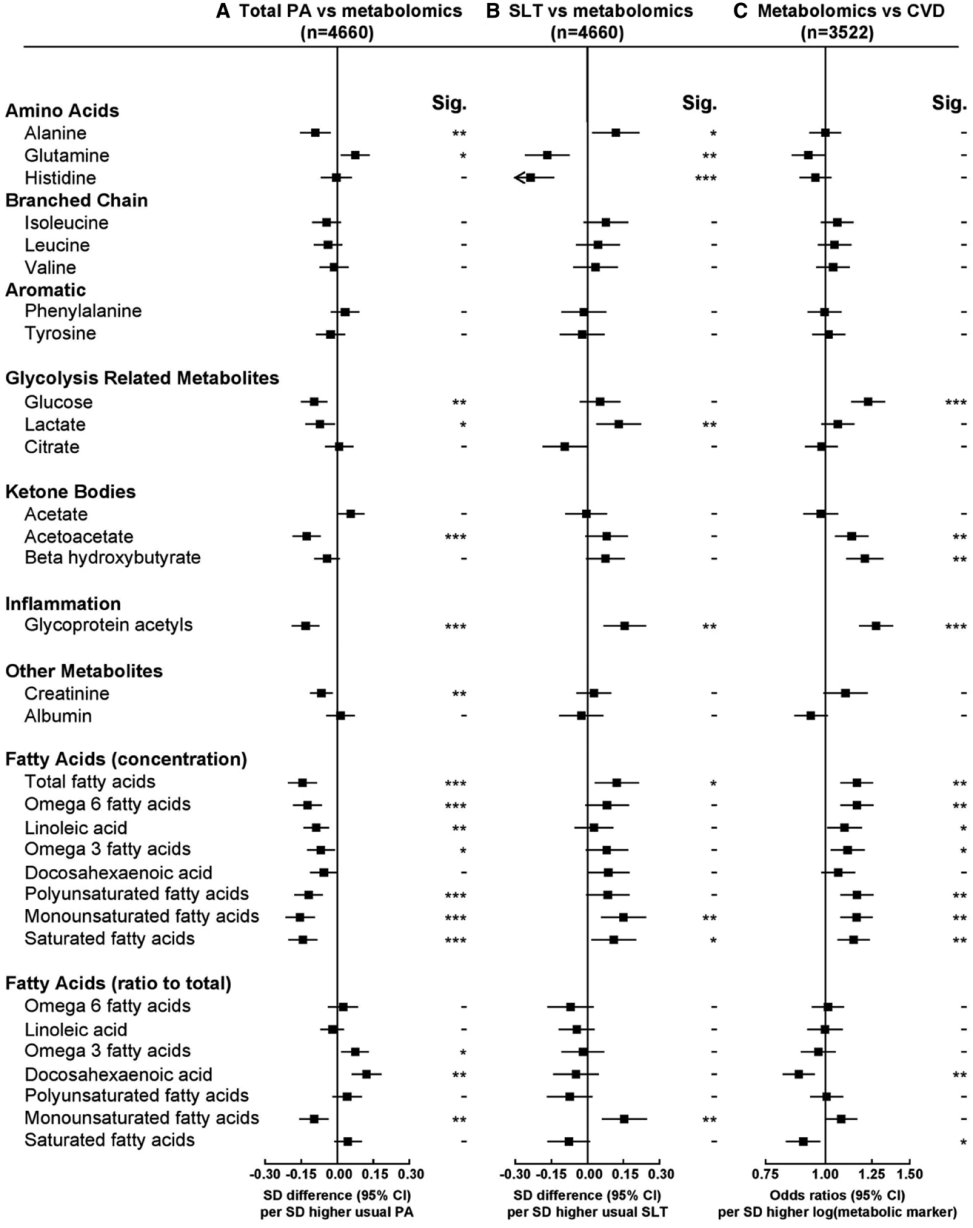
**Associations of usual total physical activity (PA) and sedentary leisure time (SLT) with other metabolic traits and of these metabolic markers with risks of occlusive cardiovascular disease (CVD).** Conventions as in Figure 1. Sig. indicates significance.

There were strong inverse associations of total physical activity with apolipoprotein B and with the ratio of apolipoprotein B to apolipoprotein A1 (Figure 2). For amino acids, physical activity was inversely associated with alanine and positively with glutamine (Figure 3). Physical activity was inversely associated with glucose, lactate, and with acetoacetate (Figure 3). Of the fatty acids, physical activity was inversely associated with absolute concentrations of many different types but not with ratios of specific fatty acids to total fatty acids, except monounsaturated fatty acids (Figure 3). Total physical activity was also inversely associated with glycoprotein acetyls (Figure 3)—a marker of inflammation. As shown in Figure I in the Data Supplement, the associations were linear for total cholesterol, triglycerides, and apolipoprotein B, whereas for HDL-C, apolipoprotein A1, creatinine, albumin, and to a lesser extent LDL-C, threshold effects were evident at higher levels of physical activity. In addition, the associations were approximately linear for VLDL and HDL particles, VLDL cholesterol (VLDL-C), HDL-C, VLDL triglycerides, glucose, and glycoprotein acetyls (Figure II in the Data Supplement).

Similar associations were observed when we compared the associations of total physical activity with 8 traits measured by both clinical chemistry and nuclear magnetic resonance (NMR) metabolomics (Figure III in the Data Supplement). Overall, among 18 175 participants with 17 biomarkers measured by clinical chemistry (Figures I and IV in the Data Supplement), there were strong inverse associations of total physical activity with total cholesterol (difference per 4 units [equivalent to 1 hour of moderate physical activity such as brisk walking (3–4 mph)]: −35 [95% CI, −49 to −22] mg/dL), LDL-C (−18 [−25 to −11] mg/dL), total triglycerides (−86 [−982 to −7.5] mg/dL), apolipoprotein B (-0.67 [-0.86 to -0.48] mg/dL), and creatinine (-0.69 [-0.85 to -0.52] μmol/L) and positive associations with HDL-C (4.4 [3.1–5.6] mg/dL) and apolipoprotein A1 (0.66 [0.45-0.87] mg/dL). There was also a positive trend for albumin. Moreover, total physical activity was inversely associated with high-sensitivity CRP (hs-CRP), fibrinogen, cystatin C, alanine aminotransferase, gamma glutamyl transferase, and uric acid and positively associated with aspartate aminotransferase (Figure IV in the Data Supplement). Opposite patterns were observed for the associations between sedentary leisure time and clinical chemistry (Figure V in the Data Supplement).

**Figure 4.**
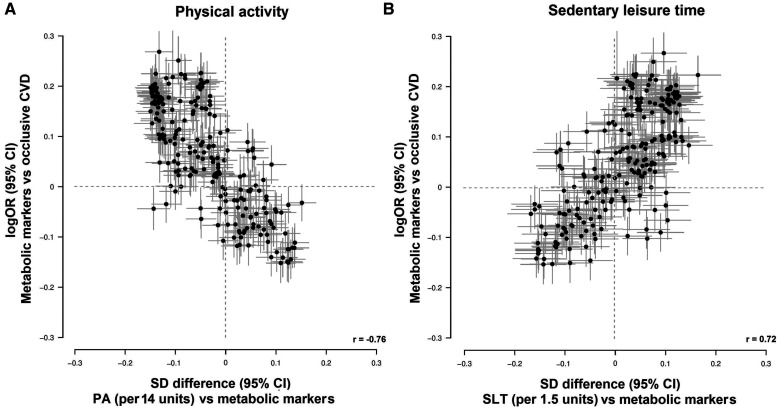
**Global comparison of 1-SD differences of 225 metabolic markers associated with 1-SD higher usual (A) physical activity (PA) and (B) sedentary leisure time (SLT) vs log-odds ratios (ORs) for occlusive cardiovascular disease (CVD) associated with 1-SD higher log-transformed metabolic markers.** Estimates on the y axis are the coefficients of logistic regression of occlusive CVD risk (myocardial infarction and ischemic stroke) on log-transformed metabolic markers. Estimates on the *x* axis are the coefficients of linear regression of log-transformed metabolic markers on (**A**) total PA and (**B**) SLT denotes Pearson correlation coefficient.

In this nested case-control study, total physical activity showed an inverse association with occlusive CVD (odds ratio per 1 SD, 0.86 [0.75–0.99]) with adjustment for age, sex, fasting time, region, smoking, education, income, self-rated health, intake of fresh fruit and red meat, and sedentary leisure time, whereas sedentary leisure time showed a positive association with occlusive CVD (odds ratio per 1 SD, 1.28 [1.02–1.60], adjusting for physical activity plus the same covariates except for sedentary leisure time). There was a clear pattern of the associations of total physical activity with metabolic markers, and of metabolic markers with disease risk; that is, metabolic markers that were associated with higher total physical activity tended to be associated with lower risk of occlusive CVD (Pearson correlation coefficient, −0.76; Figure 4). Further analyses showed that the 18 principal components which explained ≥95% of the variation across the 225 traits potentially explained ≈70% of the effect of total physical activity and ≈50% of the effect of sedentary leisure time on risk of occlusive CVD (Table IV in the Data Supplement). For sedentary leisure time, metabolic markers that associated with higher sedentary leisure time tended to associate with higher risk of occlusive CVD (Pearson correlation coefficient, 0.72; Figure 4).

In sensitivity analyses, the associations with metabolic markers were similar for occupational and nonoccupational physical activity, although the magnitude of effects appeared somewhat stronger for the latter (Figures VI through VIII in the Data Supplement), and heterogeneity tests showed no evidence of any difference except for triglycerides in large HDL (false discovery rate–adjusted *P* for heterogeneity, 0.02). Similar associations were evident (1) when restricting the analyses to controls (Figures IX through XI in the Data Supplement); (2) when further adjusting for body mass index, diabetes mellitus, other dietary factors, or medications (Figures XII through XVII in the Data Supplement); (3) when excluding participants with major chronic diseases or poor self-rated health at baseline (Figures XVIII through XX in the Data Supplement); (4) in all and fasted participants (Figures XXI through XXIII in the Data Supplement); and (5) in both men and women (Figure XXIV). When comparing participants from urban and rural areas (Figures XXV and XXVI in the Data Supplement), the patterns were generally similar except for greater magnitudes of associations for physical activity with several traits in urban areas, including VLDL particles, VLDL-C, triglycerides in VLDL, IDL (intermediate-density lipoprotein), and LDL, apolipoprotein B, isoleucine, leucine, glucose, and glycoprotein acetyls. When comparing lipids and lipoproteins across 10 regions (Figures XXVII and XXVIII in the Data Supplement), there was no evidence of any difference between regions except for triglycerides in IDL and LDL (*P* for heterogeneity, <0.05). When examining the combined effects of high total physical activity and low sedentary leisure time on metabolomics, significant interactions were observed for lipoprotein concentrations of very small VLDL, IDL, LDL (large, medium, and small), cholesterol concentrations in extremely large and very small VLDL, IDL, large LDL, very large HDL, total cholesterol, VLDL-C, LDL-C, apolipoprotein A1, and apolipoprotein B (Figure XXIX in the Data Supplement). In cases where an interaction was present, the combination of high physical activity and low sedentary leisure time had stronger associations with lower concentrations of atherogenic lipoproteins, their cholesterol concentrations, and apolipoprotein B in comparison to other groups.

## Discussion

This study comprehensively examined the associations of self-reported total, occupational, nonoccupational physical activity and sedentary leisure time with a wide range of metabolic biomarkers and the potential role of these biomarkers in explaining the association between physical activity, sedentary leisure time, and risk of major occlusive CVD. In this Chinese population, there were inverse associations of physical activity with VLDL and LDL and positive associations with HDL particle concentrations. In addition, total physical activity was inversely associated with inflammation markers including hs-CRP, fibrinogen, and glycoprotein acetyls. Although the associations of sedentary leisure time with metabolic markers generally mirrored those of total physical activity, a few traits showed different associations (eg, histidine). Importantly, we showed that the global difference in metabolic biomarkers related to higher physical activity conferred lower risk of occlusive CVD, with ≈70% of the protective association potentially explained by these metabolic markers. These metabolic markers also potentially explained ≈50% of the harmful effect of sedentary leisure time on occlusive CVD. These results further extended prospective findings in our study showing inverse associations of physical activity with major vascular events (Figure XXX in the Data Supplement).^2^

To date, 3 observational studies have previously assessed the associations of physical activity with circulating metabolic markers in adult populations.^8,15,16^ While the overall findings were generally similar to those in the present study, they tended to focus on leisure time physical activity and did not examine the association of metabolic markers with disease risk or associations of physical activity with disease risk. In a twin-pair and 3 population-based cohort studies in Finland (16 twin pairs, Pieksämäki cohort, Young Finns Study, and Northern Finland Birth Cohort 1966) with 136 metabolic markers measured using the same NMR platform,^8^ self-reported active individuals had lower concentrations, in comparison with inactive individuals, of the apolipoprotein-B–containing lipoproteins (VLDL, LDL, and IDL) and their cholesterol concentrations, and higher concentrations of large HDL and their cholesterol concentrations but not small HDL. Moreover, physically active individuals had on average lower concentrations of glucose, glycoprotein acetyls, and alanine. The present study showed similar findings for lipoprotein particles, lipid constituents, amino acids, and other metabolic biomarkers. In particular, similar findings for LDL particles and LDL-C were observed when comparing the associations for nonoccupational physical activity in CKB with the associations for leisure-time physical activity in the Finnish study, although the mean levels of LDL-C in our study populations were lower. Consistent with our association of physical activity and HDL particles, randomized controlled trials suggest that exercise training increases large HDL particles and HDL particle size and decreases small HDL particles.^17,18^ Consistent with the Finnish study, the associations between physical activity and metabolomics persisted when additionally adjusting for body mass index, while the present study extended this to show that the associations between physical activity and metabolites persisted even with additional adjustment for diabetes mellitus. As well as showing similar patterns of physical activity as in the Finnish study,^8^ the present study further characterized the associations of sedentary leisure time and showed opposite associations with lipids and lipoproteins, amino acids, and inflammation.

Isoleucine, leucine, and valine are part of the branched-chain amino acid group—essential amino acids that play a key role in energy production and protein synthesis.^19^ Circulating levels of branched-chain amino acids are associated with obesity, insulin resistance, metabolic disorders, type 2 diabetes mellitus, and CVD.^19^ The findings in CKB were directionally consistent with a small Chinese study (n=277) assessing physical activity by accelerometers and a Japanese study (n=1193) by questionnaires, both of which reported inverse associations of total physical activity with isoleucine, leucine, and valine,^15,16^ as well as positive associations of sedentary leisure time with these branched-chain amino acids.

In addition to quantifying the associations of physical activity with metabolic markers, this study examined the extent to which hundreds of metabolic markers potentially explained the associations between physical activity and CVD risk. One study in the United States with 27 000 participants and 979 incident CVD (mainly myocardial infarction and ischemic stroke) showed that conventional lipids (LDL-C, HDL-C, and total cholesterol) and inflammation (hs-CRP, fibrinogen, and soluble intercellular adhesion molecule-1) each accounted for 19% and 32% of the protective effect of leisure-time physical activity on CVD risk.^20^ In CKB, we identified a pattern whereby physical activity tended to be associated with lower concentrations of metabolic markers that were associated with a higher risk of occlusive CVD and vice versa. Compared with the US study, our study reported a larger proportion of this protective effect explained by lipids (LDL-C, HDL-C, and total cholesterol, 34%) and a similar proportion by inflammation (hs-CRP and fibrinogen, 30%). In CKB, a similar proportion of the effect of sedentary leisure time on occlusive CVD risk was potentially explained by inflammation (hs-CRP and fibrinogen, 35%), while a larger proportion was explained by lipids (LDL-C, HDL-C, and total cholesterol, 60%) than physical activity. However, among these metabolic biomarkers assessed, HDL-C, hs-CRP, and fibrinogen are unlikely to be causally associated with CVD, as suggested by Mendelian randomization studies.^21,22^ Atherogenic lipoproteins including VLDL and LDL are almost certainly causally related to CVD, while recent evidence has suggested that glycoprotein acetyls may mark inflammation pathways relevant to the development of CVD.^9^ In our study, glycoprotein acetyls alone potentially explained 25% of the total effect of physical activity on CVD, while VLDL-C and LDL-C each explained 13% and 10%, respectively (Table IV in the Data Supplement). Glycoprotein acetyls are the products of glycosylation modification of secreted inflammatory proteins, which are correlated with other acute phase reactants including CRP and fibrinogen.^10,23^ In our study, the inverse association of total physical activity with glycoprotein acetyls was consistent with the inverse associations with hs-CRP and fibrinogen as measured by clinical chemistry assay, in agreement with previous cross-sectional studies (≈70k participants) in Western populations that assessed leisure-time physical activity.^5–7^ Inflammation pathways may, therefore, represent a promising mechanism to explain the protective association between physical activity and CVD. Indeed, previous studies have reported that higher levels of glycoprotein acetyls were associated with incident type 2 diabetes mellitus, hypertension, and CVD.^10,23^

The associations that we report potentially implicate lipids and lipoproteins as mediators of the beneficial effects of physical activity on risk of CVD. This is plausible because physical activity increases the mitochondrial density in skeletal muscle, leading to higher metabolism of VLDL triglycerides and fatty acids. Notably, these patterns of associations are shown in our data, with higher physical activity associated with lower concentrations of VLDL particles, VLDL-C and VLDL triglycerides, and higher sedentary leisure time associated with higher concentrations of these biomarkers. It is also noteworthy that these associations of physical activity and sedentary leisure time were seen across the lipoprotein and lipid cascade, despite the average LDL-C of our dataset being relatively low (2.4 mmol/L). Consistent with previous observational studies in China^24^ and elsewhere (mean LDL-C, 4.4–4.5 mmol/L),^14^ randomized controlled trials have shown that lowering of LDL-C reduced the risk of major vascular events even among individuals with so-called normal or low cholesterol levels.^25^

Our findings are of contemporary importance given the recent publication of guidelines that seek to increase the amounts of physical activity that individuals undertake to prevent CVD. For example, the Physical Activity Guidelines for Americans advise more physical activity and less sedentary leisure time as both provide health benefits as assessed by all-cause and CVD mortality, providing recommendations on the types and amounts of physical activity across age groups. Of note, no quantitative guideline is provided for sitting time.^11^ More recently, the American College of Cardiology/American Heart Association published guidelines for the primary prevention of CVD, which included advice for adults to engage in at least 150 minutes of moderate-intensity physical activity (or 75 minutes of vigorous-intensity physical activity).^26^ The American College of Cardiology/American Heart Association guidelines also advise decreasing sedentary behavior in adults to lower risk of CVD, but this recommendation is based on limited data. Our study quantifies the associations of physical activity and sitting time with circulating metabolites and CVD in adult populations. More importantly, we showed similar patterns for occupational and nonoccupational physical activity, which reinforced current Chinese guidelines that promote any type of physical activity for CVD prevention.^27^

The strengths of the CKB included large numbers of well-characterized CVD events, assessment of different activity measures (ie, total, occupational, nonoccupational physical activity, and sedentary leisure time) with a broad range of blood-based metabolic markers, and the excellent concordance of NMR measurements compared with conventional clinical chemistry approaches.^10^ Our study also had several limitations. First, although similar associations were observed after further adjustment for possible confounders or mediators including body mass index, diabetes mellitus, and dietary variables, residual confounding because of unmeasured or suboptimally measured factors (eg, diet) may still be present. Second, physical activity and sedentary leisure were self-reported, and, therefore, measurement error may exist because of subjective reporting and between-person differences in intensity of physical activity. However, we corrected for regression dilution using the resurvey data when assessing the associations of physical activity and sedentary leisure time with metabolic markers, which may partially account for random measurement error and within-person variability. In addition, the patterns between total physical activity and metabolomics observed in the current study were generally consistent with a recent study of 1826 adolescents assessing device-measured physical activity and NMR metabolomics, particularly for cholesterol and triglycerides in HDL and VLDL and glycoprotein acetyls.^28^ That study also showed opposite associations for sedentary leisure time, but the associations for other metabolic biomarkers were weaker or nonsignificant compared with those in CKB. Third, we used principal component analysis to account for the large number of correlated metabolic markers when we calculated the proportion of associations of physical activity and sedentary leisure time with CVD potentially explained by all metabolic markers. Therefore, it is difficult to fully disentangle which biomarkers, either individually or in combination, chiefly explained the effects of physical activity on CVD, especially given that the associations of physical activity and metabolic biomarkers were based on cross-sectional analyses.

## Conclusions

Our study set in China shows that higher physical activity was associated with lower concentrations of atherogenic lipoproteins and cholesterol, and lower levels of inflammation, and in general, these metabolic markers were associated with risk of occlusive CVD.^10^ Opposing patterns of associations between sedentary leisure time and metabolic markers were observed. This suggests that physical activity may result in favorable alterations to blood-based lipids and metabolic markers that might partly explain the relationship between physical activity and CVD. Our findings provide insights into the biological mechanisms linking physical activity, sedentary leisure time, and CVD.

## Acknowledgments

The chief acknowledgment is to the participants, the project staff, and the China National Centre for Disease Control and Prevention and its regional offices for access to death and disease registries. The Chinese National Health Insurance scheme provided electronic linkage to all hospital admission data.

## Sources of Funding

Baseline survey: Kadoorie Charitable Foundation, Hong Kong. Long-term continuation: UK Wellcome Trust (088158/Z/09/Z, 104085/Z/14/Z), Chinese Ministry of Science and Technology (2011BAI09B01, 2012-14), Chinese National Natural Science Foundation (81390541). The British Heart Foundation, UK Medical Research Council, and Cancer Research UK provide core funding to the Oxford Clinical Trial Service Unit and Epidemiological Studies Unit. Nuclear magnetic resonance metabolomics was supported by the British Heart Foundation Centre of Research Excellence, Oxford (RE/13/1/30181). F. Bragg acknowledges support from the BHF Centre of Research Excellence, Oxford. Dr Holmes is supported by a British Heart Foundation Intermediate Clinical Research Fellowship (FS/18/23/33512) and the National Institute for Health Research Oxford Biomedical Research Centre.

## Disclosures

None.

## Supplementary Material
