# Supplementary material for: Physical Activity, Sedentary Leisure Time, Circulating Metabolic Markers, and Risk of Major Vascular Diseases
Source: Circ Genom Precis Med. 2019 Sep 17;12(9):e002527. doi: 10.1161/CIRCGEN.118.002527 (PMC6752700; doi:10.1161/CIRCGEN.118.002527)
Supplement: Supplementary file 1 [file hcg-12-e002527-s001.pdf]

SUPPLEMENTAL MATERIAL

Methods

Study population

The study population was a subset of the CKB, which is a prospective cohort study of 512,713 adults aged 30-79 years, recruited in 2004-08 from 10 (five urban and five rural) geographically-defined areas in China. Details of the CKB design, survey methods and long-term follow-up have been previously described.[1] All participants eligible for this study had completed a written informed consent form. Briefly, at the baseline survey participants completed an interviewer-administered laptop-based questionnaire on socio-demographic characteristics, smoking, alcohol consumption, diet, physical activity, and medical history, and underwent a range of physical measurements, including height, weight, hip and waist circumference, bio-impedance, lung function, blood pressure, and heart rate. All participants provided a 10mL non-fasting (with time since last meal recorded) blood sample for immediate on-site test of random plasma glucose (RPG) and long-term storage.

A nested case-control study of CVD subtypes was conducted within CKB, involving 912, 1145, and 1138 incident cases of myocardial infarction (MI, ICD-10 I21-23), ischaemic stroke (IS, I63 and I69.3), and intracerebral haemorrhage (ICH, I61), respectively, with a censoring date of 1 January 2015. Common controls within CKB were frequency matched, where possible, to the combined cases by age, sex, and area. Cases and controls were free of prior vascular disease (including absence of lipid-lowering therapy with statin and other drugs) and cancer. Controls were further selected from individuals who were alive and free of stroke of any type, MI, or CHD by the censoring date. These incident cases were identified through electronic linkage via a unique personal identification number to established death registries and the universal nationwide health insurance system, which provides coded disease diagnoses (clinical description and ICD-10 codes) and procedures (e.g. coronary artery bypass grafting, percutaneous coronary intervention) for each hospitalisation of participants

on a 6-monthly basis. A total of 4660 participants (3195 CVD cases and 1465 controls) were included in the main analysis.

Nested case-control study of clinical biochemistry

Cases and controls were originally selected with a censoring date of 1 January 2015, among the CKB participants who: a) were aged between 30-79 years at the baseline survey (2004-2008); b) did not report a doctor-diagnosed coronary heart disease (CHD), stroke, transient ischaemic attack, and cancer at baseline; and c) did not report use of statins. For the nested case-control study, 5486 incident IS, 5067 incident ICH, 1008 incident MI, and 277 incident fatal ischaemic heart disease (non-MI) cases were selected from individuals with those events. IS and ICH cases were aged below 71 years at the time of event. Stroke cases were based on the first stroke events. Approximately 90% of all stroke cases in CKB were diagnosed for stroke type with brain imaging (CT scan or MRI).[2] MI cases selected were the youngest at age of event. Controls (n=6343) were selected from individuals who were free of stroke of any type, or unspecified type, MI, or CHD by the censoring date.

All 17 biomarkers were assayed using 1 mL plasma samples collected at baseline. Plasma biochemistry measurements were conducted at the CTSU laboratory which has been accredited (International Organisation for Standardisation 17025:2005) for many of the routine tests. Lipid-related biomarkers were measured directly using biochemistry analyser Beckman Coulter AU680 (Beckman Coulter Inc., UK).

Measurement of physical activity and sedentary leisure time

At baseline, and subsequent resurveys, participants were asked about their regular type and duration of physical activities related to occupation, commuting, housework, and leisure-time exercise during the past year. To quantify the amount of physical activity, metabolic equivalent of tasks (METs) from the 2011 update of a major compendium of physical

activities were used.[3] The MET value for a particular type of physical activity represents the ratio of the energy expended per kilogram of body weight per hour during that activity to that expended when sitting quietly. The number of hours spent per day participating in each activity was multiplied by the MET value for that activity, and the daily amount of total physical activity was obtained by summing the MET-hours/day for activities related to occupation and non-occupational (i.e. commuting, housework, and non-sedentary leisure time) activities. Hours spent per day on sedentary leisure time activities (such as television watching and reading) and sleeping were also recorded, but were not included in the calculation of total physical activity.

Blood assays

For each participant, a 10-ml non-fasting blood sample (with time of last meal recorded) was collected into one EDTA vacutainer (BD Hemogard™, USA). The samples were then kept in a portable, insulated cool box with ice packs (to maintain their temperature at 0-4℃) for up to a few hours before being taken to the local study laboratory for immediate processing. After centrifuging and aliquoting, the four cryovials (including one DNA-containing buffy coat) from each blood sample were stored in a -40℃ freezer for 3-4 months, before being couriered on dry ice to the central blood repository in Beijing for storage at -80℃. Every 6 months, two frozen aliquots of plasma sample from each participant were couriered on dry ice from Beijing to Oxford for long-term storage in liquid nitrogen tanks. The same processes were carried out in urban and rural areas.

The stored baseline plasma samples for the cases and controls were retrieved, thawed and sub-aliquoted at the Wolfson laboratory, CTSU, and 100 uL aliquots were shipped on dry ice to the Brainshake Laboratory at Oulu, Finland, for NMR spectroscopy to quantify 147 circulating metabolites and 78 derived traits in a single experimental run. Samples from

cases and controls were analysed in random order, with laboratory staff blinded to case or control status.

Eight traits (total cholesterol, LDL-C, HDL-C, triglycerides [TGs], apolipoprotein B, apolipoprotein-A1, albumin, and creatinine) covered by NMR spectroscopy were also quantified using standard clinical biochemistry assays at the Wolfson Laboratory, CTSU, University of Oxford, UK, which included the same 4660 samples for NMR metabolomics plus an additional 13,515 samples in a larger nested case-control study of 17 biomarkers (the above mentioned eight plus lipoprotein (a), high-sensitivity C-reactive protein [hs-CRP], fibrinogen, cystatin C, alanine aminotransferase [ALT], aspartate aminotransferase [AST], gamma glutamyl transferase [GGT], 25-hydroxyvitamin D (25(OH)D), and uric acid.

Statistical analysis

All blood biomarkers were log-transformed and then standardised to have a standard deviation (SD) of 1. In the analysis of physical activity and sedentary leisure time with metabolic markers, inverse probability of sampling weighting was used to account for the sampling scheme for the nested case-control study. Linear regression was used to assess the associations of physical activity and sedentary leisure time with metabolic markers, adjusted for age (continuous), sex, fasting time (continuous), region (10 regions), smoking status (4 categories: never regular, occasional, former regular, or current regular), education (6 categories: no formal school, primary school, middle school, high school, technical school/college, or university), income (6 categories: <2500, 2500-4999, 5000-9999, 10,000-19,999, 20,000-34,999, or ≥35,000 RMB/year), self-rated health (4 categories: excellent, good, fair, or poor), and intake of fruit and red meat (5 categories: daily, 4-6 days/week, 1-3 days/week, monthly, never/rarely) as the basic adjustment. All analyses relating to physical activity were adjusted for sedentary leisure time and vice versa (i.e. analyses relating to sedentary leisure time were adjusted for physical activity). For each biomarker, adjusted SD differences of log-transformed metabolic markers and 95% confidence intervals (CI)

associated with 1-SD (14 MET-h/day) higher usual physical activity and with 1-SD (1.5 h/day) higher sedentary leisure time were estimated, adjusting for regression dilution using repeated measures of physical activity and sedentary leisure time in resurvey. For fatty acids, in addition to the absolute concentrations, we used the ratio of individual fatty acids to total fatty acids as this may account for the potential confounding effects of TGs. To account for the large number of highly correlated metabolic markers, we used false discovery rate-corrected (FDR) *p*-values <0.05 calculated using the Benjamini-Hochberg method.[4] To assess the associations of physical activity and sedentary leisure time with metabolic markers, restricted cubic splines for total physical activity were calculated using three fixed knots at 10, 50, and 90% throughout the range (**Supplementary Figure 1** and **Supplementary Figure 2**). Logistic regression was used to estimate odds ratios (ORs) of occlusive CVD (i.e. MI and IS) per 1-SD higher log-transformed metabolic markers (for all 225 measurements), with the same variables adjusted for as in the analysis of physical activity and metabolic markers. ICH cases were excluded from the analysis of metabolic markers and occlusive CVD because of its lack of associations with metabolic markers.[5] Adjusted log ORs were then plotted against SD differences in corresponding metabolic biomarkers per 1-SD higher physical activity and sedentary leisure time. The Pearson correlation coefficients of the associations of physical activity and sedentary leisure time with metabolic biomarkers and of metabolic biomarkers with occlusive CVD were calculated. To approximate the extent to which the NMR-spectroscopy derived traits potentially explained the association between physical activity and occlusive CVD, we simultaneously included 18 principal components (PCs) that explained ≥ 95% of the variation across the 225 traits in the basic model and examined the percent change in the log ORs per 1-SD higher usual physical activity and sedentary leisure time. The proportion of occlusive CVD risk reduction explained by the 18 PCs was calculated as follows: $((\text{logOR}_{\text{basic model}} - \text{logOR}_{\text{adjusted model}}) / (\text{logOR}_{\text{basic model}})) \times 100\%$. Statistical analysis was performed using R version 2.14.2.

Inverse probability weighting

In the analysis of physical activity and metabolic markers, inverse probability of sampling weights (i.e. inclusion in the nested case-control study) were developed to ensure that our analysis accounted for the inclusion/exclusion criteria and sampling scheme for the nested case-control study. Cases and controls were assigned different weights to reflect the different proportions of cases and controls from eligible participants in the entire CKB cohort. The weights were calculated separately for controls and cases as the number of eligible participants divided by the number selected in the nested case-control study. The weights were 307.35 for controls, 4.47 for MI cases, 27.82 for IS cases, and 6.78 for ICH cases.

Sensitivity analyses

We conducted several sensitivity analyses. First, separate analyses were conducted for occupational and non-occupational physical activity. Second, additional adjustments were made for body mass index (BMI, continuous), prevalent diabetes, intake frequency of other dietary factors (i.e. rice, wheat, staple food other than rice and wheat [e.g. corn, millet], poultry, fish/seafood, dairy, eggs, soybean, preserved vegetables [2 categories: daily or less than daily]), and medications (anti-hypertensive and anti-diabetic medications [2 categories: yes or no]). Anti-hypertensive medications included aspirin, angiotensin converting enzyme inhibitor [ACE inhibitor], beta-blocker, diuretics, and calcium channel blocker, while anti-diabetic medications included chlorpropamide or metformin, and insulin). Third, we repeated the analyses of total physical activity and sedentary leisure time with metabolomics excluding participants who self-reported a history of major chronic diseases (diabetes, hypertension, rheumatic heart disease, tuberculosis, emphysema/bronchitis, asthma, cirrhosis/chronic hepatitis, peptic ulcer, gallstone/gallbladder disease, kidney disease) and who reported poor self-rated heath at baseline. Fourth, separate analyses were conducted comparing all and fasted (≥8 hours) participants. Fifth, instead of adjusting for the 10 regions as a categorical variable, we included a random intercept for region. Lastly, we tested for interactions of high

total physical activity and low sedentary leisure time on metabolomics. We created binary variables for high physical activity (≥20 MET-h/day [median]) and low sedentary leisure time (<3 h/day [median]), and included high physical activity, low sedentary leisure time, an interaction term between these two binary variables, and other covariates in a model. From this model we obtained SD differences of metabolic markers for three groups (high physical activity and high sedentary leisure time, low physical activity and low sedentary leisure time, high physical activity and low sedentary leisure time), compared with the reference group (low physical activity and high sedentary leisure time). Model 1 included all covariates in the main model plus high physical activity and low sedentary leisure time. Model 2 included variables in model 1 plus an interaction term for high physical activity and low sedentary leisure time. The likelihood ratio test was used to compare model 1 and model 2.

Regression dilution

After completion of the baseline survey, 5 to 6% of the original participants were selected randomly for a resurvey, which took place from July through October 2008. Similar procedures were used to those at baseline. To adjust for regression dilution bias, we calculated the correlation between physical activity at baseline visit and physical activity measured at resurvey among 19,338 participants. Separate correlation coefficients were calculated for total, occupational, non-occupational physical activity, and sedentary leisure time. The regression dilution ratios were 0.52 for total physical activity, 0.59 for occupational physical activity, 0.48 for non-occupational physical activity, and 0.34 for sedentary leisure time. SD difference and log OR estimates (and corresponding SDs) per 14 units higher physical activity and per 1.5 units higher sedentary leisure time were divided by this correlation to obtain regression dilution-adjusted estimates.[6]

References

1. Chen Z, Chen J, Collins R, Guo Y, Peto R, Wu F, Li L; China Kadoorie Biobank (CKB) collaborative group. China Kadoorie Biobank of 0.5 million people: survey methods, baseline characteristics and long-term follow-up. *Int J Epidemiol.* 2011;40:1652-1666.

2. Lacey B, Lewington S, Clarke R, et al. Age-specific association between blood pressure and vascular and non-vascular chronic diseases in 0.5 million adults in China: a prospective cohort study. *Lancet Glob Health* 2018; 6(6): 641-49.

3. Ainsworth BE, Haskell WL, Herrmann SD, Meckes N, Bassett DR Jr, Tudor-Locke C, Greer JL, Vezina J, Whitt-Glover MC, Leon AS. 2011 Compendium of Physical Activities: a second update of codes and MET values. *Med Sci Sports Exerc.* 2011;43:1575-1581.

4. Benjamini Y, Hochberg Y. Controlling the false discovery rate: a practical and powerful approach to multiple testing. *Journal of the Royal Statistical Society Series B (Methodological).* 1995:289-300.

5. Holmes MV, Millwood IY, Kartsonaki C, Hill MR, Bennett DA, Boxall R, Guo Y, Xu X, Bian Z, Hu R, et al. Lipids, lipoproteins, and metabolites and risk of myocardial infarction and stroke. *J Am Coll Cardiol.* 2018;71:620-632.

6. Clarke R, Shipley M, Lewington S, Youngman L, Collins R, Marmont M, et al. Underestimation of risk associations due to regression dilution in long-term follow-up of prospective studies. *Am J Epidemiol* 1999; 150(4): 341-53.

Supplemental Table 1. Daily dietary intake frequency of participants in the nested case-control study among participants without prior CVD

Variable*	MI/IS cases (n=2057)	ICH cases (n=1138)	Controls (n=1465)	All (n=4660)
**Daily consumption, %**				
Fresh fruit	12.8	11.9	15.1	13.3
Fresh vegetables	93.2	92.8	92.4	93.3
Red meat	26.8	24.6	23.6	25.8
Poultry	0.3	0.4	0.4	0.4
Fish/seafood	1.6	1.2	2.0	1.7
Dairy products	7.5	5.3	7.0	7.0
Fresh eggs	13.8	13.1	14.5	14.1
Soybean products	3.5	2.7	2.8	3.2
Rice	52.5	51.7	52.6	52.7
Wheat products	57.0	57.3	54.0	56.2
Other staple food†	20.7	21.1	21.5	21.0
Preserved vegetables	11.4	12.2	13.1	12.4

* Results were standardised by age, sex, and area (where appropriate).
† Other staple food referred to food other than rice and wheat (e.g. corn, millet).
Abbreviations: ICH=intracerebral haemorrhage, IS=ischemic stroke, MI=myocardial infarction.

Supplemental Table 2. Baseline characteristics of participants in the nested case-control study among participants without prior CVD

Variable[*]	CVD cases (n=3195)	Controls (n=1465)	All (n=4660)
Age (SD), year	46.8 (7.7)	45.0 (8.3)	46.2 (8.0)
Female, %	51.0	52.3	50.2
**Socioeconomic and lifestyle factors, %**			
Urban residents	31.0	24.9	29.1
≥9 years of education	21.1	24.4	22.1
Household income ≥35,000 RMB/year	12.0	10.4	11.5
Ever regular smoking			
Male	74.0	67.8	72.5
Female	2.1	1.8	2.1
Weekly alcohol intake			
Male	34.4	33.3	34.4
Female	2.2	1.6	2.0
Total physical activity (SD), MET h/day	22.7 (14.3)	24.6 (14.5)	23.0 (14.4)
Sedentary leisure time (SD), h/d	3.1 (1.5)	3.0 (1.4)	3.1 (1.5)
**Blood pressure, blood glucose and anthropometry**			
SBP (SD), mmHg	141.7 (27.3)	126.8 (17.8)	137.3 (25.7)
RPG (SD), mmol/L	6.3 (3.1)	5.6 (1.9)	6.1 (2.8)
BMI (SD), kg/m^2	24.2 (3.6)	23.4 (3.2)	24.0 (3.5)
**Prior disease history, %**			
Hypertension	17.9	5.5	14.3
Diabetes	7.6	3.1	6.5
**Family disease history, %**			
Diabetes	6.7	5.4	6.3
CVD	13.8	17.3	14.2

[*] Results were standardised by age, sex, and area (where appropriate). Values are means unless otherwise stated.
Abbreviations: %BF=percent body fat, BMI=body mass index, CHD=coronary heart disease, CVD=cardiovascular disease, ICH=intracerebral haemorrhage, IS=ischemic stroke, MET=metabolic equivalent of task, MI=myocardial infarction, RPG=random plasma glucose, SBP=systolic blood pressure, TIA=transient ischaemic attack.

Supplemental Table 3. Associations of usual total physical activity and sedentary leisure time with all 225 metabolic biomarkers

		Total physical activity				Sedentary leisure time			
Metabolic biomarker	Unit	Coefficient	SE	*p*-value	FDR-adjusted *p*-value	Coefficient	SE	*p*-value	FDR-adjusted *p*-value
Acetoacetate	mmol/l	-0.127	0.027	3.20E-06	3.13E-05	8.0E-02	4.3E-02	6.21E-02	9.50E-02
Acetate	mmol/l	0.056	0.027	3.69E-02	6.33E-02	-5.0E-03	4.2E-02	9.04E-01	9.29E-01
Alanine	mmol/l	-0.092	0.030	2.44E-03	5.72E-03	1.2E-01	4.7E-02	1.27E-02	2.60E-02
Albumin	signal area	0.013	0.028	6.35E-01	6.97E-01	-2.6E-02	4.4E-02	5.63E-01	6.07E-01
Apolipoprotein A-I	g/l	-0.010	0.029	7.33E-01	7.74E-01	-1.3E-01	4.5E-02	3.54E-03	9.37E-03
Apolipoprotein B	g/l	-0.137	0.028	1.10E-06	2.21E-05	1.9E-01	4.4E-02	1.46E-05	1.31E-04
Ratio of apolipoprotein B to apolipoprotein A-I	%	-0.131	0.028	3.96E-06	3.71E-05	2.5E-01	4.5E-02	3.15E-08	7.09E-06
3-hydroxybutyrate	mmol/l	-0.043	0.025	8.95E-02	1.40E-01	7.5E-02	3.9E-02	5.53E-02	8.82E-02
Citrate	mmol/l	0.008	0.028	7.75E-01	8.15E-01	-9.5E-02	4.4E-02	3.30E-02	5.80E-02
Creatinine	mmol/l	-0.066	0.022	2.99E-03	6.86E-03	2.7E-02	3.5E-02	4.44E-01	4.94E-01
22:6, docosahexaenoic acid	mmol/l	-0.056	0.027	4.29E-02	7.25E-02	8.6E-02	4.3E-02	4.42E-02	7.32E-02
Ratio of 22:6 docosahexaenoic acid to total fatty acids	%	0.121	0.029	3.71E-05	1.60E-04	-4.7E-02	4.6E-02	3.03E-01	3.55E-01
Esterified cholesterol	mmol/l	-0.110	0.031	3.78E-04	1.18E-03	1.3E-02	4.8E-02	7.92E-01	8.21E-01
Omega-3 fatty acids	mmol/l	-0.068	0.027	1.19E-02	2.33E-02	8.1E-02	4.3E-02	5.68E-02	8.97E-02
Ratio of omega-3 fatty acids to total fatty acids	%	0.074	0.027	6.72E-03	1.44E-02	-1.9E-02	4.3E-02	6.62E-01	7.06E-01
Omega-6 fatty acids	mmol/l	-0.124	0.028	1.10E-05	7.10E-05	8.1E-02	4.4E-02	6.52E-02	9.91E-02
Ratio of omega-6 fatty acids to total fatty acids	%	0.025	0.030	4.09E-01	4.76E-01	-7.1E-02	4.7E-02	1.30E-01	1.74E-01
Free cholesterol	mmol/l	-0.051	0.029	8.27E-02	1.31E-01	8.7E-02	4.6E-02	6.03E-02	9.29E-02
Glucose	mmol/l	-0.096	0.026	2.35E-04	7.78E-04	5.3E-02	4.1E-02	1.97E-01	2.47E-01
Glutamine	mmol/l	0.075	0.029	8.50E-03	1.71E-02	-1.7E-01	4.5E-02	1.90E-04	9.52E-04
Glycoprotein acetyls, mainly a1-acid glycoprotein	mmol/l	-0.132	0.027	1.43E-06	2.21E-05	1.6E-01	4.3E-02	2.86E-04	1.31E-03
Total cholesterol in HDL	mmol/l	0.072	0.029	1.26E-02	2.42E-02	-2.1E-01	4.5E-02	5.97E-06	6.72E-05
Mean diameter for HDL particles	nm	0.107	0.029	2.59E-04	8.46E-04	-1.8E-01	4.6E-02	9.30E-05	5.17E-04
Triglycerides in HDL	mmol/l	-0.078	0.029	7.49E-03	1.58E-02	1.2E-01	4.6E-02	9.72E-03	2.04E-02
Total cholesterol in HDL2	mmol/l	0.078	0.029	7.69E-03	1.60E-02	-2.1E-01	4.6E-02	5.54E-06	6.56E-05
Total cholesterol in HDL3	mmol/l	0.058	0.028	4.06E-02	6.92E-02	-9.5E-02	4.5E-02	3.35E-02	5.84E-02
Histidine	mmol/l	-0.004	0.030	9.01E-01	9.14E-01	-2.4E-01	4.8E-02	7.00E-07	1.95E-05
Total cholesterol in IDL	mmol/l	-0.032	0.029	2.71E-01	3.48E-01	8.4E-02	4.6E-02	6.73E-02	1.01E-01

Total cholesterol to total lipids ratio in IDL	%	-0.013	0.030	6.48E-01	7.01E-01	-3.1E-02	4.6E-02	5.06E-01	5.51E-01
Cholesterol esters in IDL	mmol/l	-0.048	0.029	9.75E-02	1.49E-01	9.3E-02	4.5E-02	4.17E-02	7.06E-02
Cholesterol esters to total lipids ratio in IDL	%	-0.095	0.030	1.50E-03	3.67E-03	1.1E-02	4.7E-02	8.13E-01	8.39E-01
Free cholesterol in IDL	mmol/l	0.005	0.029	8.77E-01	8.97E-01	5.8E-02	4.6E-02	2.07E-01	2.56E-01
Free cholesterol to total lipids ratio in IDL	%	0.083	0.030	5.75E-03	1.24E-02	-4.2E-02	4.7E-02	3.75E-01	4.30E-01
Total lipids in IDL	mmol/l	-0.045	0.028	1.15E-01	1.73E-01	1.0E-01	4.5E-02	2.19E-02	4.04E-02
Concentration of IDL particles	mol/l	-0.055	0.028	4.74E-02	7.95E-02	1.2E-01	4.3E-02	5.61E-03	1.33E-02
Phospholipids in IDL	mmol/l	-0.028	0.029	3.36E-01	4.13E-01	9.1E-02	4.6E-02	4.51E-02	7.37E-02
Phospholipids to total lipds ratio in IDL	%	0.071	0.029	1.28E-02	2.43E-02	-8.5E-02	4.5E-02	5.79E-02	9.05E-02
Triglycerides in IDL	mmol/l	-0.052	0.029	7.48E-02	1.21E-01	1.6E-01	4.6E-02	3.82E-04	1.62E-03
Triglycerides to total lipids ratio in IDL	%	-0.015	0.029	5.91E-01	6.59E-01	6.5E-02	4.5E-02	1.46E-01	1.90E-01
Isoleucine	mmol/l	-0.045	0.028	1.14E-01	1.73E-01	7.7E-02	4.5E-02	8.46E-02	1.19E-01
Total cholesterol in large HDL	mmol/l	0.108	0.029	1.70E-04	5.87E-04	-2.3E-01	4.5E-02	2.29E-07	1.03E-05
Total cholesterol to total lipids ratio in large HDL	%	0.141	0.028	5.15E-07	1.93E-05	-2.2E-01	4.4E-02	4.05E-07	1.52E-05
Cholesterol esters in large HDL	mmol/l	0.106	0.029	2.21E-04	7.42E-04	-2.4E-01	4.5E-02	1.62E-07	1.03E-05
Cholesterol esters to total lipids ratio in large HDL	%	0.113	0.028	6.07E-05	2.36E-04	-2.2E-01	4.4E-02	5.46E-07	1.75E-05
Free cholesterol in large HDL	mmol/l	0.120	0.029	3.17E-05	1.40E-04	-2.2E-01	4.5E-02	1.19E-06	2.42E-05
Free cholesterol to total lipids ratio in large HDL	%	0.160	0.031	3.15E-07	1.61E-05	-1.3E-01	4.9E-02	6.91E-03	1.55E-02
Total lipids in large HDL	mmol/l	0.096	0.029	9.27E-04	2.51E-03	-2.2E-01	4.5E-02	7.79E-07	1.95E-05
Concentration of large HDL particles	mol/l	0.085	0.028	2.75E-03	6.37E-03	-2.1E-01	4.5E-02	1.85E-06	2.60E-05
Phospholipids in large HDL	mmol/l	0.092	0.029	1.54E-03	3.73E-03	-2.2E-01	4.6E-02	8.72E-07	1.96E-05
Phospholipids to total lipds ratio in large HDL	%	-0.102	0.029	4.57E-04	1.35E-03	1.4E-01	4.5E-02	1.60E-03	4.99E-03
Triglycerides in large HDL	mmol/l	0.034	0.027	2.19E-01	2.92E-01	-3.6E-02	4.3E-02	3.98E-01	4.50E-01
Triglycerides to total lipids ratio in large HDL	%	-0.044	0.029	1.27E-01	1.88E-01	1.4E-01	4.5E-02	2.08E-03	6.01E-03
Total cholesterol in large LDL	mmol/l	-0.037	0.030	2.18E-01	2.91E-01	1.1E-01	4.8E-02	2.17E-02	4.04E-02
Total cholesterol to total lipids ratio in large LDL	%	-0.053	0.031	8.19E-02	1.31E-01	2.3E-03	4.8E-02	9.63E-01	9.76E-01
Cholesterol esters in large LDL	mmol/l	-0.057	0.030	6.33E-02	1.05E-01	1.1E-01	4.8E-02	2.10E-02	3.97E-02
Cholesterol esters to total lipids ratio in large LDL	%	-0.128	0.038	7.83E-04	2.20E-03	2.6E-02	6.0E-02	6.68E-01	7.09E-01
Free cholesterol in large LDL	mmol/l	-0.008	0.030	7.82E-01	8.18E-01	8.5E-02	4.7E-02	6.89E-02	1.01E-01
Free cholesterol to total lipids ratio in large LDL	%	0.137	0.028	7.59E-07	2.14E-05	-1.5E-01	4.3E-02	3.74E-04	1.62E-03
Total lipids in large LDL	mmol/l	-0.029	0.031	3.55E-01	4.27E-01	1.2E-01	4.8E-02	1.02E-02	2.11E-02
Concentration of large LDL particles	mol/l	-0.065	0.028	1.85E-02	3.41E-02	1.3E-01	4.4E-02	2.69E-03	7.37E-03
Phospholipids in large LDL	mmol/l	-0.037	0.031	2.26E-01	2.99E-01	1.3E-01	4.8E-02	8.96E-03	1.90E-02
Phospholipids to total lipds ratio in large LDL	%	0.041	0.030	1.73E-01	2.41E-01	-9.0E-02	4.7E-02	5.70E-02	8.97E-02

Triglycerides in large LDL	mmol/l	-0.026	0.030	3.87E-01	4.58E-01	1.6E-01	4.7E-02	4.78E-04	1.85E-03
Triglycerides to total lipids ratio in large LDL	%	0.024	0.028	4.04E-01	4.74E-01	3.9E-02	4.5E-02	3.86E-01	4.38E-01
Total cholesterol in large VLDL	mmol/l	-0.137	0.030	4.76E-06	4.28E-05	1.8E-01	4.7E-02	1.48E-04	7.89E-04
Total cholesterol to total lipids ratio in large VLDL	%	-0.050	0.029	8.88E-02	1.40E-01	1.5E-01	4.6E-02	9.91E-04	3.28E-03
Cholesterol esters in large VLDL	mmol/l	-0.135	0.030	5.43E-06	4.36E-05	1.9E-01	4.6E-02	3.53E-05	2.64E-04
Cholesterol esters to total lipids ratio in large VLDL	%	0.042	0.025	9.12E-02	1.41E-01	7.8E-02	3.9E-02	4.38E-02	7.32E-02
Free cholesterol in large VLDL	mmol/l	-0.135	0.030	5.60E-06	4.36E-05	1.6E-01	4.7E-02	4.58E-04	1.81E-03
Free cholesterol to total lipids ratio in large VLDL	%	-0.105	0.033	1.25E-03	3.24E-03	1.1E-01	5.1E-02	3.68E-02	6.37E-02
Total lipids in large VLDL	mmol/l	-0.141	0.030	2.97E-06	3.04E-05	1.6E-01	4.7E-02	8.90E-04	3.04E-03
Concentration of large VLDL particles	mol/l	-0.139	0.029	1.35E-06	2.21E-05	1.6E-01	4.5E-02	4.02E-04	1.65E-03
Phospholipids in large VLDL	mmol/l	-0.142	0.030	1.72E-06	2.42E-05	1.7E-01	4.7E-02	2.40E-04	1.15E-03
Phospholipids to total lipds ratio in large VLDL	%	-0.102	0.031	9.99E-04	2.68E-03	1.4E-01	4.9E-02	4.22E-03	1.07E-02
Triglycerides in large VLDL	mmol/l	-0.143	0.030	1.86E-06	2.46E-05	1.6E-01	4.7E-02	8.81E-04	3.04E-03
Triglycerides to total lipids ratio in large VLDL	%	0.057	0.030	6.07E-02	1.01E-01	-1.7E-01	4.8E-02	3.46E-04	1.53E-03
18:2, linoleic acid	mmol/l	-0.087	0.025	4.47E-04	1.34E-03	2.7E-02	3.9E-02	4.86E-01	5.33E-01
Ratio of 18:2 linoleic acid to total fatty acids	%	-0.021	0.023	3.60E-01	4.29E-01	-4.5E-02	3.6E-02	2.12E-01	2.60E-01
Lactate	mmol/l	-0.072	0.029	1.26E-02	2.42E-02	1.3E-01	4.5E-02	3.67E-03	9.60E-03
Total cholesterol in LDL	mmol/l	-0.050	0.030	9.13E-02	1.41E-01	1.0E-01	4.6E-02	2.90E-02	5.21E-02
Mean diameter for LDL particles	nm	0.115	0.029	5.88E-05	2.32E-04	-7.0E-02	4.5E-02	1.18E-01	1.59E-01
Triglycerides in LDL	mmol/l	-0.067	0.029	2.14E-02	3.85E-02	1.6E-01	4.5E-02	4.03E-04	1.65E-03
Leucine	mmol/l	-0.038	0.028	1.70E-01	2.37E-01	4.4E-02	4.4E-02	3.20E-01	3.73E-01
Total cholesterol in medium HDL	mmol/l	0.020	0.031	5.08E-01	5.75E-01	-1.5E-01	4.8E-02	1.67E-03	5.15E-03
Total cholesterol to total lipids ratio in medium HDL	%	0.018	0.029	5.39E-01	6.06E-01	-1.5E-01	4.6E-02	8.07E-04	2.93E-03
Cholesterol esters in medium HDL	mmol/l	0.016	0.031	6.10E-01	6.73E-01	-1.5E-01	4.8E-02	2.00E-03	5.85E-03
Cholesterol esters to total lipids ratio in medium HDL	%	0.005	0.029	8.72E-01	8.96E-01	-1.1E-01	4.6E-02	1.44E-02	2.83E-02
Free cholesterol in medium HDL	mmol/l	0.027	0.030	3.61E-01	4.29E-01	-1.9E-01	4.6E-02	5.29E-05	3.60E-04
Free cholesterol to total lipids ratio in medium HDL	%	0.068	0.030	2.14E-02	3.85E-02	-2.2E-01	4.6E-02	1.65E-06	2.60E-05
Total lipids in medium HDL	mmol/l	0.023	0.032	4.72E-01	5.42E-01	-1.2E-01	5.0E-02	1.91E-02	3.63E-02
Concentration of medium HDL particles	mol/l	-0.013	0.029	6.44E-01	7.00E-01	-1.7E-01	4.6E-02	1.54E-04	7.89E-04
Phospholipids in medium HDL	mmol/l	0.029	0.031	3.54E-01	4.27E-01	-1.3E-01	4.9E-02	8.62E-03	1.86E-02
Phospholipids to total lipds ratio in medium HDL	%	0.059	0.028	3.56E-02	6.16E-02	3.8E-02	4.4E-02	3.83E-01	4.37E-01
Triglycerides in medium HDL	mmol/l	-0.100	0.030	7.97E-04	2.21E-03	1.5E-01	4.7E-02	1.07E-03	3.49E-03
Triglycerides to total lipids ratio in medium HDL	%	-0.101	0.030	6.90E-04	1.97E-03	2.4E-01	4.7E-02	1.84E-07	1.03E-05
Total cholesterol in medium LDL	mmol/l	-0.036	0.031	2.47E-01	3.25E-01	7.8E-02	4.9E-02	1.06E-01	1.46E-01

Total cholesterol to total lipids ratio in medium LDL	%	-0.027	0.028	3.36E-01	4.13E-01	1.8E-02	4.4E-02	6.89E-01	7.25E-01
Cholesterol esters in medium LDL	mmol/l	-0.041	0.030	1.64E-01	2.33E-01	8.5E-02	4.7E-02	6.78E-02	1.01E-01
Cholesterol esters to total lipids ratio in medium LDL	%	-0.026	0.028	3.54E-01	4.27E-01	4.8E-02	4.3E-02	2.65E-01	3.19E-01
Free cholesterol in medium LDL	mmol/l	-0.024	0.032	4.52E-01	5.22E-01	6.6E-02	5.0E-02	1.84E-01	2.32E-01
Free cholesterol to total lipids ratio in medium LDL	%	0.093	0.029	1.31E-03	3.25E-03	-1.6E-01	4.5E-02	6.41E-04	2.44E-03
Total lipids in medium LDL	mmol/l	-0.030	0.032	3.55E-01	4.27E-01	7.8E-02	5.0E-02	1.18E-01	1.59E-01
Concentration of medium LDL particles	mol/l	-0.074	0.028	8.21E-03	1.68E-02	1.4E-01	4.4E-02	1.89E-03	5.62E-03
Phospholipids in medium LDL	mmol/l	-0.040	0.032	2.15E-01	2.91E-01	8.6E-02	5.0E-02	8.79E-02	1.23E-01
Phospholipids to total lipds ratio in medium LDL	%	0.023	0.029	4.35E-01	5.05E-01	-6.3E-02	4.6E-02	1.66E-01	2.12E-01
Triglycerides in medium LDL	mmol/l	-0.031	0.031	3.18E-01	3.97E-01	1.4E-01	4.8E-02	4.09E-03	1.05E-02
Triglycerides to total lipids ratio in medium LDL	%	0.032	0.028	2.54E-01	3.30E-01	3.1E-02	4.4E-02	4.79E-01	5.29E-01
Total cholesterol in medium VLDL	mmol/l	-0.136	0.030	5.05E-06	4.36E-05	2.0E-01	4.7E-02	2.60E-05	2.09E-04
Total cholesterol to total lipids ratio in medium VLDL	%	-0.005	0.030	8.61E-01	8.89E-01	6.5E-02	4.7E-02	1.62E-01	2.07E-01
Cholesterol esters in medium VLDL	mmol/l	-0.128	0.030	1.43E-05	7.68E-05	2.1E-01	4.6E-02	9.67E-06	9.46E-05
Cholesterol esters to total lipids ratio in medium VLDL	%	0.031	0.029	2.80E-01	3.59E-01	3.5E-02	4.5E-02	4.36E-01	4.88E-01
Free cholesterol in medium VLDL	mmol/l	-0.140	0.030	2.15E-06	2.55E-05	1.8E-01	4.6E-02	7.95E-05	4.70E-04
Free cholesterol to total lipids ratio in medium VLDL	%	-0.099	0.031	1.31E-03	3.25E-03	9.3E-02	4.8E-02	5.24E-02	8.42E-02
Total lipids in medium VLDL	mmol/l	-0.141	0.030	2.12E-06	2.55E-05	1.9E-01	4.7E-02	4.93E-05	3.46E-04
Concentration of medium VLDL particles	mol/l	-0.145	0.028	2.76E-07	1.61E-05	1.9E-01	4.4E-02	2.45E-05	2.04E-04
Phospholipids in medium VLDL	mmol/l	-0.142	0.030	1.39E-06	2.21E-05	1.9E-01	4.6E-02	3.48E-05	2.64E-04
Phospholipids to total lipds ratio in medium VLDL	%	0.063	0.030	3.56E-02	6.16E-02	-7.2E-02	4.7E-02	1.27E-01	1.70E-01
Triglycerides in medium VLDL	mmol/l	-0.143	0.030	1.29E-06	2.21E-05	1.8E-01	4.6E-02	7.05E-05	4.41E-04
Triglycerides to total lipids ratio in medium VLDL	%	-0.011	0.029	7.03E-01	7.49E-01	-5.4E-02	4.6E-02	2.34E-01	2.85E-01
Monounsaturated fatty acids; 16:1, 18:1	mmol/l	-0.155	0.029	8.42E-08	9.48E-06	1.5E-01	4.5E-02	8.91E-04	3.04E-03
Ratio of monounsaturated fatty acids to total fatty acids	%	-0.097	0.029	8.74E-04	2.40E-03	1.5E-01	4.5E-02	7.23E-04	2.67E-03
Phosphatidylcholine and other cholines	mmol/l	-0.132	0.034	1.18E-04	4.28E-04	-6.2E-02	5.4E-02	2.50E-01	3.02E-01
Phenylalanine	mmol/l	0.033	0.028	2.51E-01	3.28E-01	-1.5E-02	4.5E-02	7.39E-01	7.74E-01
Polyunsaturated fatty acids	mmol/l	-0.119	0.028	1.83E-05	9.16E-05	8.5E-02	4.4E-02	5.20E-02	8.42E-02
Ratio of polyunsaturated fatty acids to total fatty acids	%	0.041	0.029	1.59E-01	2.30E-01	-7.3E-02	4.6E-02	1.08E-01	1.47E-01
Remnant cholesterol (non-HDL, non-LDL -cholesterol)	mmol/l	-0.111	0.029	1.01E-04	3.82E-04	1.7E-01	4.5E-02	1.51E-04	7.89E-04
Total cholesterol in small HDL	mmol/l	-0.001	0.034	9.68E-01	9.68E-01	3.0E-04	5.3E-02	9.96E-01	1.00E+00
Total cholesterol to total lipids ratio in small HDL	%	0.029	0.028	3.06E-01	3.85E-01	2.2E-03	4.5E-02	9.61E-01	9.76E-01
Cholesterol esters in small HDL	mmol/l	0.004	0.030	9.06E-01	9.14E-01	-6.0E-04	4.7E-02	9.90E-01	9.99E-01
Cholesterol esters to total lipids ratio in small HDL	%	0.021	0.026	4.01E-01	4.73E-01	-8.5E-06	4.0E-02	1.00E+00	1.00E+00

Free cholesterol in small HDL	mmol/l	-0.017	0.034	6.10E-01	6.73E-01	-6.5E-02	5.3E-02	2.23E-01	2.73E-01
Free cholesterol to total lipids ratio in small HDL	%	0.048	0.027	7.57E-02	1.22E-01	-1.3E-01	4.2E-02	2.99E-03	8.02E-03
Total lipids in small HDL	mmol/l	-0.021	0.040	5.92E-01	6.59E-01	4.8E-03	6.2E-02	9.38E-01	9.59E-01
Concentration of small HDL particles	mol/l	-0.067	0.030	2.69E-02	4.72E-02	-2.8E-02	4.7E-02	5.52E-01	5.97E-01
Phospholipids in small HDL	mmol/l	-0.026	0.037	4.88E-01	5.58E-01	-3.8E-02	5.8E-02	5.07E-01	5.51E-01
Phospholipids to total lipds ratio in small HDL	%	-0.007	0.028	7.94E-01	8.27E-01	-9.3E-02	4.4E-02	3.73E-02	6.40E-02
Triglycerides in small HDL	mmol/l	-0.097	0.030	1.30E-03	3.25E-03	2.1E-01	4.7E-02	1.01E-05	9.51E-05
Triglycerides to total lipids ratio in small HDL	%	-0.083	0.030	5.09E-03	1.12E-02	2.2E-01	4.6E-02	1.46E-06	2.60E-05
Total cholesterol in small LDL	mmol/l	-0.043	0.031	1.66E-01	2.34E-01	7.0E-02	4.8E-02	1.47E-01	1.90E-01
Total cholesterol to total lipids ratio in small LDL	%	-0.003	0.028	9.02E-01	9.14E-01	3.9E-02	4.4E-02	3.72E-01	4.29E-01
Cholesterol esters in small LDL	mmol/l	-0.043	0.030	1.48E-01	2.15E-01	8.4E-02	4.6E-02	6.91E-02	1.01E-01
Cholesterol esters to total lipids ratio in small LDL	%	0.003	0.025	9.12E-01	9.16E-01	7.9E-02	4.0E-02	4.52E-02	7.37E-02
Free cholesterol in small LDL	mmol/l	-0.039	0.032	2.16E-01	2.91E-01	5.3E-02	5.0E-02	2.89E-01	3.43E-01
Free cholesterol to total lipids ratio in small LDL	%	0.103	0.029	3.33E-04	1.07E-03	-1.8E-01	4.5E-02	4.17E-05	3.02E-04
Total lipids in small LDL	mmol/l	-0.042	0.032	1.88E-01	2.58E-01	6.4E-02	5.1E-02	2.07E-01	2.56E-01
Concentration of small LDL particles	mol/l	-0.082	0.028	3.49E-03	7.92E-03	1.3E-01	4.4E-02	3.81E-03	9.84E-03
Phospholipids in small LDL	mmol/l	-0.049	0.033	1.30E-01	1.91E-01	5.5E-02	5.1E-02	2.83E-01	3.36E-01
Phospholipids to total lipds ratio in small LDL	%	0.031	0.029	2.87E-01	3.65E-01	-9.9E-02	4.5E-02	2.80E-02	5.12E-02
Triglycerides in small LDL	mmol/l	-0.089	0.030	3.59E-03	8.08E-03	1.6E-01	4.8E-02	1.14E-03	3.66E-03
Triglycerides to total lipids ratio in small LDL	%	-0.032	0.029	2.68E-01	3.47E-01	6.6E-02	4.5E-02	1.45E-01	1.90E-01
Total cholesterol in small VLDL	mmol/l	-0.101	0.030	6.05E-04	1.75E-03	2.1E-01	4.6E-02	8.36E-06	8.55E-05
Total cholesterol to total lipids ratio in small VLDL	%	0.075	0.029	1.06E-02	2.11E-02	-4.3E-02	4.6E-02	3.54E-01	4.10E-01
Cholesterol esters in small VLDL	mmol/l	-0.077	0.029	8.34E-03	1.69E-02	1.8E-01	4.6E-02	9.43E-05	5.17E-04
Cholesterol esters to total lipids ratio in small VLDL	%	0.067	0.029	2.23E-02	3.95E-02	-3.8E-02	4.6E-02	4.09E-01	4.60E-01
Free cholesterol in small VLDL	mmol/l	-0.123	0.029	3.14E-05	1.40E-04	2.2E-01	4.6E-02	1.83E-06	2.60E-05
Free cholesterol to total lipids ratio in small VLDL	%	0.089	0.029	2.18E-03	5.15E-03	-6.4E-02	4.6E-02	1.58E-01	2.03E-01
Total lipids in small VLDL	mmol/l	-0.130	0.030	1.54E-05	8.07E-05	2.2E-01	4.7E-02	4.45E-06	5.56E-05
Concentration of small VLDL particles	mol/l	-0.134	0.028	1.26E-06	2.21E-05	2.3E-01	4.3E-02	1.11E-07	1.03E-05
Phospholipids in small VLDL	mmol/l	-0.129	0.030	1.40E-05	7.68E-05	2.2E-01	4.6E-02	2.40E-06	3.18E-05
Phospholipids to total lipds ratio in small VLDL	%	0.108	0.029	2.02E-04	6.88E-04	-1.4E-01	4.5E-02	1.85E-03	5.61E-03
Triglycerides in small VLDL	mmol/l	-0.140	0.030	2.43E-06	2.60E-05	2.1E-01	4.6E-02	7.17E-06	7.68E-05
Triglycerides to total lipids ratio in small VLDL	%	-0.092	0.029	1.70E-03	4.08E-03	7.5E-02	4.6E-02	9.95E-02	1.37E-01
Serum total cholesterol	mmol/l	-0.065	0.028	2.20E-02	3.93E-02	7.6E-02	4.4E-02	8.39E-02	1.19E-01
Serum total triglycerides	mmol/l	-0.139	0.029	2.35E-06	2.60E-05	1.8E-01	4.6E-02	8.15E-05	4.70E-04

Saturated fatty acids	mmol/l	-0.143	0.029	6.48E-07	2.08E-05	1.1E-01	4.5E-02	1.42E-02	2.80E-02
Ratio of saturated fatty acids to total fatty acids	%	0.044	0.027	1.05E-01	1.60E-01	-7.8E-02	4.3E-02	6.92E-02	1.01E-01
Sphingomyelins	mmol/l	-0.099	0.031	1.22E-03	3.22E-03	-8.3E-02	4.8E-02	8.27E-02	1.19E-01
Ratio of triglycerides to phosphoglycerides	%	-0.121	0.030	4.12E-05	1.75E-04	1.9E-01	4.6E-02	5.52E-05	3.65E-04
Total cholines	mmol/l	-0.139	0.036	1.20E-04	4.28E-04	-9.8E-02	5.6E-02	8.29E-02	1.19E-01
Total fatty acids	mmol/l	-0.145	0.028	3.58E-07	1.61E-05	1.2E-01	4.4E-02	6.17E-03	1.43E-02
Total phosphoglycerides	mmol/l	-0.154	0.035	1.39E-05	7.68E-05	-8.3E-02	5.5E-02	1.33E-01	1.76E-01
Tyrosine	mmol/l	-0.028	0.029	3.31E-01	4.12E-01	-2.1E-02	4.5E-02	6.36E-01	6.81E-01
Estimated degree of unsaturation		0.039	0.028	1.60E-01	2.30E-01	-6.4E-02	4.4E-02	1.42E-01	1.87E-01
Valine	mmol/l	-0.013	0.029	6.43E-01	7.00E-01	3.4E-02	4.5E-02	4.53E-01	5.02E-01
Total cholesterol in VLDL	mmol/l	-0.127	0.029	1.32E-05	7.68E-05	1.9E-01	4.6E-02	2.24E-05	1.94E-04
Mean diameter for VLDL particles	nm	-0.122	0.030	4.80E-05	1.93E-04	1.2E-01	4.7E-02	8.68E-03	1.86E-02
Triglycerides in VLDL	mmol/l	-0.142	0.029	1.47E-06	2.21E-05	1.8E-01	4.6E-02	7.91E-05	4.70E-04
Total cholesterol in very large HDL	mmol/l	0.054	0.029	6.58E-02	1.07E-01	-9.3E-02	4.6E-02	4.42E-02	7.32E-02
Total cholesterol to total lipids ratio in very large HDL	%	-0.098	0.028	4.33E-04	1.32E-03	7.6E-02	4.4E-02	8.38E-02	1.19E-01
Cholesterol esters in very large HDL	mmol/l	0.045	0.029	1.27E-01	1.88E-01	-8.4E-02	4.6E-02	6.78E-02	1.01E-01
Cholesterol esters to total lipids ratio in very large HDL	%	-0.109	0.028	1.08E-04	3.98E-04	1.1E-01	4.4E-02	1.60E-02	3.10E-02
Free cholesterol in very large HDL	mmol/l	0.070	0.029	1.69E-02	3.15E-02	-1.3E-01	4.6E-02	4.34E-03	1.09E-02
Free cholesterol to total lipids ratio in very large HDL	%	0.006	0.028	8.33E-01	8.63E-01	-1.3E-01	4.3E-02	2.99E-03	8.02E-03
Total lipids in very large HDL	mmol/l	0.079	0.030	7.95E-03	1.64E-02	-1.0E-01	4.7E-02	2.89E-02	5.21E-02
Concentration of very large HDL particles	mol/l	0.068	0.029	2.07E-02	3.79E-02	-1.2E-01	4.6E-02	8.06E-03	1.76E-02
Phospholipids in very large HDL	mmol/l	0.103	0.030	5.26E-04	1.54E-03	-1.2E-01	4.7E-02	7.73E-03	1.71E-02
Phospholipids to total lipds ratio in very large HDL	%	0.116	0.028	4.38E-05	1.79E-04	-9.6E-02	4.5E-02	3.10E-02	5.50E-02
Triglycerides in very large HDL	mmol/l	-0.053	0.028	6.45E-02	1.06E-01	4.8E-02	4.5E-02	2.80E-01	3.36E-01
Triglycerides to total lipids ratio in very large HDL	%	-0.111	0.030	1.69E-04	5.87E-04	1.3E-01	4.6E-02	7.13E-03	1.59E-02
Total cholesterol in very large VLDL	mmol/l	-0.126	0.030	2.06E-05	1.01E-04	1.6E-01	4.6E-02	8.48E-04	3.03E-03
Total cholesterol to total lipids ratio in very large VLDL	%	0.029	0.028	3.02E-01	3.82E-01	7.7E-02	4.4E-02	7.99E-02	1.16E-01
Cholesterol esters in very large VLDL	mmol/l	-0.125	0.030	2.19E-05	1.05E-04	1.7E-01	4.6E-02	3.46E-04	1.53E-03
Cholesterol esters to total lipids ratio in very large VLDL	%	0.083	0.029	3.80E-03	8.46E-03	1.8E-02	4.5E-02	6.89E-01	7.25E-01
Free cholesterol in very large VLDL	mmol/l	-0.127	0.029	1.34E-05	7.68E-05	1.4E-01	4.6E-02	1.90E-03	5.62E-03
Free cholesterol to total lipids ratio in very large VLDL	%	-0.093	0.034	5.67E-03	1.24E-02	9.9E-02	5.2E-02	5.90E-02	9.15E-02
Total lipids in very large VLDL	mmol/l	-0.130	0.030	1.66E-05	8.51E-05	1.4E-01	4.7E-02	2.54E-03	7.05E-03
Concentration of very large VLDL particles	mol/l	-0.133	0.029	5.66E-06	4.36E-05	1.4E-01	4.6E-02	2.39E-03	6.78E-03
Phospholipids in very large VLDL	mmol/l	-0.133	0.029	6.04E-06	4.38E-05	1.5E-01	4.6E-02	1.34E-03	4.25E-03

Phospholipids to total lipds ratio in very large VLDL	%	-0.114	0.032	3.44E-04	1.09E-03	1.1E-01	5.0E-02	2.92E-02	5.21E-02
Triglycerides in very large VLDL	mmol/l	-0.132	0.030	1.16E-05	7.22E-05	1.4E-01	4.7E-02	2.41E-03	6.78E-03
Triglycerides to total lipids ratio in very large VLDL	%	0.043	0.031	1.61E-01	2.30E-01	-1.2E-01	4.7E-02	1.02E-02	2.11E-02
Total cholesterol in very small VLDL	mmol/l	-0.011	0.029	6.98E-01	7.48E-01	1.0E-01	4.6E-02	2.17E-02	4.04E-02
Total cholesterol to total lipids ratio in very small VLDL	%	0.100	0.028	4.20E-04	1.29E-03	-1.5E-01	4.4E-02	7.00E-04	2.62E-03
Cholesterol esters in very small VLDL	mmol/l	-0.010	0.029	7.13E-01	7.57E-01	9.1E-02	4.5E-02	4.15E-02	7.06E-02
Cholesterol esters to total lipids ratio in very small VLDL	%	0.088	0.027	1.25E-03	3.24E-03	-1.6E-01	4.3E-02	2.59E-04	1.21E-03
Free cholesterol in very small VLDL	mmol/l	-0.021	0.030	4.91E-01	5.58E-01	1.1E-01	4.7E-02	1.61E-02	3.10E-02
Free cholesterol to total lipids ratio in very small VLDL	%	0.044	0.035	2.10E-01	2.86E-01	-5.8E-02	5.5E-02	2.96E-01	3.48E-01
Total lipids in very small VLDL	mmol/l	-0.039	0.029	1.78E-01	2.46E-01	1.6E-01	4.6E-02	4.29E-04	1.72E-03
Concentration of very small VLDL particles	mol/l	-0.067	0.028	1.61E-02	3.05E-02	1.8E-01	4.4E-02	5.71E-05	3.67E-04
Phospholipids in very small VLDL	mmol/l	-0.044	0.030	1.38E-01	2.02E-01	1.3E-01	4.6E-02	6.69E-03	1.52E-02
Phospholipids to total lipds ratio in very small VLDL	%	-0.014	0.032	6.66E-01	7.17E-01	-1.3E-02	4.9E-02	7.86E-01	8.18E-01
Triglycerides in very small VLDL	mmol/l	-0.094	0.029	1.27E-03	3.24E-03	2.2E-01	4.6E-02	1.61E-06	2.60E-05
Triglycerides to total lipids ratio in very small VLDL	%	-0.070	0.029	1.66E-02	3.12E-02	1.1E-01	4.6E-02	1.40E-02	2.78E-02
Total cholesterol in chylomicrons and extremely large VLDL	mmol/l	-0.127	0.029	1.39E-05	7.68E-05	1.5E-01	4.6E-02	9.86E-04	3.28E-03
Total cholesterol to total lipids ratio in chylomicrons and extremely large VLDL	%	-0.116	0.030	1.02E-04	3.82E-04	1.3E-01	4.7E-02	5.20E-03	1.25E-02
Cholesterol esters in chylomicrons and extremely large VLDL	mmol/l	-0.128	0.029	9.32E-06	6.20E-05	1.7E-01	4.5E-02	2.36E-04	1.15E-03
Cholesterol esters to total lipids ratio in chylomicrons and extremely large VLDL	%	-0.080	0.030	7.31E-03	1.55E-02	6.2E-02	4.7E-02	1.83E-01	2.31E-01
Free cholesterol in chylomicrons and extremely large VLDL	mmol/l	-0.121	0.029	3.16E-05	1.40E-04	1.3E-01	4.6E-02	5.15E-03	1.25E-02
Free cholesterol to total lipids ratio in chylomicrons and extremely large VLDL	%	-0.142	0.032	9.37E-06	6.20E-05	1.2E-01	5.0E-02	1.36E-02	2.73E-02
Total lipids in chylomicrons and extremely large VLDL	mmol/l	-0.122	0.030	4.25E-05	1.77E-04	1.3E-01	4.7E-02	5.22E-03	1.25E-02
Concentration of chylomicrons and extremely large VLDL particles	mol/l	-0.133	0.029	5.81E-06	4.36E-05	1.3E-01	4.6E-02	6.53E-03	1.50E-02
Phospholipids in chylomicrons and extremely large VLDL	mmol/l	-0.130	0.029	7.47E-06	5.25E-05	1.3E-01	4.6E-02	5.03E-03	1.24E-02
Phospholipids to total lipds ratio in chylomicrons and extremely large VLDL	%	-0.178	0.033	5.01E-08	9.48E-06	8.5E-02	5.1E-02	9.83E-02	1.37E-01
Triglycerides in chylomicrons and extremely large VLDL	mmol/l	-0.124	0.030	2.95E-05	1.38E-04	1.3E-01	4.7E-02	6.14E-03	1.43E-02
Triglycerides to total lipids ratio in chylomicrons and extremely large VLDL	%	0.056	0.022	1.12E-02	2.21E-02	-8.6E-02	3.5E-02	1.31E-02	2.66E-02

Abbreviations: FDR, false-discovery rate; SE, standard error.
Models were adjusted for age, sex, fasting time, region, smoking status, education, income, self-rated health, intake of fruit and meat, sedentary leisure time (for total physical activity), and total physical activity (for sedentary leisure time).
The SD was 14 MET-h/day for physical activity and 1.5 h/day for sedentary leisure time.
The regression dilution ratio was 0.52 for physical activity and 0.34 for sedentary leisure time.

Supplemental Figure 1. Associations of total physical activity with 8 selected clinical chemistry biomarkers (n=18,175)

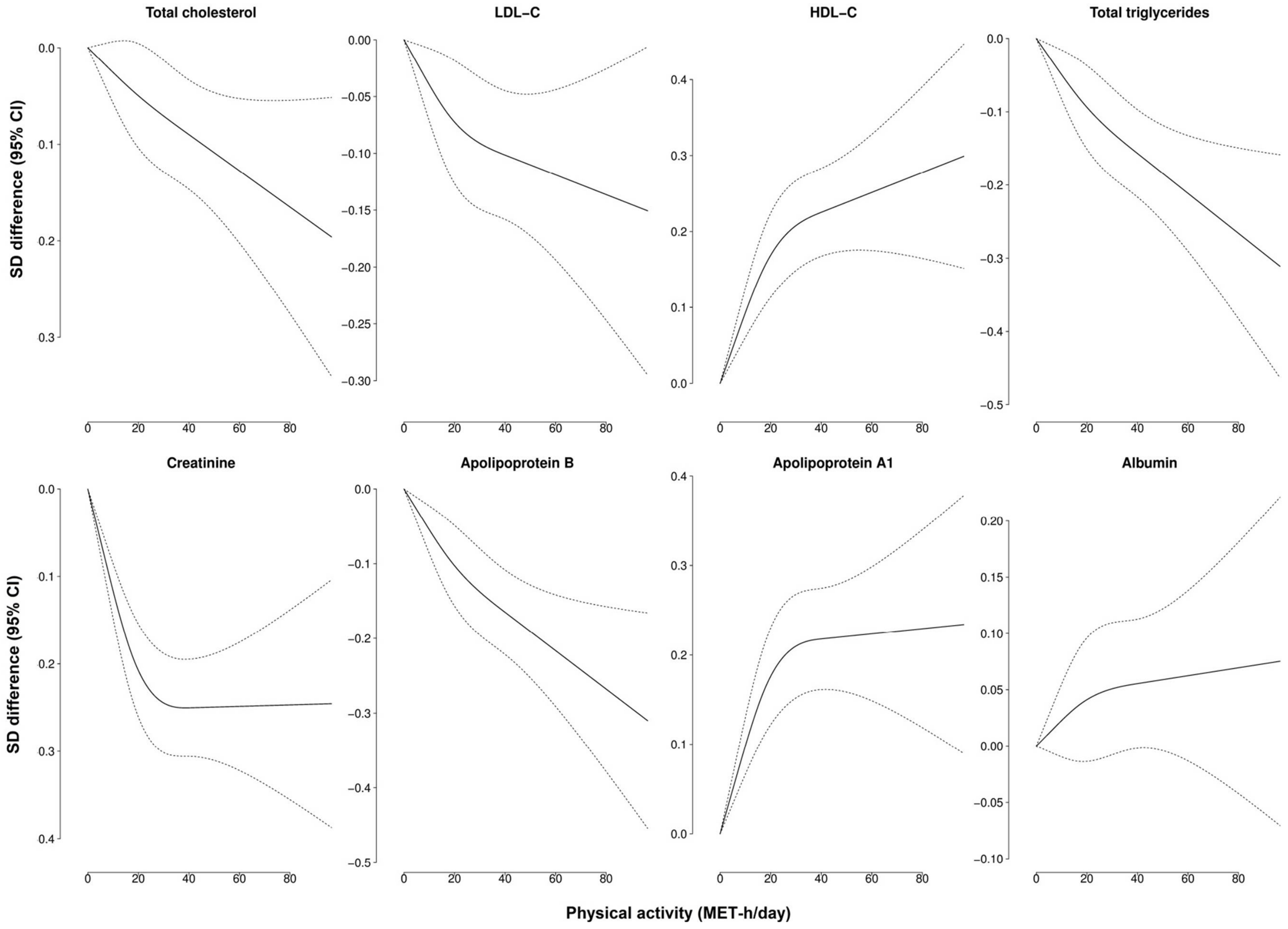

Supplemental Figure 2. Associations of total physical activity with selected NMR-measured metabolomics markers (n=4660)

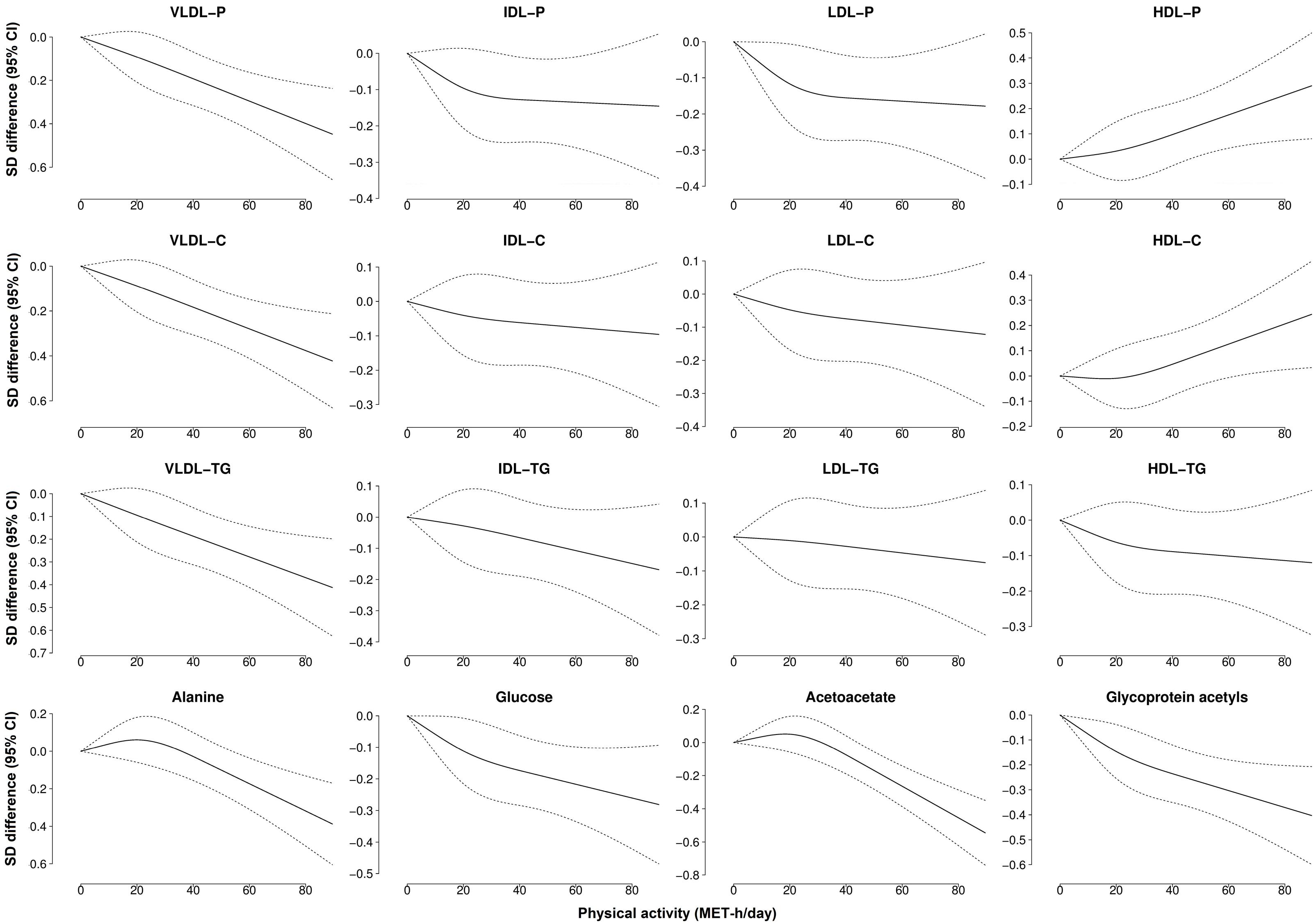

Supplemental Figure 3. Adjusted SD differences (95% CI) of 8 biomarkers, as measured by both clinical chemistry and NMR metabolomics, associated with 4 units higher usual total physical activity (n=4660)*

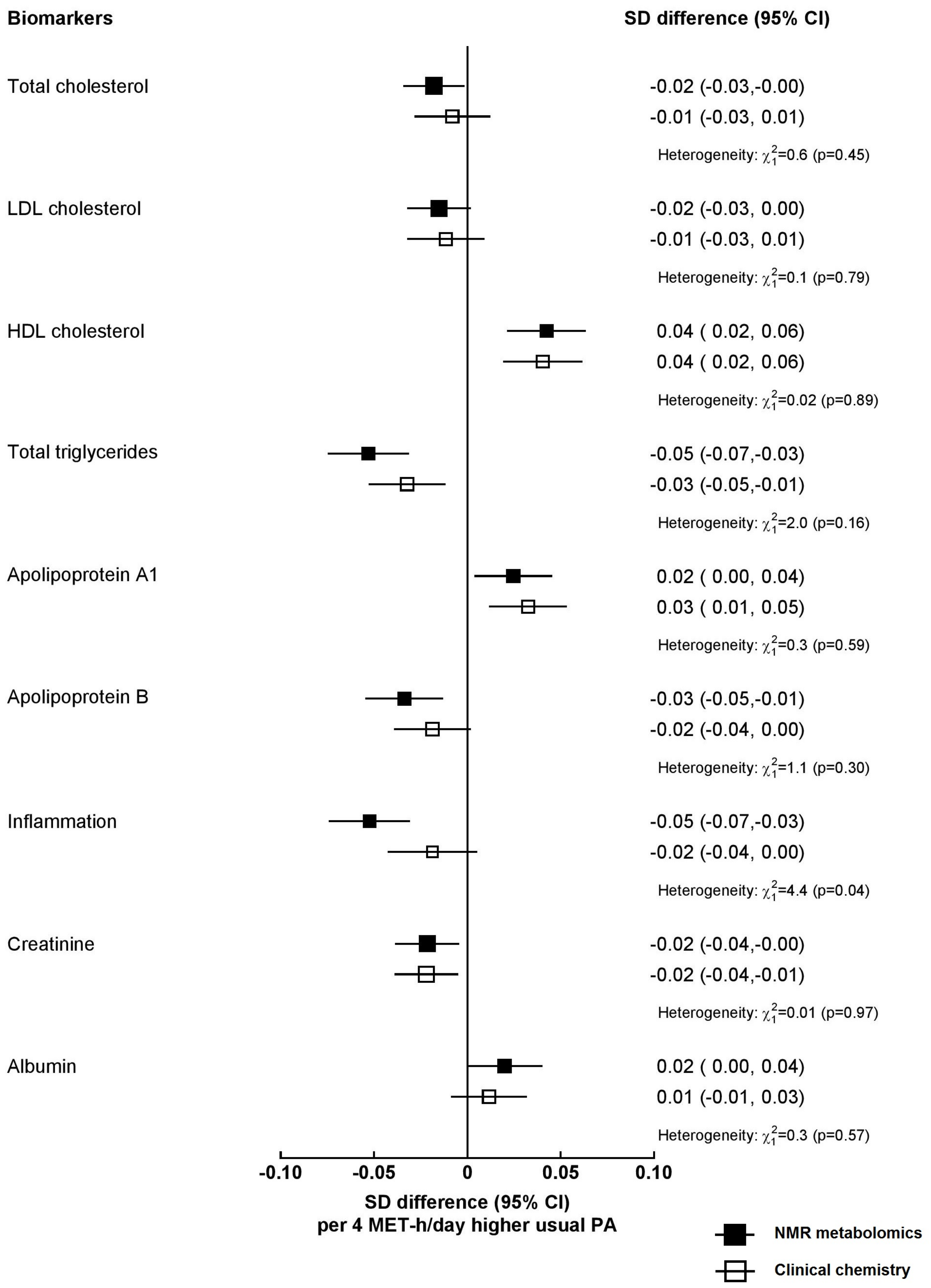

* Inflammation: NMR: glycoprotein acetyls; clinical chemistry: high sensitivity CRP.

Supplemental Figure 4. Adjusted SD differences (95% CI) of 17 clinical chemistry biomarkers associated with 4 units higher usual total physical activity (n=18,175)*

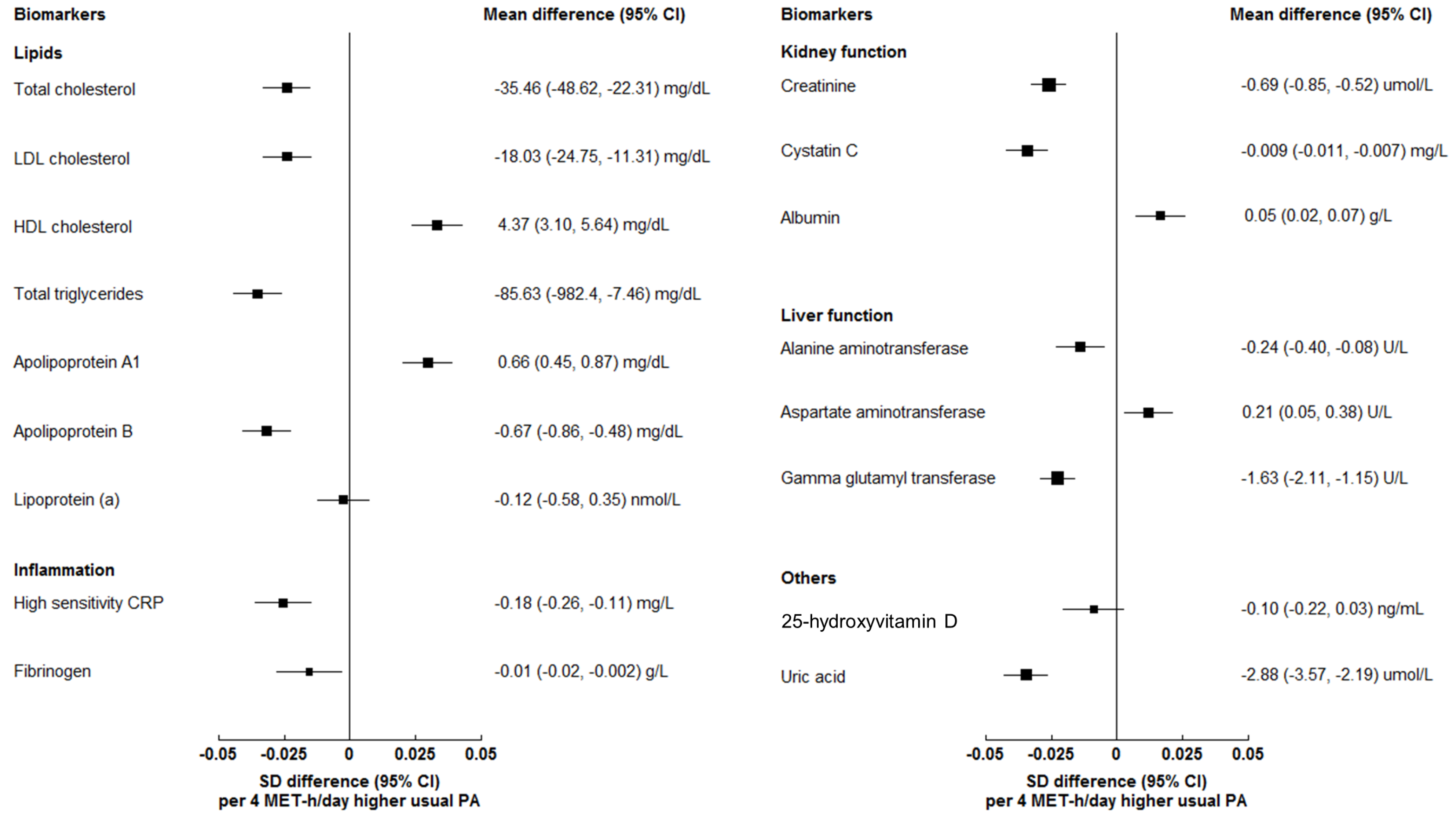

* All 17 biomarkers as measured by clinical chemistry were on the original scale to show mean differences in absolute units.

Supplemental Figure 5. Adjusted SD differences (95% CI) of 17 clinical chemistry biomarkers associated with 1-SD higher usual levels of (a) total physical activity and (b) sedentary leisure time (n=18,175)

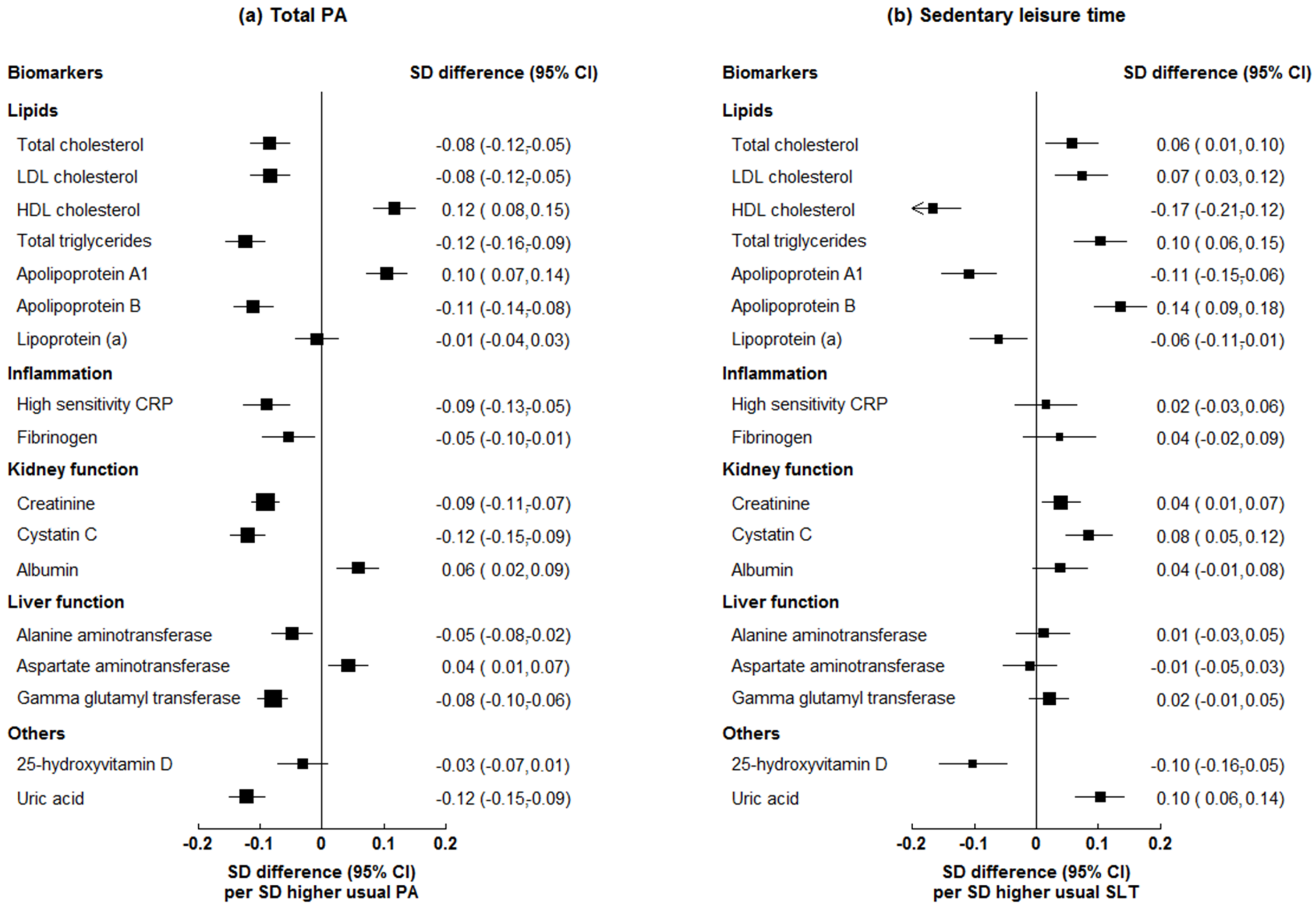

Supplemental Table 4. Proportion of the association of physical activity (PA) and sedentary leisure time (SLT) with CVD explained by selected metabolic markers*

	Total physical activity		Sedentary leisure time	
	**OR (95% CI)**	**% explained**	**OR (95% CI)**	**% explained**
Basic model*	0.86 (0.75, 0.99)	--	1.28 (1.02, 1.60)	--
+ VLDL lipoprotein particles	0.88 (0.77, 1.02)	13.5	1.26 (1.01, 1.58)	5.6
+ VLDL cholesterol	0.88 (0.77, 1.02)	13.4	1.26 (1.01, 1.58)	5.6
+ VLDL triglycerides	0.88 (0.76, 1.02)	13.3	1.27 (1.01, 1.58)	5.1
+ LDL lipoprotein particles	0.87 (0.76, 1.01)	4.0	1.26 (1.01, 1.58)	6.2
+ LDL cholesterol	0.87 (0.75, 1.004)	10.1	1.27 (1.01, 1.58)	5.3
+ LDL triglycerides	0.87 (0.76, 1.01)	4.6	1.27 (1.01, 1.59)	4.7
+ HDL lipoprotein particles	0.87 (0.76, 1.01)	4.8	1.26 (1.01, 1.58)	5.6
+ HDL cholesterol	0.87 (0.75, 1.004)	2.5	1.28 (1.02, 1.60)	3.4
+ HDL triglycerides	0.87 (0.75, 1.004)	2.9	1.28 (1.03, 1.61)	0.4
+ Branch chained amino acids	0.87 (0.75, 1.002)	1.0	1.28 (1.02, 1.60)	0
+ Aromatic amino acids	0.87 (0.78, 1.002)	1.1	1.28 (1.02, 1.60)	1.4
+ Other amino acids	0.87 (0.75, 1.01)	3.9	1.28 (1.02, 1.60)	1.1
+ Glycoprotein acetyls	0.90 (0.78, 1.04)	25.0	1.25 (0.996, 1.56)	11.0
+ 18 principal components†	0.96 (0.82, 1.12)	69.9	1.11 (0.87, 1.41)	50.0

* Basic model included age, sex, fasting time, region, smoking, education, income, self-rated health, and intake of fresh fruit and red meat, sedentary leisure time (for physical activity), and physical activity (for sedentary leisure time).
† In the complete case analysis (n=4151), the adjusted ORs were 0.90 (0.78-1.03) per 14 units higher PA and were 1.22 (0.97-1.55) per 1.5 units higher SLT.

Supplemental Figure 6. Associations of usual occupational and non-occupational physical activity with lipoprotein particle concentration, cholesterol and triglycerides as measured by NMR metabolomics

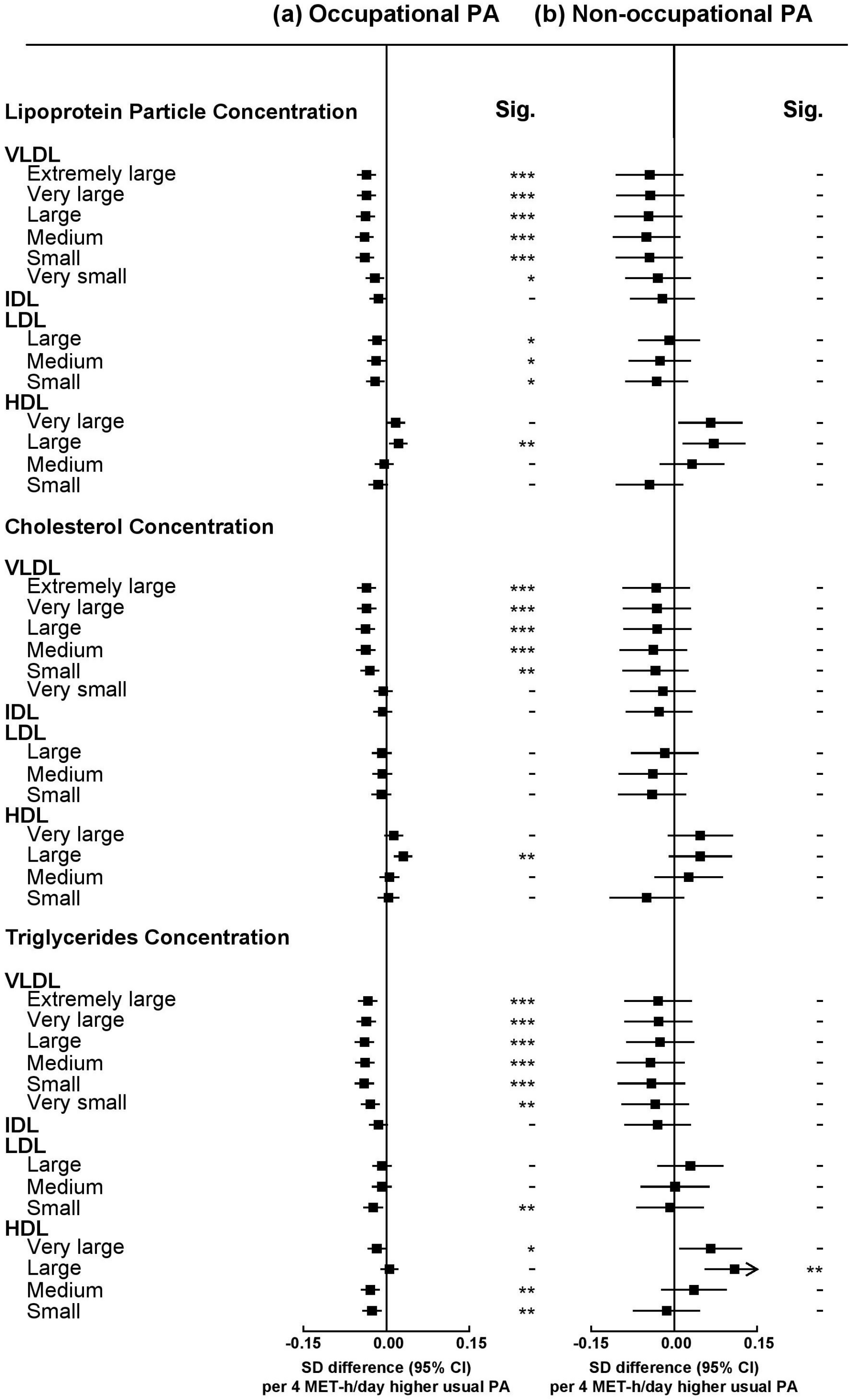

Significance: '***' < 0.0001, '**' < 0.01, '*' < 0.05, '-' > 0.05

P-values for heterogeneity comparing occupational vs non-occupational physical activity (FDR-corrected): all>0.05 except for triglycerides in large HDL.

Supplemental Figure 7. Associations of usual occupational and non-occupational physical activity with mean particle diameter, cholesterol and triglycerides as measured by NMR metabolomics

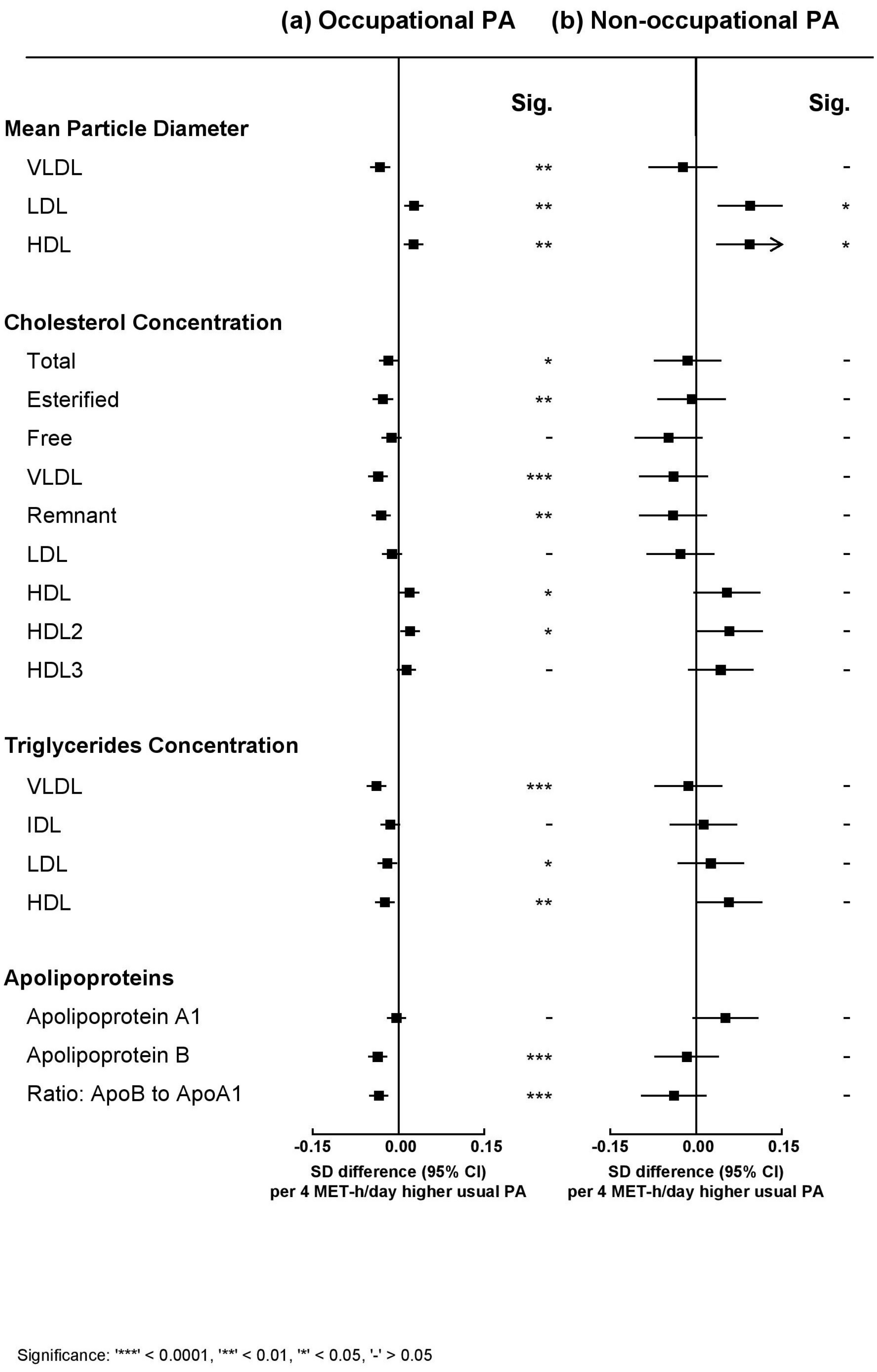

P-values for heterogeneity comparing occupational vs non-occupational physical activity (FDR-corrected): all>0.05 except for triglycerides in large HDL.

Supplemental Figure 8. Association of usual occupational and non-occupational physical activity with other metabolic traits as measured by NMR metabolomics

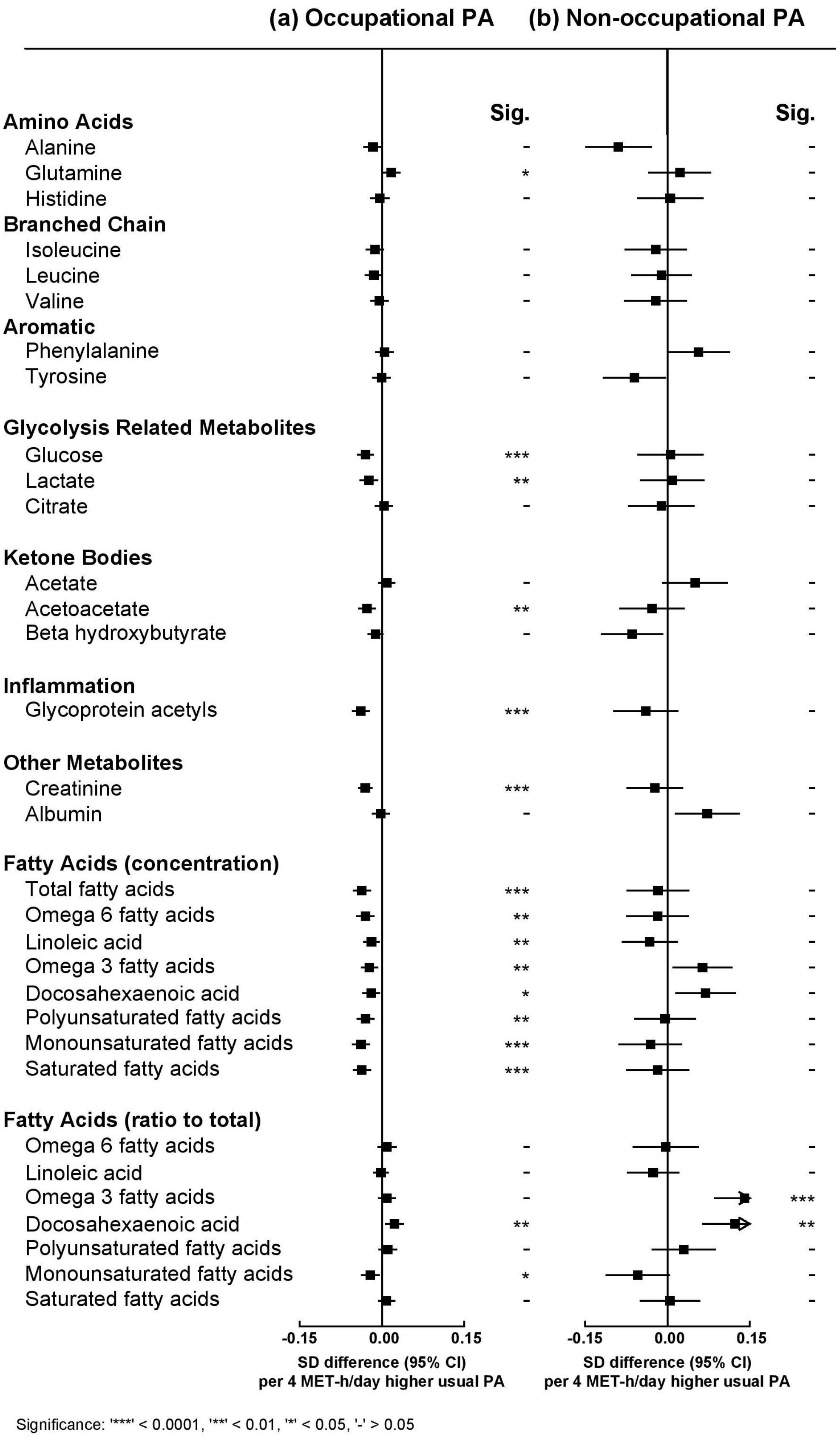

P-values for heterogeneity comparing occupational vs non-occupational physical activity (FDR-corrected): all>0.05 except for triglycerides in large HDL.

Supplemental Figure 9. Associations of usual total physical activity (PA) and sedentary leisure time (SLT) with lipoprotein particle concentration, cholesterol and triglycerides in all participants and controls, separately

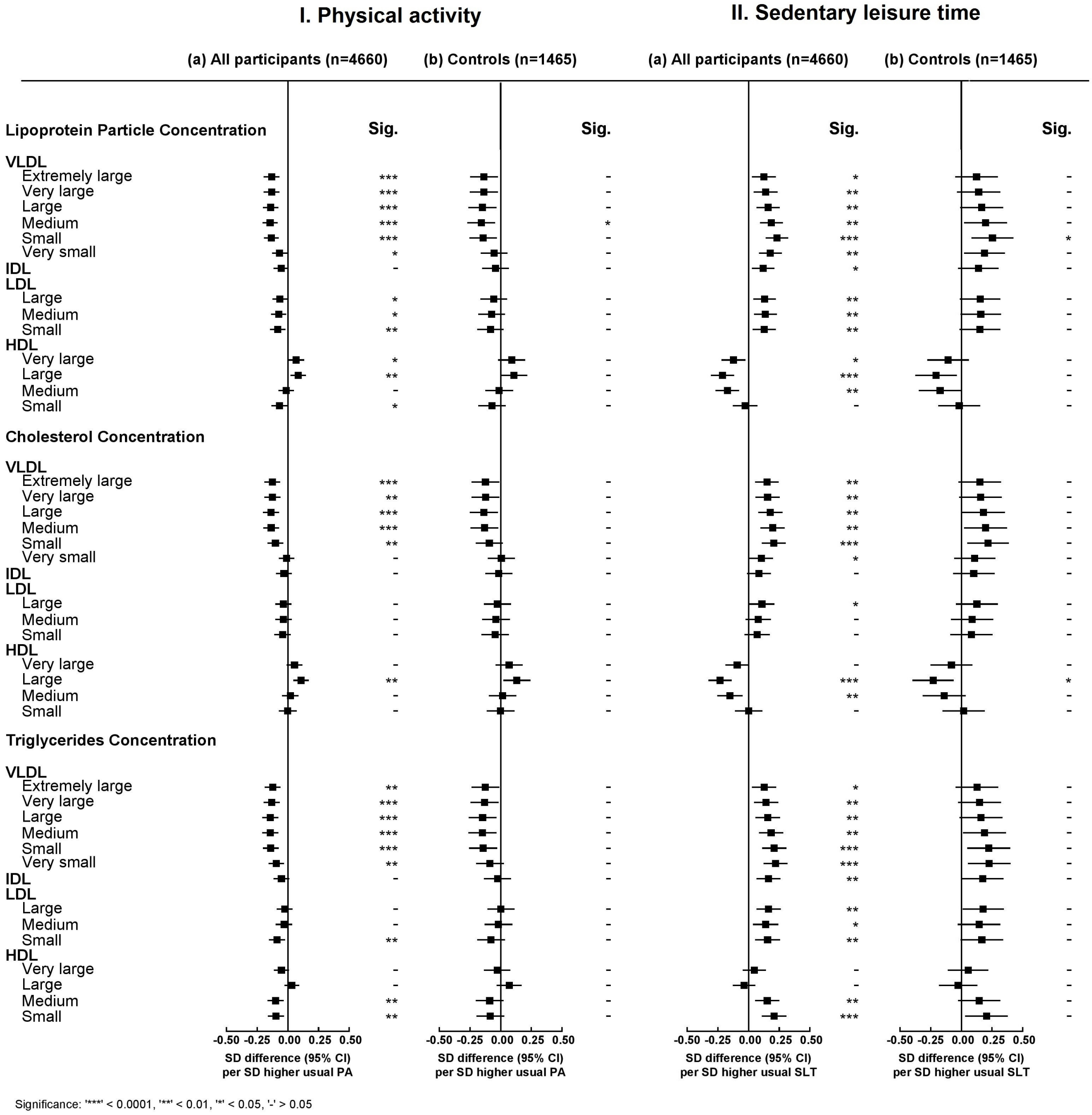

Supplemental Figure 10. Associations of usual total physical activity and sedentary leisure time with mean particle diameter, cholesterol and triglycerides in all participants and controls, separately

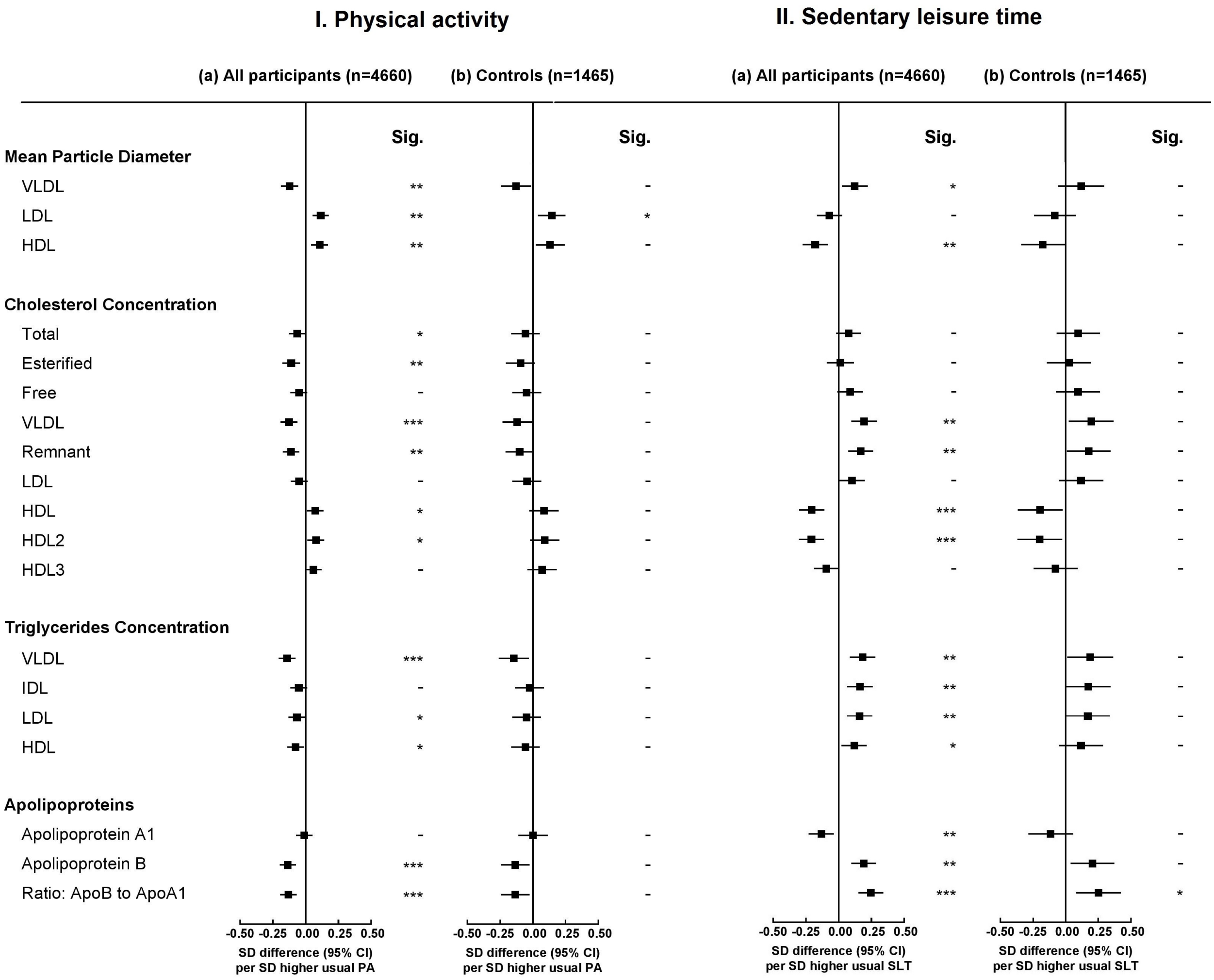

Significance: '***' < 0.0001, '**' < 0.01, '*' < 0.05, '-' > 0.05

Supplemental Figure 11. Associations of usual total physical activity and sedentary leisure time with other metabolic traits in all participants and controls, separately

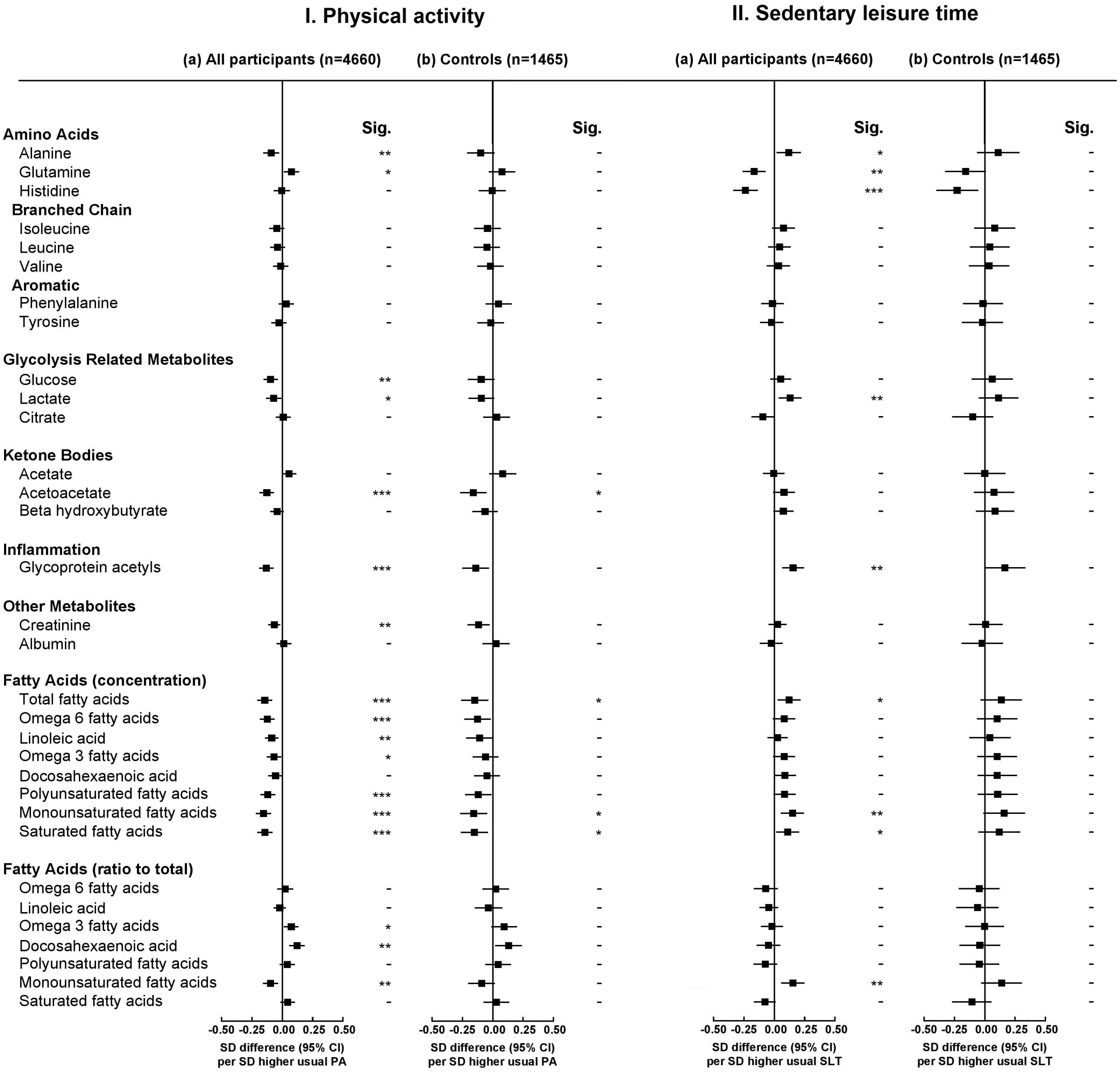

Significance: '***' < 0.0001, '**' < 0.01, '*' < 0.05, '-' > 0.05

Supplemental Figure 12. Associations of total physical activity (PA) with lipoprotein particle concentration, cholesterol and triglycerides with additional adjustments

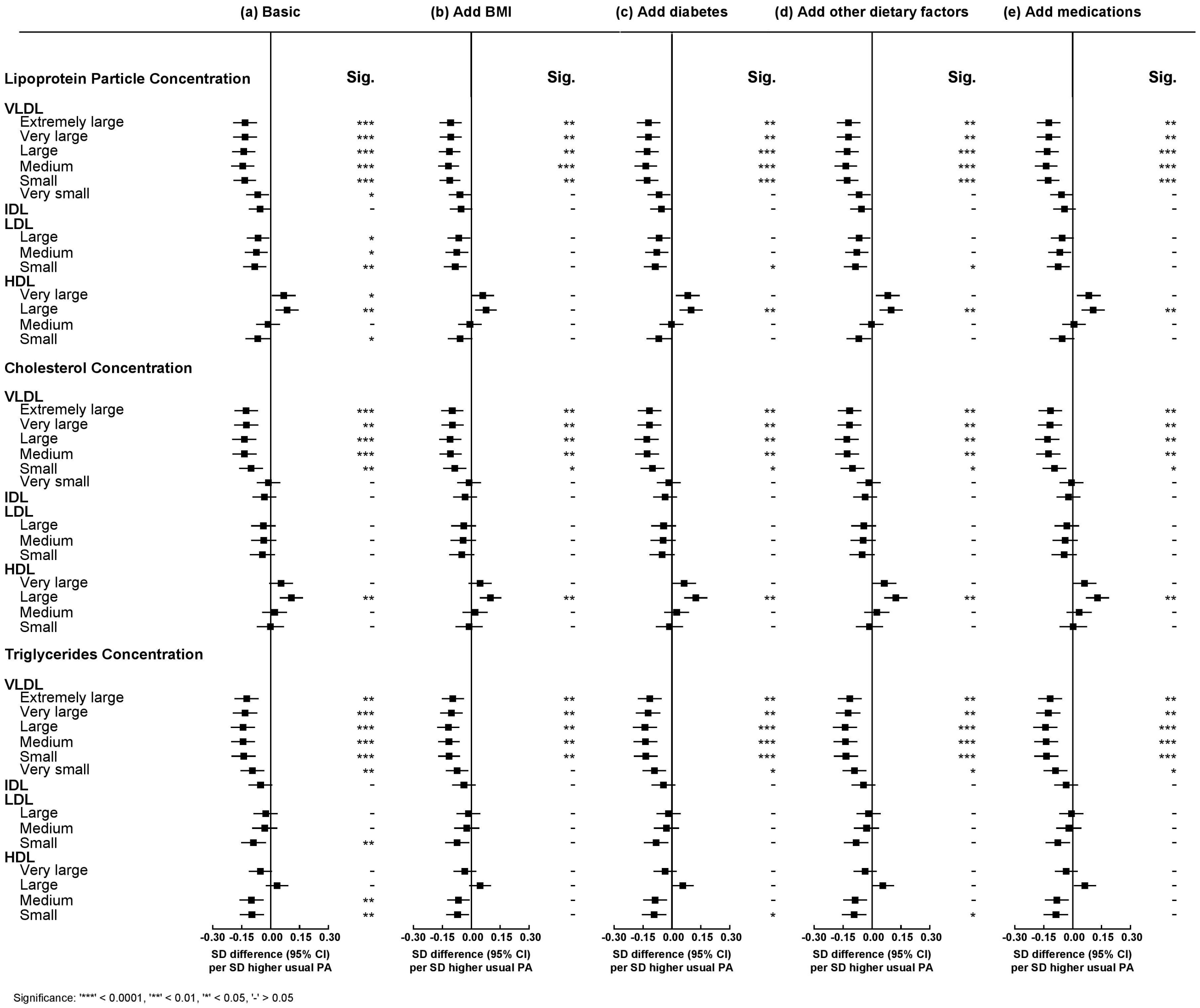

Other dietary factors include rice, wheat, staple food other than rice and wheat (e.g. corn, millet), poultry, fish/seafood, dairy, eggs, soybean, and preserved vegetables. Medications include aspirin, ACE inhibitor, beta-blocker, diuretics, calcium channel blocker, chlorpropamide or metformin, and insulin.

Supplemental Figure 13. Associations of total physical activity with mean particle diameter, cholesterol and triglycerides with additional adjustments

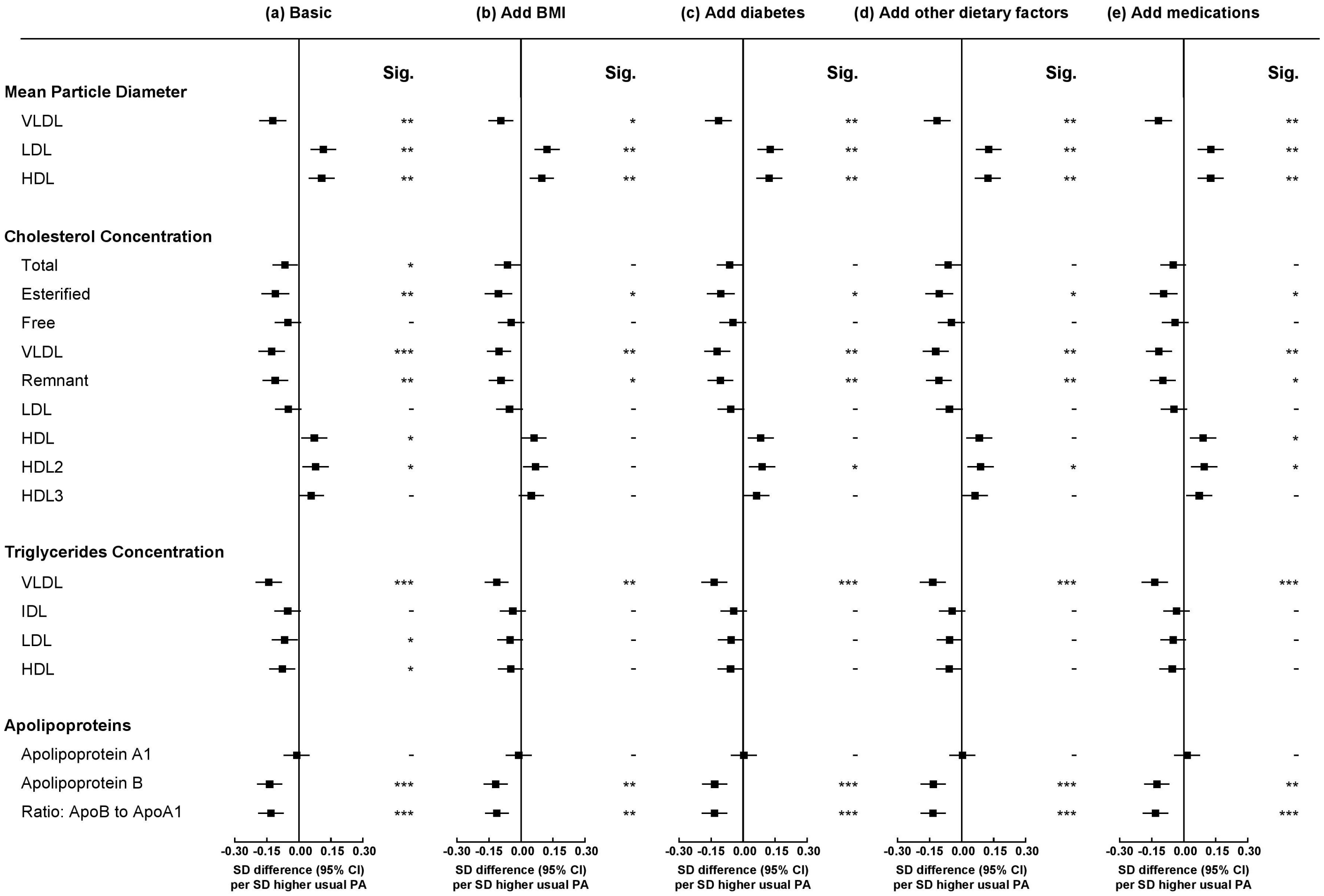

Significance: '***' < 0.0001, '**' < 0.01, '*' < 0.05, '-' > 0.05

Supplemental Figure 14. Associations of total physical activity with other metabolic traits with additional adjustments

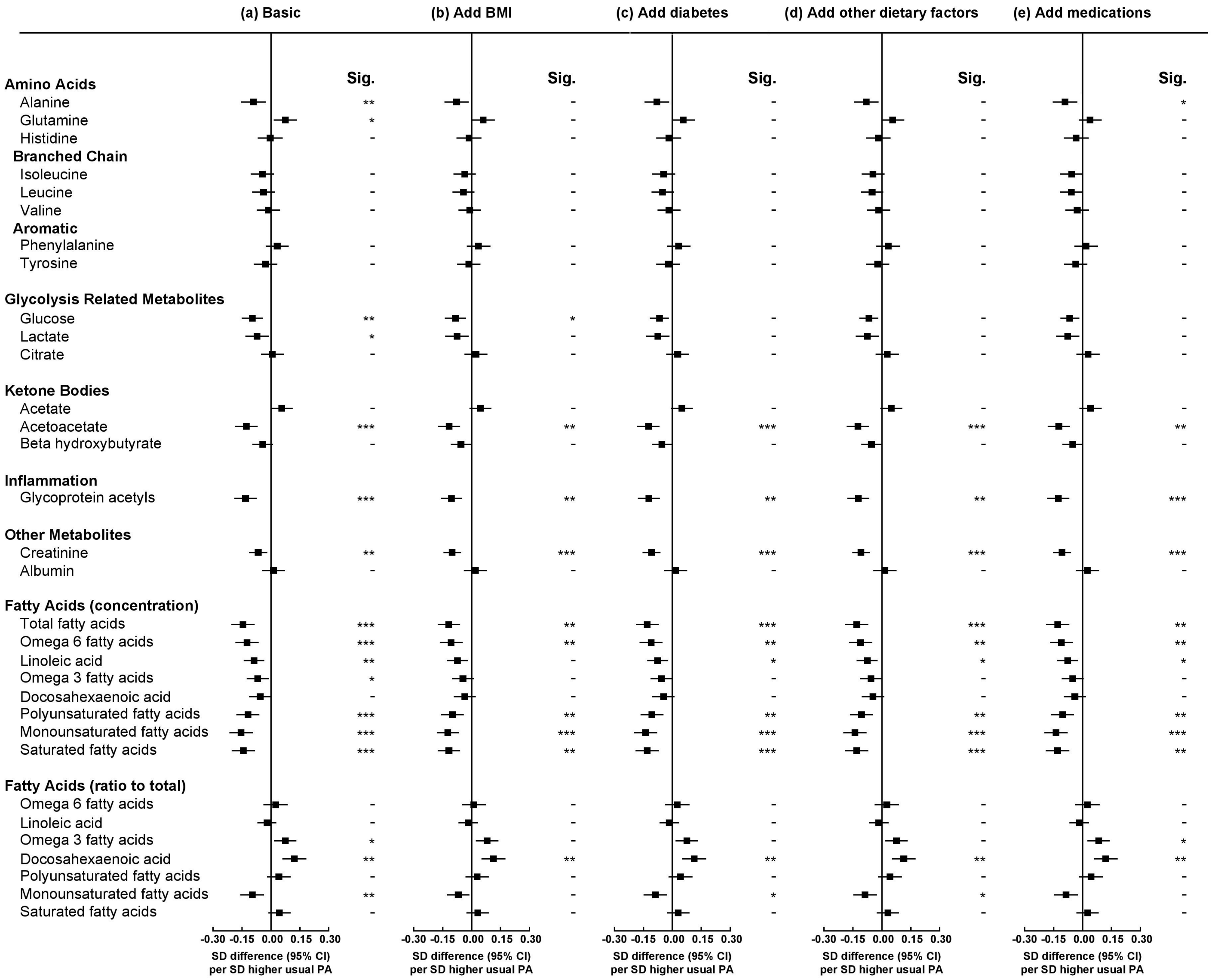

Significance: '***' < 0.0001, '**' < 0.01, '*' < 0.05, '-' > 0.05

Supplemental Figure 15. Associations of sedentary leisure time (SLT) with lipoprotein particle concentration, cholesterol and triglycerides with additional adjustments

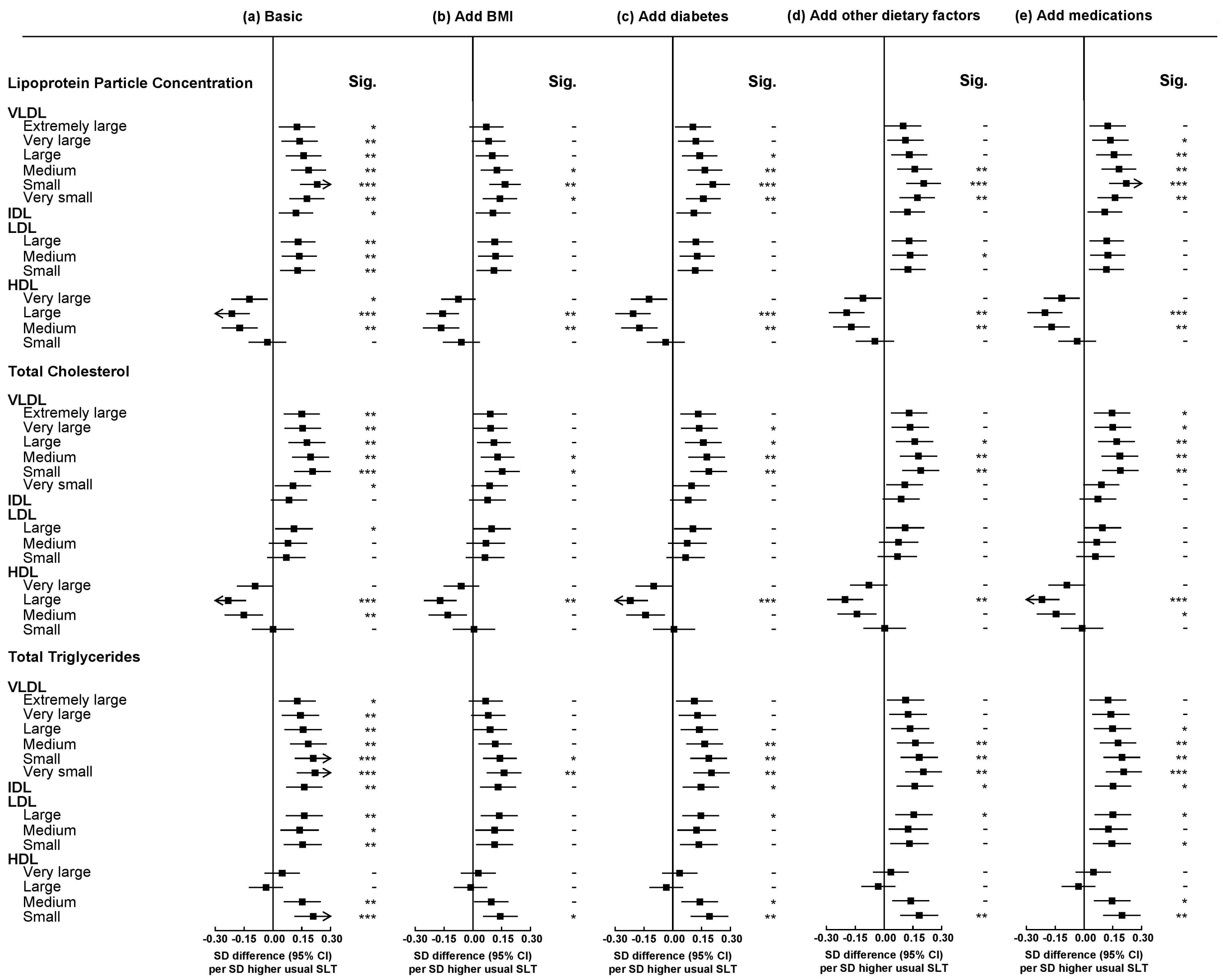

Significance: '***' < 0.0001, '**' < 0.01, '*' < 0.05, '-' > 0.05

Supplemental Figure 16. Associations of sedentary leisure time with mean particle diameter, cholesterol and triglycerides with additional adjustments

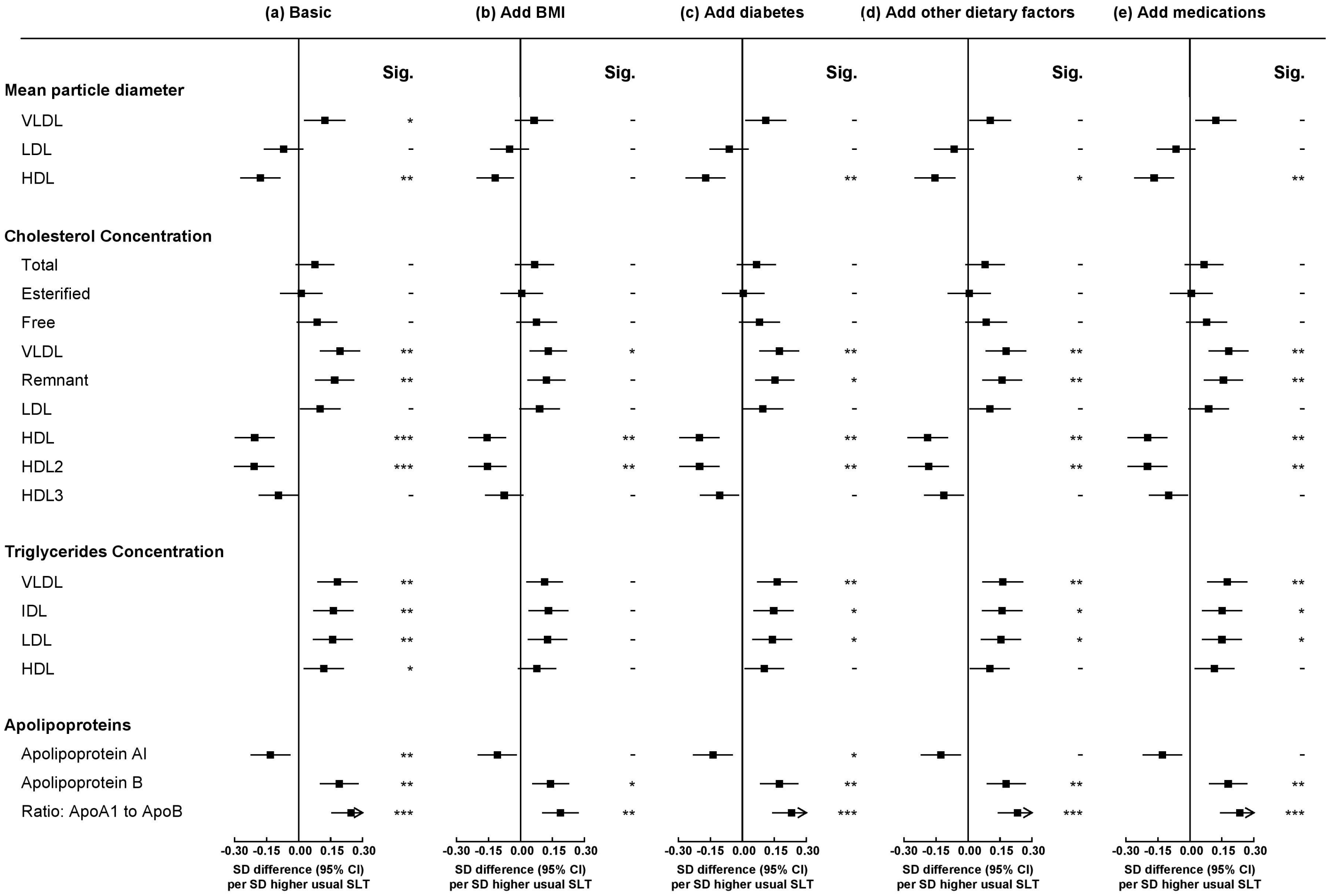

Significance: '***' < 0.0001, '**' < 0.01, '*' < 0.05, '-' > 0.05

Supplemental Figure 17. Associations of sedentary leisure time with other metabolic traits with additional adjustments

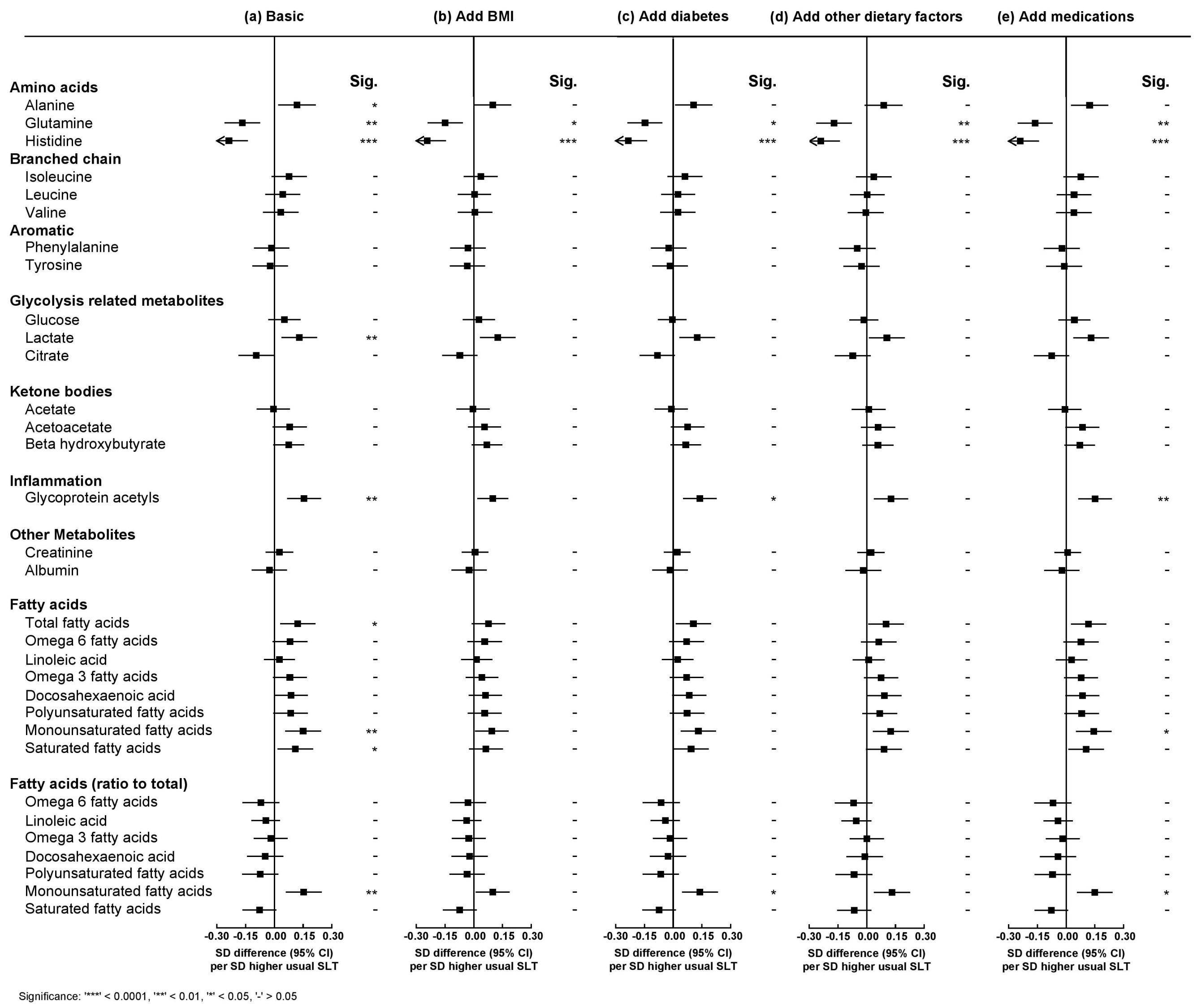

Supplemental Figure 18. Associations of total physical activity (PA) and sedentary leisure time (SLT) with lipoprotein particle concentration, cholesterol and triglycerides excluding chronic diseases and poor self-rated health at baseline

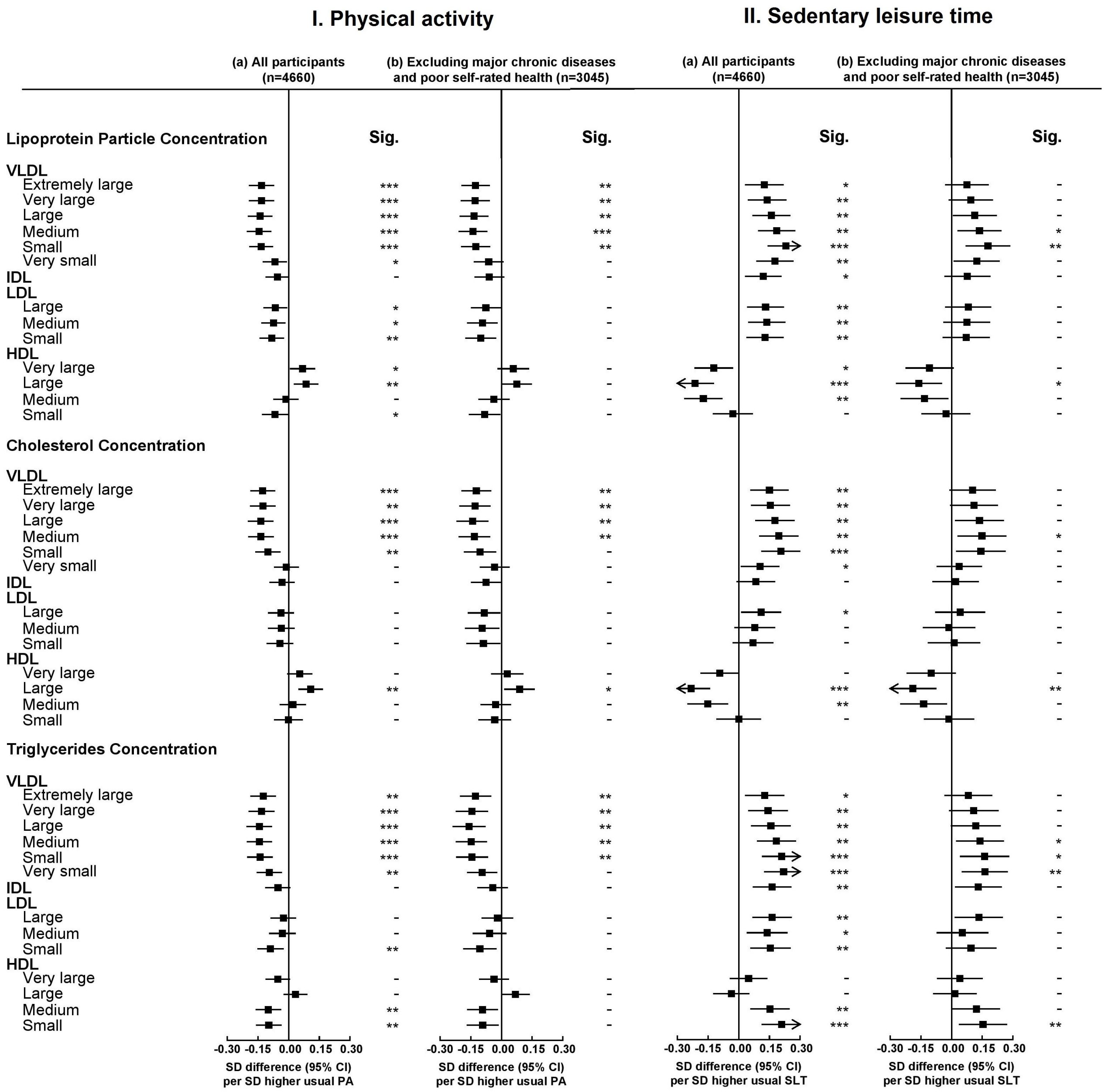

Significance: '***' < 0.0001, '**' < 0.01, '*' < 0.05, '-' > 0.05

Supplemental Figure 19. Associations of total physical activity and sedentary leisure time with mean particle diameter, cholesterol and triglycerides excluding chronic diseases and poor self-rated health at baseline

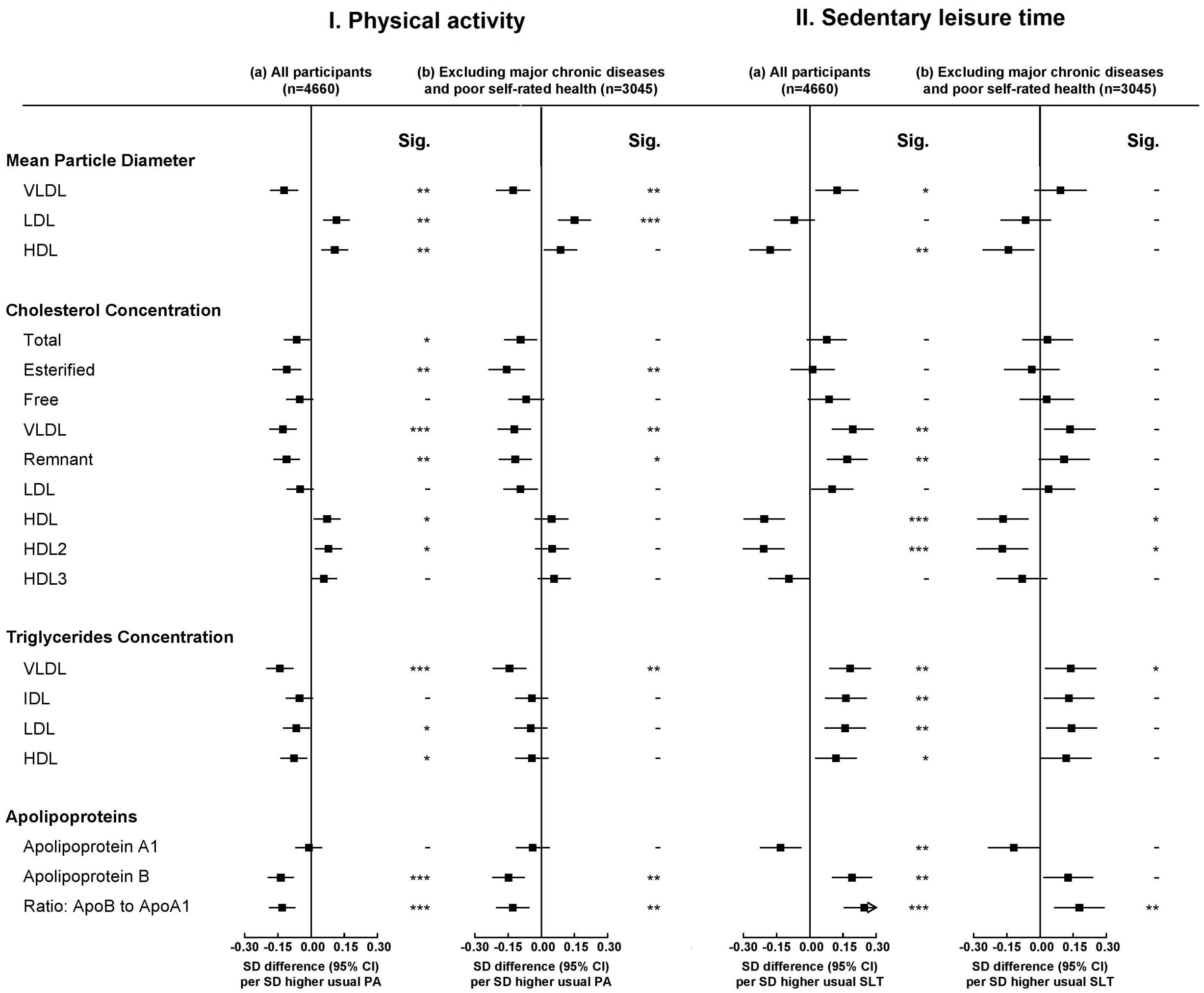

Significance: '***' < 0.0001, '**' < 0.01, '*' < 0.05, '-' > 0.05

Supplemental Figure 20. Associations of total physical activity and sedentary leisure time with other metabolic traits excluding chronic diseases and poor self-rated health at baseline

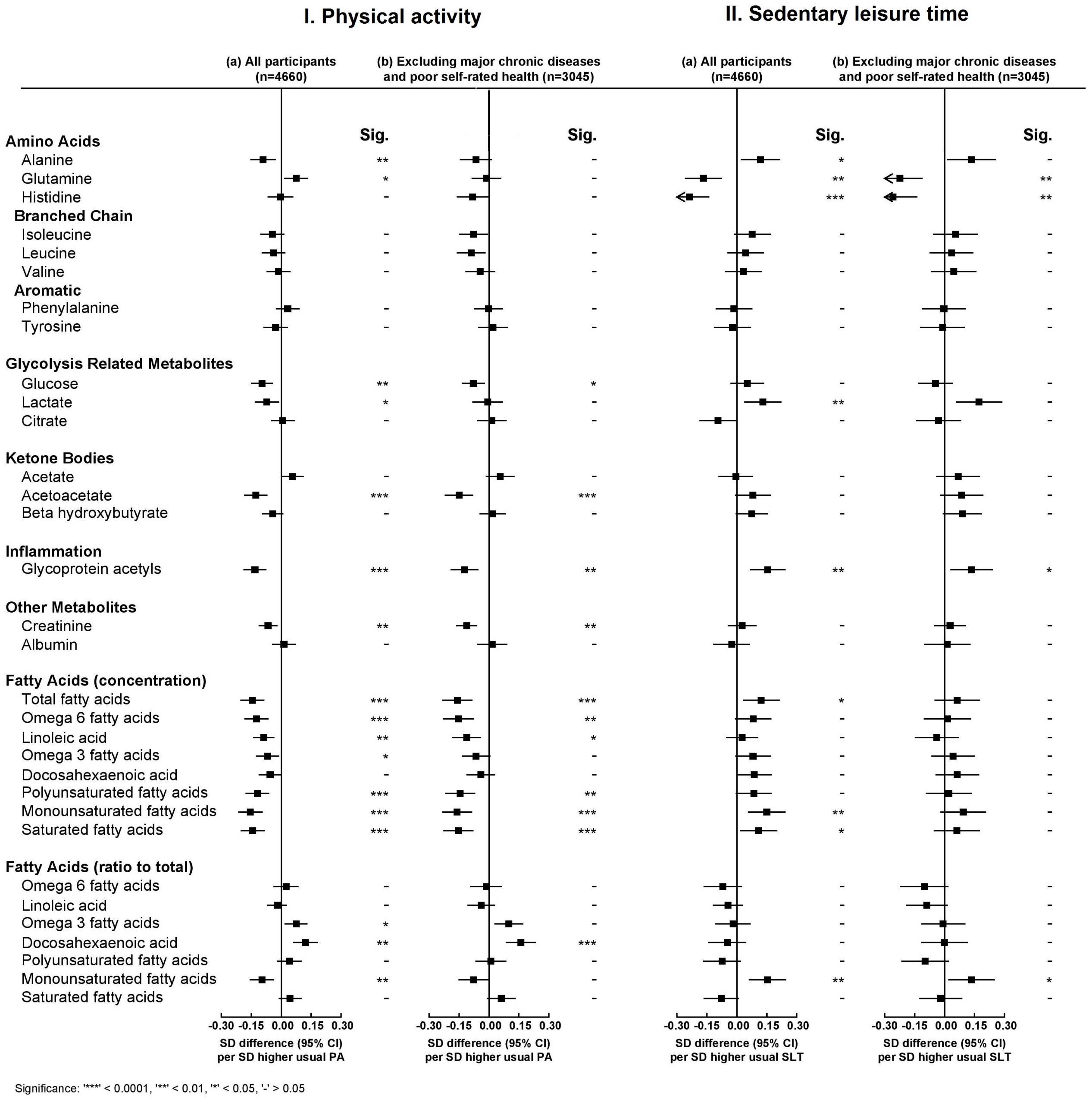

Supplemental Figure 21. Associations of total physical activity (PA) and sedentary leisure time (SLT) with lipoprotein particle concentration, cholesterol and triglycerides by fasting status

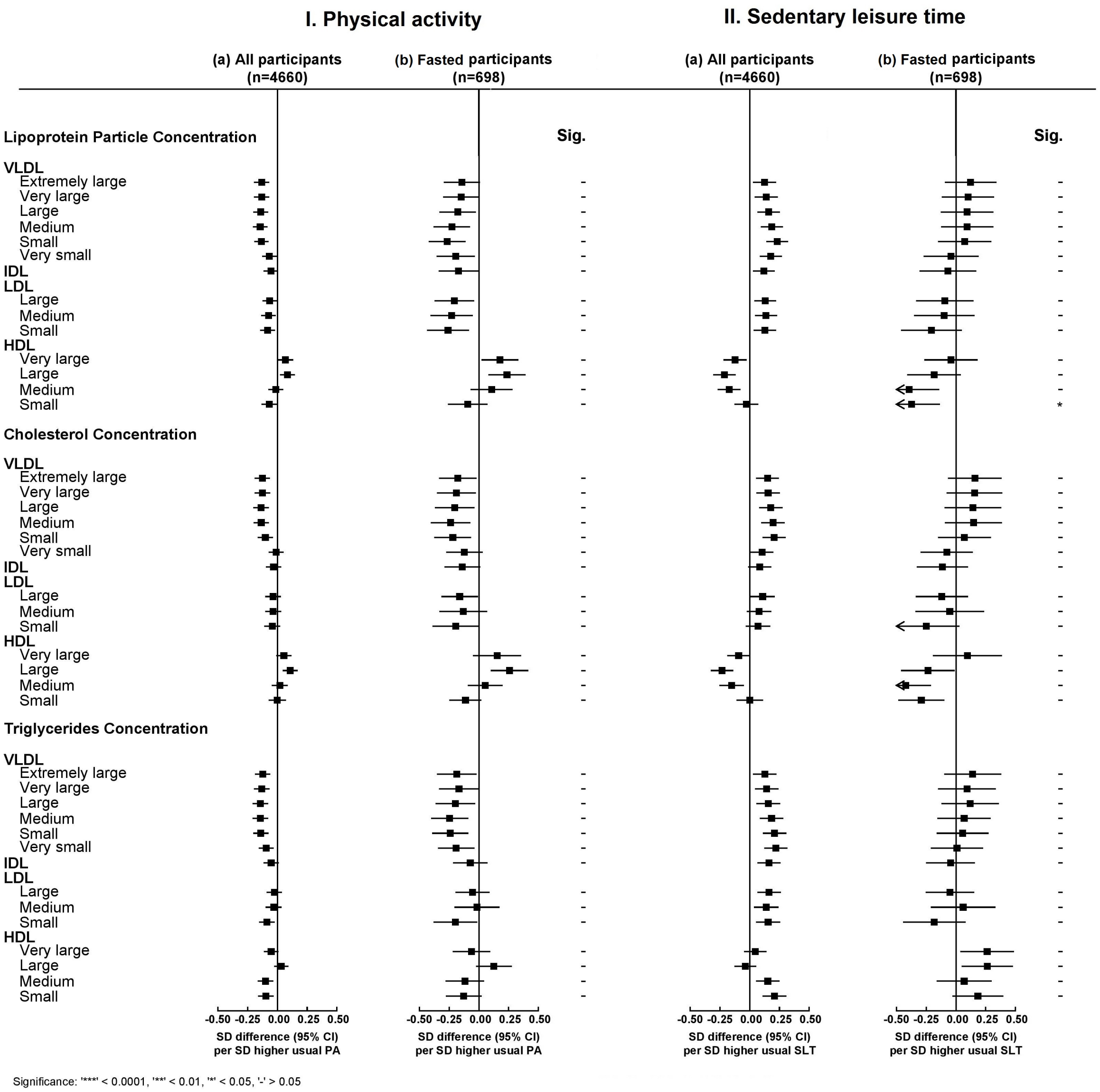

P-values for significance were obtained from heterogeneity tests between fasted and nonfasted participants.

Supplemental Figure 22. Associations of total physical activity and sedentary leisure time with mean particle diameter, cholesterol and triglycerides by fasting status

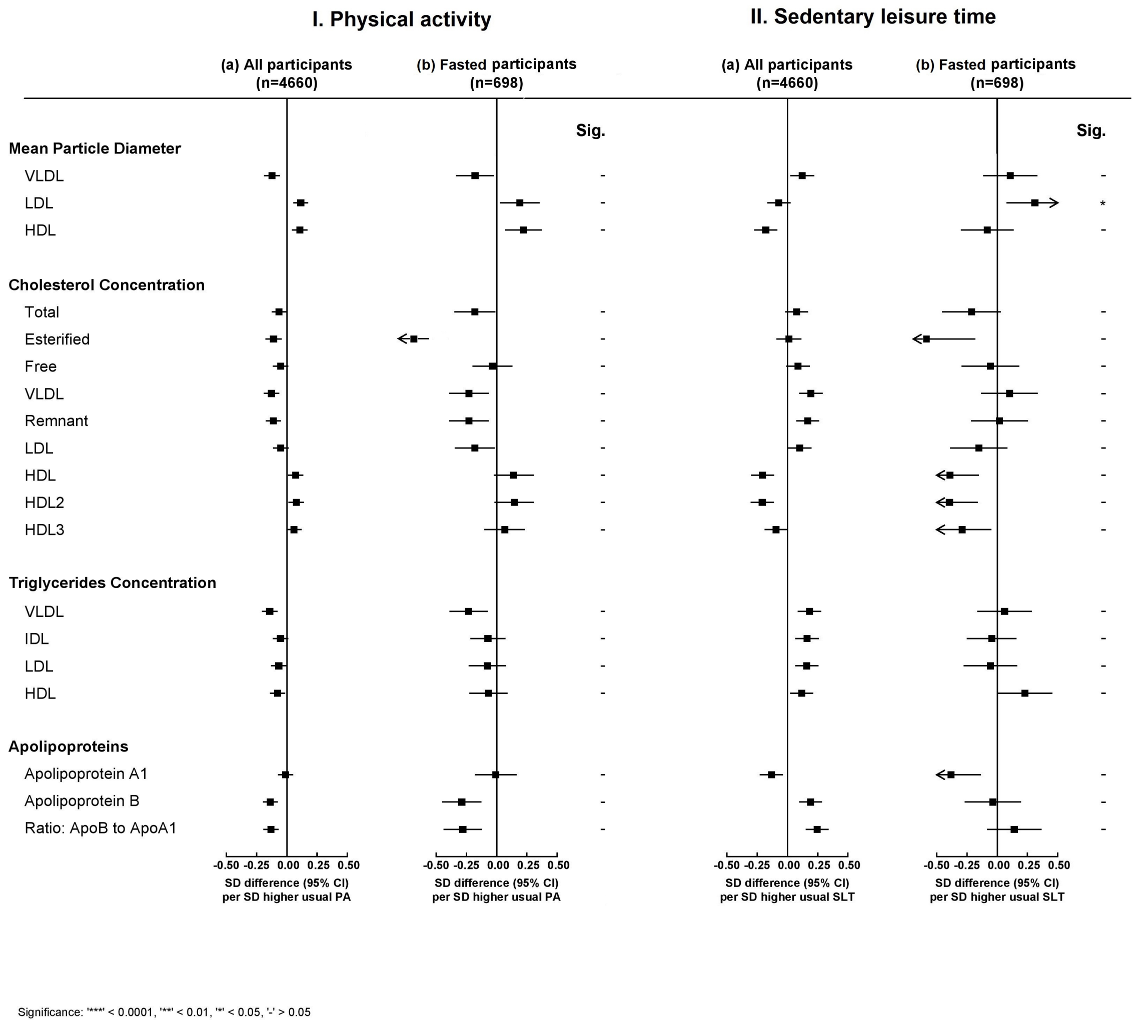

P-values for significance were obtained from heterogeneity tests between fasted and nonfasted participants.

Supplemental Figure 23. Associations of total physical activity and sedentary leisure time with other metabolic traits by fasting status

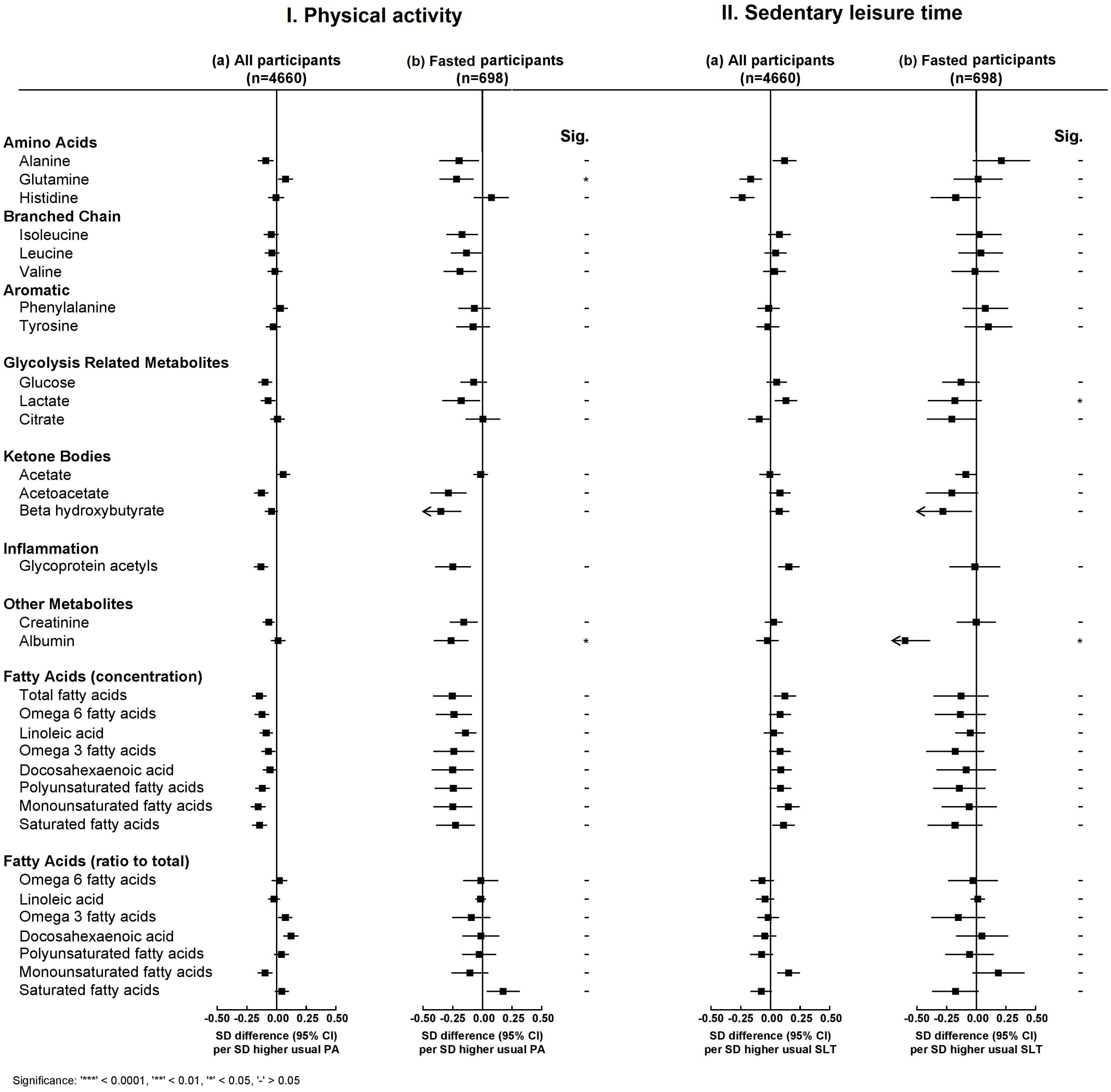

P-values for significance were obtained from heterogeneity tests between fasted and nonfasted participants.

Supplemental Figure 24. Global comparison of adjusted SD differences (95% CI) of clinical chemistry and NMR metabolomics associated with 4 units higher usual total physical activity between men and women

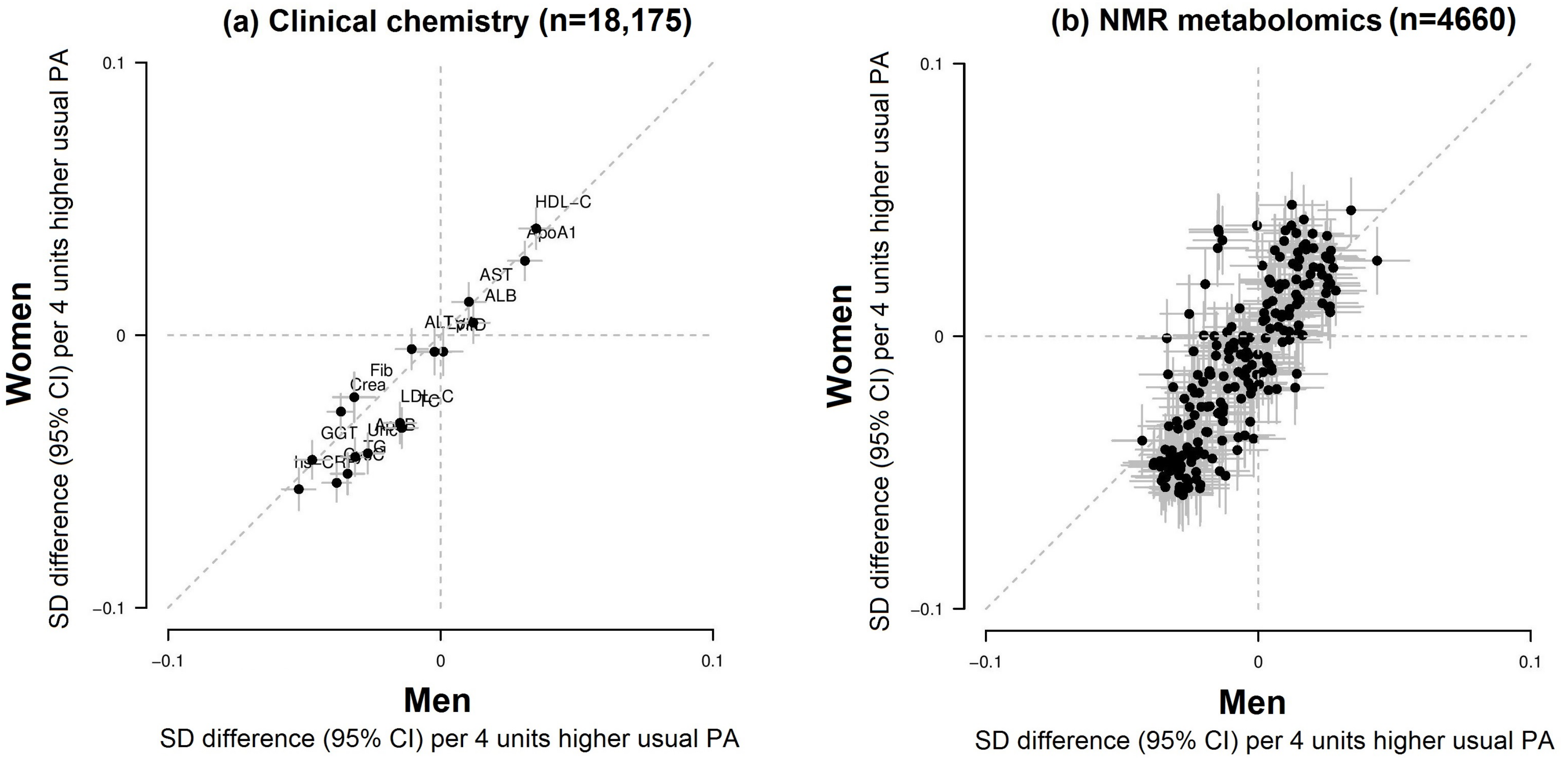

Supplemental Figure 25. Associations of physical activity and sedentary leisure time with lipids and lipoproteins in urban and rural areas, separately

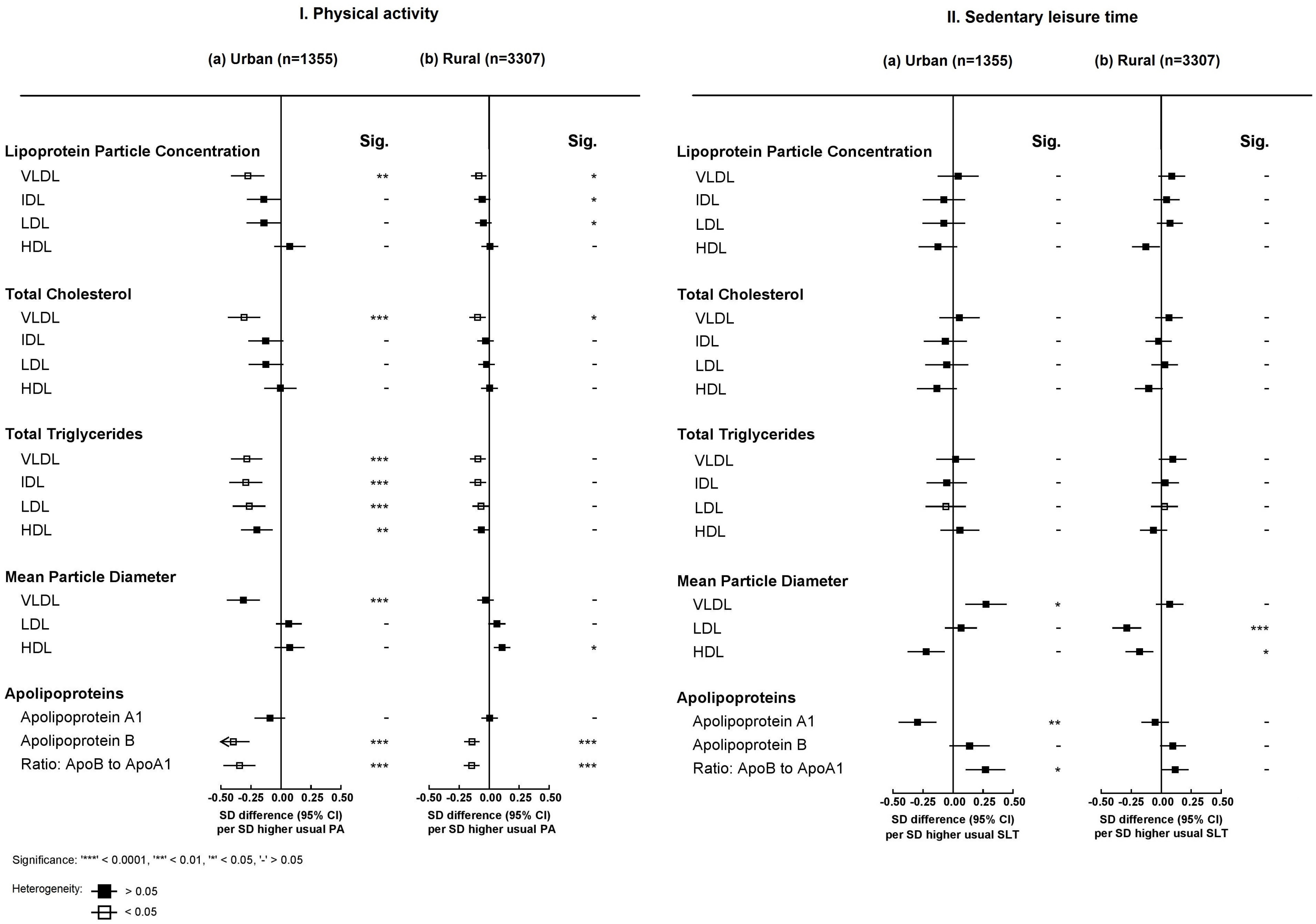

Supplemental Figure 26. Associations of physical activity and sedentary leisure time with other metabolic markers in urban and rural areas, separately

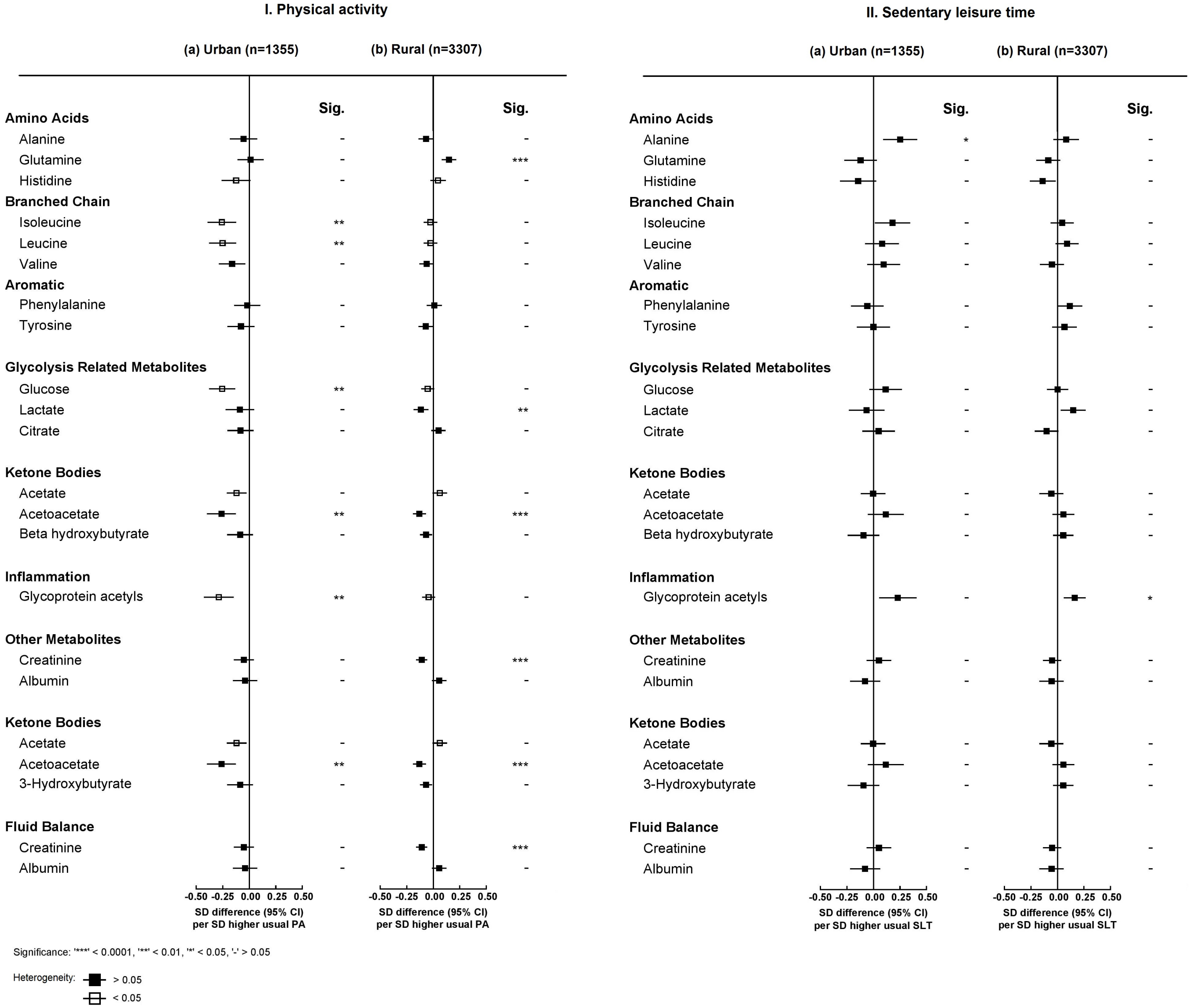

Supplemental Figure 27. Associations of physical activity with lipids and lipoproteins in 10 regions

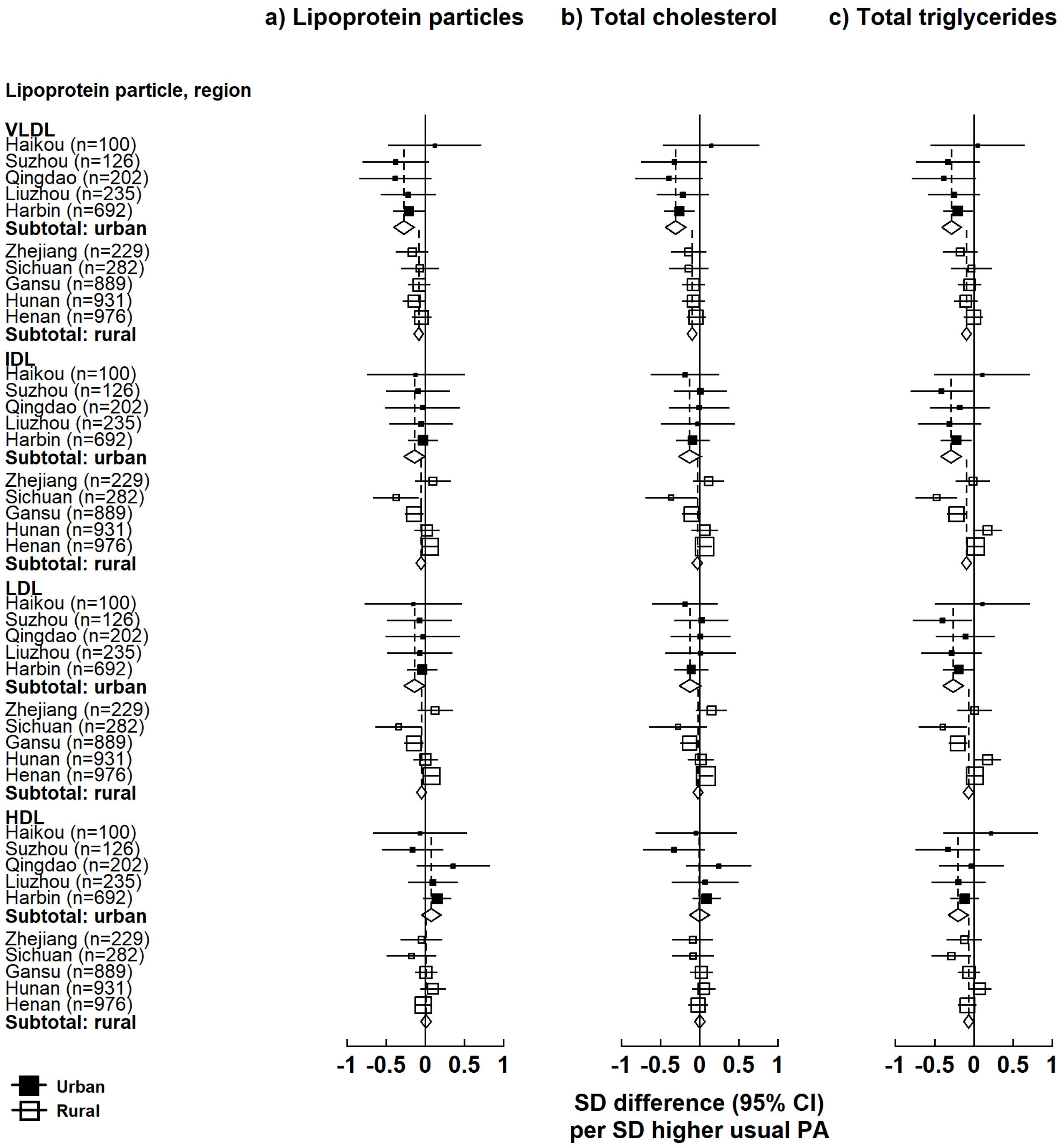

Supplemental Figure 28. Associations of sedentary leisure time with lipids and lipoproteins in 10 regions

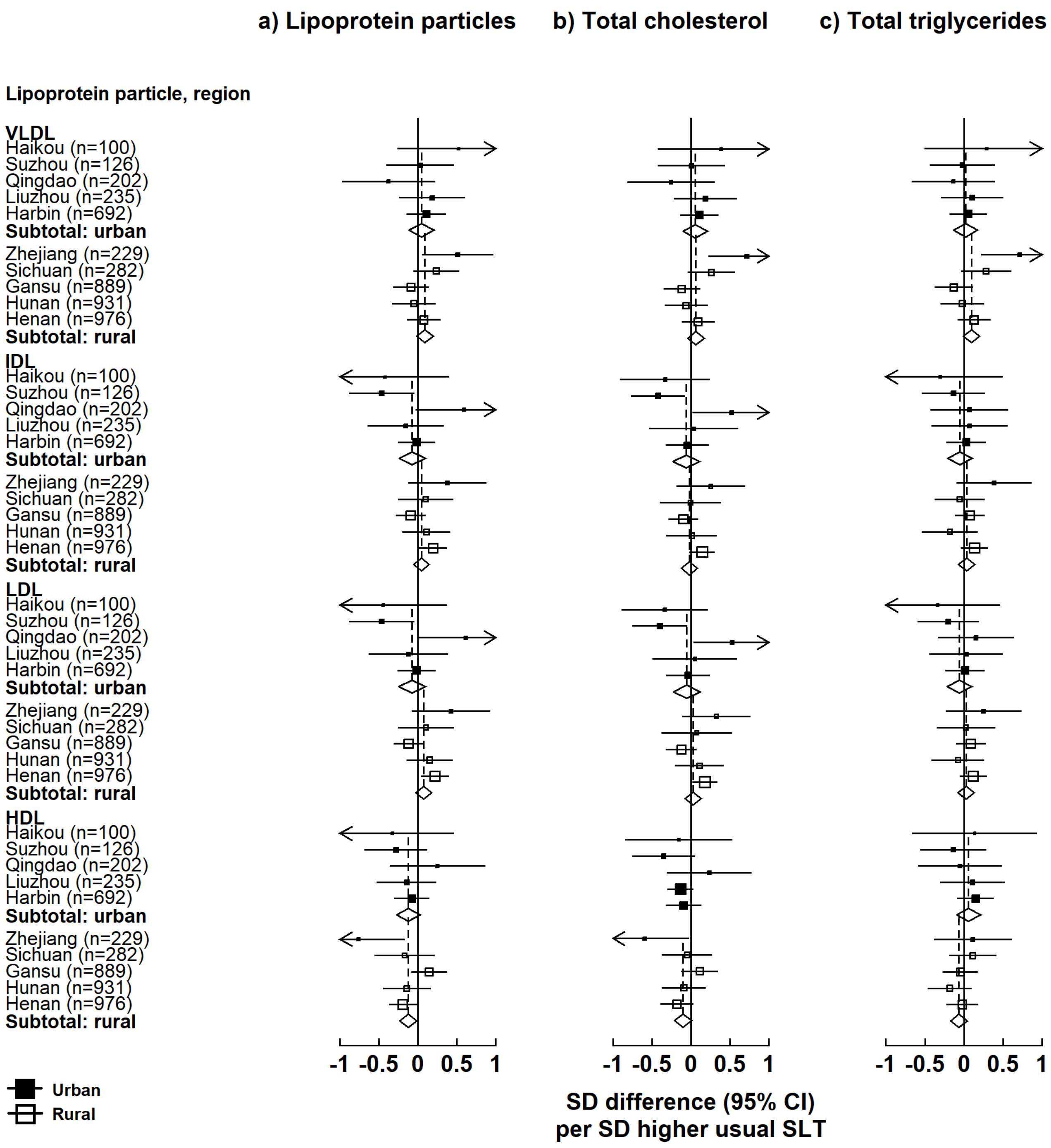

Supplemental Figure 29. Interactions of physical activity (PA) and sedentary leisure time (SLT) on metabolomics

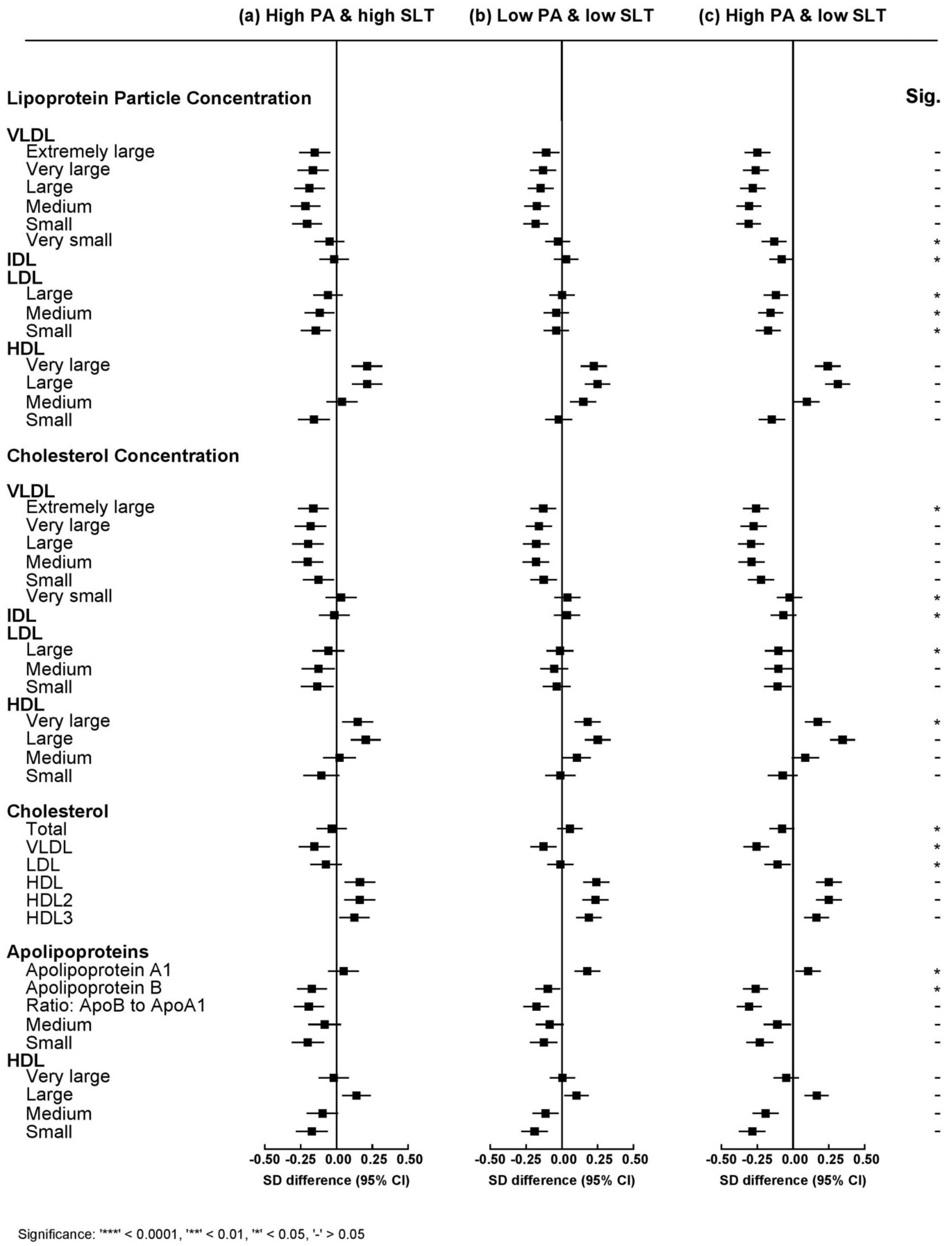

High PA, ≥20 MET-h/day; low PA, <20 MET-h/day; high SLT, ≥3 h/day; low SLT, <3 h/day; reference, low PA & high SLT. P-values for significance of the interaction term were obtained from likelihood ratios tests and were corrected for multiple testing using the Benjamini-Hochberg method.

Supplemental Figure 30. Adjusted hazard ratios (HRs) for major vascular events by levels of usual total physical activity

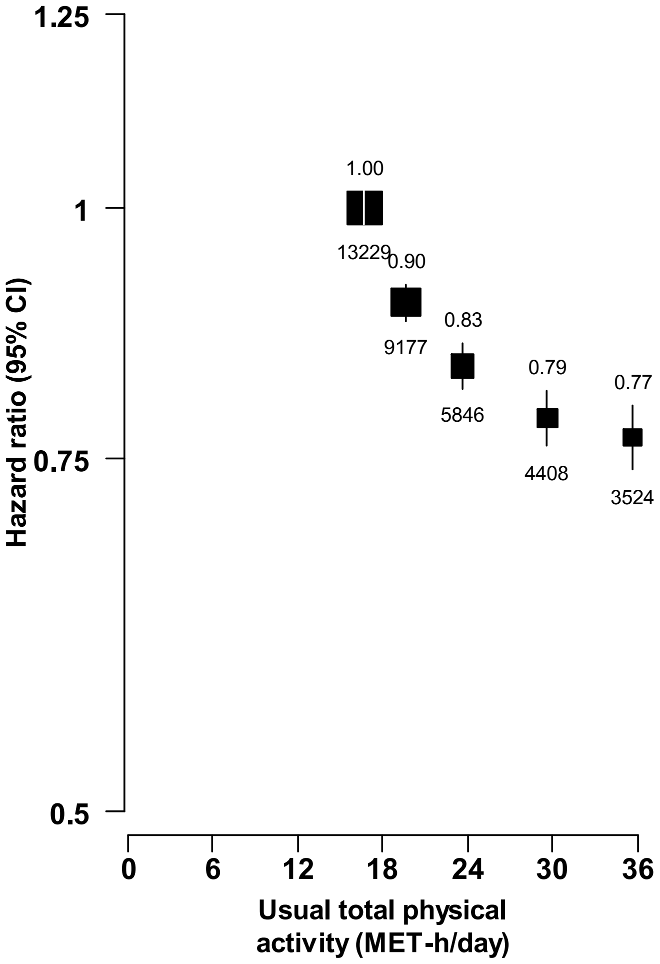

Supplementary Figure 30 is adapted from Bennett et al. (*JAMA Cardiol.* 2: 1349-1358). Values shown are the HR (95% CI) for major vascular events (n=36,184) by quintiles of total physical activity after adjustment for age, sex, region, household income, education, alcohol consumption, smoking, fresh fruit intake, sedentary leisure time, and self-reported general health status. The size of the squares is proportional to the inverse variance of each effect size. The hazard ratio per 4 usual MET-h/day was 0.94 (0.93-0.95). Major vascular events include fatal ischemic heart disease (ICD-10: I20-I25), nonfatal MI (I21-I23), IS (I63), ICH (I61), cardiovascular death (I00-I99), and other nonfatal stroke (I60 and I64).

Reference:
Bennett D, Du H, Clarke R, Guo Y, Yang L, Bian Z, Chen Y, Millwood I, Yu C, He P, et al. Association physical activity and risk of major cardiovascular diseases in Chinese men and women. *JAMA Cardiol*. 2017;2:1349-1358.
