# Supplementary material for: Physical Activity, Sedentary Leisure Time, Circulating Metabolic Markers, and Risk of Major Vascular Diseases
Source: Circ Genom Precis Med. 2019 Sep 17;12(9):e002527. doi: 10.1161/CIRCGEN.118.002527 (PMC6752700; doi:10.1161/CIRCGEN.118.002527)
Supplement: Supplementary file 2 [file hcg-12-e002527-s002.pdf]

Physical Activity, Sedentary Leisure Time, Circulating Metabolic Markers and Risk of Major Vascular Diseases

Running title: *Pang & Kartsonaki et al.; Physical activity, metabolomics and CVD*

Yuanjie Pang, DPhil[1,2]*, Christiana Kartsonaki, DPhil[2,3]*, Huaidong Du, PhD[2,3], Iona Y. Millwood, DPhil[2], Yu Guo, MSc[4], Yiping Chen, DPhil[2,3], Zheng Bian, MSc[4], Ling Yang, PhD[2,3], Robin Walters, PhD[2,3], Fiona Bragg, DPhil[2], Jun Lv, PhD[1], Canqing Yu, PhD[1], Junshi Chen, MD[5], Richard Peto, FRS[2], Robert Clarke, FRCP[2,3], Rory Collins, FRS[2], Derrick A. Bennett, PhD[2], Liming Li, MD[1,4], Michael V. Holmes, PhD[2,3,6]†, Zhengming Chen, DPhil[2]†

[1]School of Public Health, Peking Univ, Beijing, China; [2]Clinical Trial Service Unit & Epidemiological Studies Unit (CTSU), Nuffield Dept of Population Health, [3]Medical Research Council Population Health Research Unit (MRC PHRU), Univ of Oxford, Nuffield Dept of Population Health, Univ of Oxford, Oxford, United Kingdom; [4]Chinese Academy of Medical Sciences; [5]National Center for Food Safety Risk Assessment, Beijing, China; [6]National Institute for Health Research Oxford Biomedical Research Centre, Oxford Univ Hospital, Oxford, United Kingdom

*joint first author / †Joint senior author

American Heart Association

Circulation: Genomic and Precision Medicine

Correspondence:

Associated Prof. Michael V. Holmes
MRC PHRU
NDPH, Big Data Institute Building
University of Oxford
Old Road Campus
Oxford, OX3 7LF, UK
Tel: 44-1865-743644
Fax: 44-1865-743985
E-mail: michael.holmes@ndph.ox.ac.uk

Prof. Zhengming Chen
CTSU
NDPH, Big Data Institute Building
University of Oxford
Old Road Campus
Oxford, OX3 7LF, UK
Tel: 44-1865-743839
Fax: 44-1865-743985
E-mail: zhengming.chen@ndph.ox.ac.uk

Journal Subject Terms: Cardiovascular Disease; Exercise; Risk Factors

Abstract:

Background: Physical inactivity and sedentary behaviour are associated with higher risks of cardiovascular disease (CVD). Little is known about the relevance of circulating metabolites for these associations.

Methods: A nested case-control study within the prospective China Kadoorie Biobank included 3195 incident CVD cases (2057 occlusive CVD and 1138 intracerebral haemorrhage) and 1465 controls aged 30-79 years without prior CVD or statin use at baseline. NMR-spectroscopy measured 225 metabolic markers and derived traits in baseline plasma samples. Linear regression was used to relate self-reported physical activity and sedentary leisure time to biomarkers, adjusting for potential confounders. These were contrasted with associations of biomarkers with occlusive CVD risk.

Results: Physical activity and sedentary leisure time were associated with >100 metabolic markers, with patterns of associations generally mirroring each other. Physical activity was inversely associated with very low and low density- and positively with large and very large high density-lipoprotein particle concentrations. Physical activity was also inversely associated with alanine, glucose, lactate, acetoacetate, and the inflammatory marker glycoprotein acetyls. In general, associations of physical activity and sedentary leisure time with specific metabolic markers were directionally consistent with the associations of these metabolic markers with occlusive CVD risk. Overall metabolic markers potentially explained ~70% of the protective associations of physical activity and ~50% of the positive associations of sedentary leisure time with occlusive CVD.

Conclusions: Among Chinese adults, physical activity and sedentary behaviour have opposing associations with a diverse range of circulating metabolites, which may partially explain their associations with CVD risk.

Key words: physical exercise; metabolomics; cardiovascular disease; China; sedentary leisure time, Chinese

American Heart Association.
Circulation: Genomic and Precision Medicine

Non-standard Abbreviations and Acronyms

25(OH)D, 25-hydroxyvitamin D
ALT, alanine aminotransferase
AST, aspartate aminotransferase
BACC, branched-chain amino acid
BMI, body mass index
CDC, Centre for Disease Control and Prevention
CI, confidence interval
CKB, China Kadoorie Biobank
CVD, cardiovascular disease
FDR, false discovery rate
GGT, gamma glutamyl transferase
HDL-C, high-density lipoprotein cholesterol
hs-CRP, high-sensitivity C-reactive protein
ICH, intracerebral haemorrhage
IDL, intermediate-density lipoprotein
IS, ischaemic stroke
LDL-C, low-density lipoprotein cholesterol
MET, metabolic equivalent of task
MI, myocardial infarction
PA, physical activity
PC, principal component
OR, odds ratio
RCT, randomised controlled trial
RPG, random plasma glucose
SD, standard deviation
SLT, sedentary leisure time
TG, triglyceride
VLDL, very low-density lipoprotein

American Heart Association
Circulation: Genomic and Precision Medicine

Introduction

Low physical activity and prolonged sedentary time, usually measured by self-report, have been independently associated with higher risk of cardiovascular disease (CVD) in diverse populations, including Chinese populations.[1-4] Several biological mechanisms have been proposed that may partially explain these associations, including higher levels of adiposity, blood pressure, and systemic inflammation, as well as unfavourable changes in glucose homeostasis, insulin sensitivity, and lipid and lipoprotein profiles.[5] Observational studies conducted in Western populations have also demonstrated that individuals with higher levels of self-reported physical activity have lower levels of LDL-cholesterol (LDL-C), blood glucose and inflammation (e.g. fibrinogen and C-reactive protein), and higher levels of HDL-cholesterol (HDL-C), with sedentary behaviours generally showing opposite associations.[5-7]

American Heart Association. Circulation: Genomic and Precision Medicine

In addition to conventional biomarkers, there is also emerging evidence that physical activity may be associated with a range of circulating metabolites, including lipids and lipoproteins, amino acids, glycoprotein acetyls, glucose, and fatty acids.[8] These metabolic biomarkers have been shown in a few prospective cohort studies to be associated with risk of occlusive vascular diseases.[9,10] However, the existing evidence on these associations is still limited and no study has yet simultaneously assessed the associations of physical activity and sedentary behaviours with circulating lipids and metabolic markers, and of metabolic markers with CVD risks in the same population. If such associations could be reliably established in different populations, it might shed light on potential mechanisms by which physical activity and sedentary behaviours influence CVD risk. The new Physical Activity Guidelines for Americans provide guidance on the types and amounts of physical activity and but no quantitative guideline for sitting time, and therefore more evidence is needed to assess the cardio-metabolic benefits of

increasing physical activity and decreasing sitting time.[11] Furthermore, reliable assessment of such associations in China is required, because the levels and patterns of physical activity differ importantly from those in Western populations, with, for example, leisure-time physical activity only accounting for ~5% of total physical activity in many parts of China,[12] as opposed to 40% in typical Western populations.[13] Moreover, the mean levels of many blood biomarkers in Chinese (e.g. LDL-C) also differ markedly from Europeans.[14]

The aim of the present study was to examine the associations of self-reported physical activity and sedentary leisure time (as a measure of sedentary behaviours), with plasma lipoproteins, lipids and other metabolic biomarkers in a nested case-control study in the China Kadoorie Biobank (CKB) and to explore how these associations with metabolic markers might explain associations of physical activity and sedentary leisure with vascular disease.

American Heart Association.
Circulation: Genomic and Precision Medicine

Methods

The methods are available in the supplemental data. The CKB data is available at http://www.ckbiobank.org/site/Data+Access. The CKB study was approved by the Ethical Review Committee of the Chinese Center for Disease Control and Prevention and the Oxford Tropical Research Ethics Committee, University of Oxford. All participants eligible for this study had completed a written informed consent form. All methods were performed in accordance with relevant guidelines and regulations.

Results

Among the 4660 participants included, the mean age (SD) was 46 (8) years, 50% were women, the mean level of physical activity was 23 MET-h/day and mean sedentary leisure time was 3.1

h/day (Table 1). The overall mean (SD) LDL-C concentration in controls was 84.9 (27.0) mg/dL. Compared with controls, individuals who subsequently developed MI and IS had lower levels of physical activity, and higher levels of adiposity, systolic blood pressure (SBP), and RPG at baseline, and were more likely to have a history of hypertension or diabetes, while individuals who developed ICH were more likely to have higher SBP and a history of hypertension (Table 1). Individuals with CVD were less likely to consume fresh fruit, fish/seafood, and fresh eggs, and more likely to consume red meat and wheat products (Table 1 and Supplemental Table 1). Characteristics of individuals according to whether they developed CVD (combining MI, IS, and ICH cases) are provided in Supplemental Table 2.

Overall higher total physical activity and sedentary leisure time were associated with >100 metabolic markers at FDR ≤5%, with patterns of associations generally mirroring each other (Figures 1-3). The associations for all 225 traits are shown in Supplemental Table 3. For lipoprotein particle concentrations, physical activity was inversely associated with concentrations of very low-density lipoprotein (VLDL) and more weakly with low-density lipoprotein (LDL) subclasses (Figure 1). For high density-lipoprotein (HDL) particles, physical activity was positively associated with very large and large HDL and inversely with small HDL (Figure 1). Cholesterol within specific lipoprotein particles showed similar associations as the lipoprotein particles (Figure 1). There was an inverse association with remnant cholesterol, but positive associations with cholesterol in HDL particles (Figure 2). Total physical activity was associated with lower triglyceride concentrations in the majority of lipoproteins (Figure 1). Higher physical activity was also associated with smaller VLDL diameter and larger LDL and HDL diameter (Figure 2).

American Heart Association.
Circulation: Genomic and Precision Medicine

There were strong inverse associations of total physical activity with apolipoprotein B and with the ratio of apolipoprotein B to apolipoprotein A1 (Figure 2). For amino acids, physical activity was inversely associated with alanine and positively with glutamine (Figure 3). Physical activity was inversely associated with glucose and lactate (Figure 3), and with acetoacetate (Figure 3). Of the fatty acids, physical activity was inversely associated with absolute concentrations of many different types, but not with ratios of specific fatty acids versus total fatty acids, except monounsaturated fatty acids (Figure 3). Total physical activity was also inversely associated with glycoprotein acetyls (Figure 3), a marker of inflammation. As shown in Supplemental Figure 1, the associations were linear for total cholesterol, triglycerides and apolipoprotein B whereas for HDL-C, apolipoprotein A1, creatinine, albumin and to a lesser extent LDL-C, threshold effects were evident at higher levels of PA. In addition, the associations were approximately linear for VLDL and HDL lipoprotein particles, VLDL-C, HDL-C, VLDL triglycerides, glucose, and glycoprotein acetyls (Supplemental Figure 2).

American Heart Association
Circulation: Genomic and Precision Medicine

Similar associations were observed when we compared the associations of total physical activity with 8 traits measured by both clinical chemistry and NMR metabolomics (Supplemental Figure 3). Overall, among 18,175 participants with 17 biomarkers measured by clinical chemistry (Supplemental Figure 1 and Supplemental Figure 4), there were strong inverse associations of total physical activity with total cholesterol (difference per 4 units (equivalent to 1 hour of moderate physical activity such as brisk walking [3-4 mph]): -35 [95% CI: -49 to -22] mg/dL), LDL-C (-18 [-25 to -11] mg/dL), total triglycerides (-86 [-982 to -7.5] mg/dL), apolipoprotein B, and creatinine, and positive associations with HDL-C (4.4 [3.1 to 5.6] mg/dL), and apolipoprotein A1. There was also a positive trend for albumin. Moreover, total physical activity was inversely associated with hs-CRP, fibrinogen, cystatin C, ALT, GGT, and uric acid,

and positively associated with AST (Supplemental Figure 4). Opposite patterns were observed for the associations between sedentary leisure time and clinical chemistry (Supplemental Figure 5).

In this nested case-control study, total physical activity showed an inverse association with occlusive CVD (OR per 1-SD 0.86 [0.75-0.99]) following adjustment for age, sex, fasting time, region, smoking, education, income, self-rated health, intake of fresh fruit and red meat, and sedentary leisure time, whereas sedentary leisure time showed a positive association with occlusive CVD (OR per 1-SD 1.28 [1.02-1.60], adjusting for physical activity plus the same covariates except for sedentary leisure time). There was a clear pattern of the associations of total physical activity with metabolic markers, and metabolic markers with disease risk, i.e. metabolic markers that were associated with higher total physical activity tended to be associated with lower risk of occlusive CVD (Pearson correlation coefficient = -0.76; Figure 4). Further analyses showed that the 18 PCs which explained ≥95% of the variation across the 225 traits potentially explained ~70% of the effect of total physical activity and ~50% of the effect of sedentary leisure time on risk of occlusive CVD (Supplemental Table 4). For sedentary leisure time, metabolic markers that associated with higher sedentary leisure time tended to associate with higher risk of occlusive CVD (Pearson correlation coefficient = 0.72; Figure 4).

American Heart Association.
Circulation: Genomic and Precision Medicine

In sensitivity analyses, the associations with metabolic markers were similar for occupational and non-occupational physical activity, although the magnitude of effects appeared somewhat stronger for the latter (Supplemental Figures 6-8), and heterogeneity tests showed no evidence of any difference except for triglycerides in large HDL (FDR-adjusted *p* for heterogeneity 0.02). Similar associations were evident 1) when restricting the analyses to controls (Supplemental Figures 9-11), 2) when further adjusting for BMI, diabetes, other dietary

factors, or medications (Supplemental Figures 12-17), 3) when excluding major chronic diseases and poor self-rated health at baseline (Supplemental Figures 18-20), 4) in all and fasted participants (Supplemental Figures 21-23), and 5) in both men and women (Supplemental Figure 24). When comparing participants from urban and rural areas (Supplemental Figures 25-26), the patterns were generally similar except for greater magnitudes of associations for physical activity with several traits in urban areas, including VLDL lipoprotein particles, VLDL cholesterol, triglycerides in VLDL, IDL, and LDL, apolipoprotein B, isoleucine, leucine, glucose, and glycoprotein acetyls. When comparing lipids and lipoproteins across 10 regions (Supplemental Figures 27-28), there was no evidence of any difference between regions except for triglycerides in IDL and LDL (*p* for heterogeneity <0.05). When examining the combined effects of high total physical activity and low sedentary leisure time on metabolomics, significant interactions were observed for lipoprotein concentrations of very small VLDL, IDL, LDL (large, medium, small), cholesterol concentrations in extremely large and very small VLDL, IDL, large LDL, very large HDL, total cholesterol, VLDL-C, LDL-C, apolipoprotein A1, and apolipoprotein B (Supplemental Figure 29). In cases where an interaction was present, the combination of high physical activity and low sedentary leisure time had stronger associations with lower concentrations of atherogenic lipoproteins, their cholesterol concentrations and apolipoprotein B in comparison to other groups.

American Heart Association
Circulation: Genomic and Precision Medicine

Discussion

This study comprehensively examined the associations of self-reported total, occupational, non-occupational physical activity and sedentary leisure time with a wide range of metabolic biomarkers, and the potential role of these biomarkers in explaining the association between

physical activity, sedentary leisure time, and risk of major occlusive CVD. In this Chinese population, there were inverse associations of physical activity with VLDL and LDL and positive associations with HDL particle concentrations. In addition, total physical activity was inversely associated with inflammation markers including hs-CRP, fibrinogen, and glycoprotein acetyls. Although the associations of sedentary leisure time with metabolic markers generally mirrored those of total physical activity, a few traits showed different associations (e.g. histidine). Importantly, we showed that the global difference in metabolic biomarkers related to higher physical activity conferred lower risk of occlusive CVD, with an approximately 70% of the protective association potentially explained by these metabolic markers. These metabolic markers also potentially explained ~50% of the harmful effect of sedentary leisure time on occlusive CVD. These results further extended prospective findings in our study showing inverse associations of physical activity with major vascular events (Supplemental Figure 30).[2]

American Heart Association
Circulation: Genomic and Precision Medicine

To date, three observational studies have previously assessed the associations of physical activity with circulating metabolic markers in adult populations.[8,15,16] While the overall findings were generally similar to those in the present study, they tended to focus on leisure time physical activity and did not examine the association of metabolic markers with disease risk, nor associations of physical activity with disease risk. In a twin-pair and three population-based cohort studies in Finland (16 twin pairs, Pieksämäki cohort, Young Finns Study, and Northern Finland Birth Cohort 1966) with 136 metabolic markers measured using the same NMR platform,[8] self-reported active individuals had lower concentrations, in comparison with inactive individuals, of the apolipoprotein-B-containing lipoproteins (VLDL, LDL, and IDL) and their cholesterol concentrations, and higher concentrations of large HDL and their cholesterol concentrations, but not small HDL. Moreover, physically active individuals had on average

lower concentrations of glucose, glycoprotein acetyls, and alanine. The present study showed similar findings for lipoprotein particles, lipid constituents, amino acids, and other metabolic biomarkers. In particular, similar findings for LDL particles and LDL-C were observed when comparing the associations for non-occupational physical activity in CKB with the associations for leisure-time physical activity in the Finnish study, although the mean levels of LDL-C in our study populations were lower. Consistent with our association of physical activity and HDL particles, randomised controlled trials (RCTs) suggest that exercise training increases large HDL particles and HDL particle size and decreases small HDL particles.[17,18] Consistent with the Finnish study, the associations between physical activity and metabolomics persisted when additionally adjusting for BMI, while the present study extended this to show that the associations between physical activity and metabolites persisted even with additional adjustment for diabetes. As well as showing similar patterns of physical activity as in the Finnish study,[8] the present study further characterised the associations of sedentary leisure time and showed opposite associations with lipids and lipoproteins, amino acids, and inflammation.

American Heart Association
Circulation: Genomic and Precision Medicine

Isoleucine, leucine, and valine are part of the branched-chain amino acid (BCAA) group, essential amino acids that play a key role in energy production and protein synthesis.[19] Circulating levels of BCAAs are associated with obesity, insulin resistance, metabolic disorders, type 2 diabetes, and CVD.[19] The findings in CKB were directionally consistent with a small Chinese study (n=277) assessing physical activity by accelerometers and a Japanese study (n=1193) by questionnaires, both of which reported inverse associations of total physical activity with isoleucine, leucine, and valine, [15,16] as well as positive associations of sedentary leisure time with these BCAAs.

In addition to quantifying the associations of physical activity with metabolic markers, this study examined the extent to which hundreds of metabolic markers potentially explained the associations between physical activity and CVD risk. One study in the US with 27,000 participants and 979 incident CVD (mainly MI and IS) showed that conventional lipids (LDL-C, HDL-C, and total cholesterol) and inflammation (hs-CRP, fibrinogen, and sICAM-1) each accounted for 19% and 32% of the protective effect of leisure-time physical activity on CVD risk.[20] In CKB, we identified a pattern whereby physical activity tended to associate with lower concentrations of metabolic markers that were associated with a higher risk of occlusive CVD and vice versa. Compare with the US study, our study reported a larger proportion of this protective effect explained by lipids (LDL-C, HDL-C, and total cholesterol: 34%) and a similar proportion by inflammation (hs-CRP and fibrinogen: 30%). In CKB, a similar proportion of the effect of sedentary leisure time on occlusive CVD risk was potentially explained by inflammation (hs-CRP and fibrinogen: 35%), while a larger proportion was explained by lipids (LDL-C, HDL-C, and total cholesterol: 60%) than physical activity. However, among these metabolic biomarkers assessed, HDL-C, hs-CRP, and fibrinogen are unlikely to be causally associated with CVD, as suggested by Mendelian randomisation studies.[21,22] Atherogenic lipoproteins including VLDL and LDL are almost certainly causally related to CVD, while recent evidence has suggested that glycoprotein acetyls may mark inflammation pathways relevant to the development of CVD.[9] In our study, glycoprotein acetyls alone potentially explained 25% of the total effect of physical activity on CVD, while VLDL-C and LDL-C each explained 13% and 10%, respectively (Supplemental Table 4). Glycoprotein acetyls are the products of glycosylation modification of secreted inflammatory proteins, which are correlated with other acute phase reactants including CRP and fibrinogen.[10,23] In our study, the inverse

American Heart Association
Circulation: Genomic and Precision Medicine

association of total physical activity with glycoprotein acetyls was consistent with the inverse associations with hs-CRP and fibrinogen as measured by clinical chemistry assay, in agreement with previous cross-sectional studies (~70K participants) in Western populations that assessed leisure-time physical activity.[5-7] Inflammation pathways may therefore represent a promising mechanism to explain the protective association between physical activity and CVD. Indeed, previous studies have reported that higher levels of glycoprotein acetyls were associated with incident type 2 diabetes, hypertension, and CVD.[10,23]

The associations that we report potentially implicate lipids and lipoproteins as mediators of the beneficial effects of physical activity on risk of cardiovascular disease. This is plausible because physical activity increases the mitochondrial density in skeletal muscle, leading to higher metabolism of VLDL triglycerides and fatty acids. Notably, these patterns of association are shown in our data, with higher physical activity associated with lower concentrations of VLDL particles, VLDL cholesterol and VLDL triglycerides, and higher sedentary leisure time associated with higher concentrations of these biomarkers. It is also noteworthy that these associations of physical activity and sedentary leisure time were seen across the lipoprotein and lipid cascade, despite the average LDL-C of our dataset being relatively low (2.4 mmol/L). Consistent with previous observational studies in China[24] and elsewhere (mean LDL-C 4.4-4.5 mmol/L)[14], randomised controlled trials have shown that lowering of LDL-C reduced the risk of major vascular events even among individuals with so-called normal or low cholesterol levels.[25]

Our findings are of contemporary importance given the recent publication of guidelines that seek to increase the amounts of physical activity that individuals undertake in order to prevent cardiovascular disease. For example, the Physical Activity Guidelines for Americans advise more physical activity and less sedentary leisure time as both provide health benefits as

American Heart Association
Circulation: Genomic and Precision Medicine

assessed by all-cause and CVD mortality, providing recommendations on the types and amounts of physical activity across age groups. Of note, no quantitative guideline is provided for sitting time.[11] More recently, the American College of Cardiology/American Heart Association (ACC/AHA) published guidelines for the primary prevention of CVD, which included advice for adults to engage in at least 150 minutes of moderate-intensity physical activity (or 75 minutes of vigorous-intensity physical activity).[26] The ACC/AHA guidelines also advise decreasing sedentary behaviour in adults to lower risk of CVD but this recommendation is based on limited data. Our study quantifies the associations of physical activity and sitting time with circulating metabolites and CVD in adult populations. More importantly, we showed similar patterns for occupational and non-occupational physical activity, which reinforced current Chinese guidelines that promote any type of physical activity for CVD prevention.[27]

American Heart Association
Circulation: Genomic and Precision Medicine

The strengths of the CKB included large numbers of well-characterised CVD events, assessment of different activity measures (i.e. total, occupational, non-occupational physical activity, and sedentary leisure time) with a broad range of blood-based metabolic markers, and the excellent concordance of NMR measurements compared to conventional clinical chemistry approaches.[10] Our study also had several limitations. First, although similar associations were observed after further adjustment for possible confounders or mediators including BMI, diabetes, and dietary variables, residual confounding because of unmeasured or sub-optimally measured factors (e.g. diet) may still be present. Second, physical activity and sedentary leisure were self-reported, and therefore measurement error may exist due to subjective reporting and between-person differences in intensity of physical activity. However, we corrected for regression dilution using the resurvey data when assessing the associations of physical activity and sedentary leisure time with metabolic markers, which may partially account for random measurement error and

within-person variability. In addition, the patterns between total physical activity and metabolomics observed in the current study were generally consistent with a recent study of 1826 adolescents assessing device-measured physical activity and NMR metabolomics, particularly for cholesterol and triglycerides in HDL and VLDL and glycoprotein acetyls.[28] That study also showed opposite associations for sedentary leisure time, but the associations for other metabolic biomarkers were weaker or nonsignificant compared with those in CKB. Third, we used PCA to account for the large number of correlated metabolic markers when we calculated the proportion of associations of physical activity and sedentary leisure time with CVD potentially explained by all metabolic markers. Therefore, it is difficult to fully disentangle which biomarkers, either individually or in combination, chiefly explained the effects of physical activity on CVD, especially given that the associations of physical activity and metabolic biomarkers were based on cross-sectional analyses.

American Heart Association. Circulation: Genomic and Precision Medicine

Conclusions

Our study set in China shows that higher physical activity was associated with lower concentrations of atherogenic lipoproteins and cholesterol, and lower levels of inflammation, and in general, these metabolic markers were associated with risk of occlusive CVD.[10] Opposing patterns of associations between sedentary leisure time and metabolic markers were observed. This suggests that physical activity may result in favourable alterations to blood-based lipids and metabolic markers that might partly explain the relationship between physical activity and CVD. Our findings provide insights into the biological mechanisms linking physical activity, sedentary leisure time and CVD.

Acknowledgments: The chief acknowledgment is to the participants, the project staff, and the China National Centre for Disease Control and Prevention (CDC) and its regional offices for

access to death and disease registries. The Chinese National Health Insurance scheme provided electronic linkage to all hospital admission data.

Sources of Funding: Baseline survey: Kadoorie Charitable Foundation, Hong Kong. Long-term continuation: UK Wellcome Trust (088158/Z/09/Z, 104085/Z/14/Z), Chinese Ministry of Science and Technology (2011BAI09B01, 2012-14), Chinese National Natural Science Foundation (81390541). The British Heart Foundation, UK Medical Research Council and Cancer Research UK provide core funding to the Oxford CTSU. NMR metabolomics was supported by the BHF Cntre of Research Excellence, Oxford (RE/13/1/30181). Dr Bragg acknowledges support from the BHF Centre of Research Excellence, Oxford. Dr Holmes is supported by a British Heart Foundation Intermediate Clinical Research Fellowship (FS/18/23/33512) and the National Institute for Health Research Oxford Biomedical Research Centre.

American Heart Association.

Disclosures: None.

Circulation: Genomic and Precision Medicine

References:

1. Sattelmair J, Pertman J, Ding EL, Kohl HW 3rd, Haskell W, Lee IM. Dose response between physical activity and risk of coronary heart disease: a meta-analysis. *Circulation.* 2011;124:789-795.

2. Bennett D, Du H, Clarke R, Guo Y, Yang L, Bian Z, Chen Y, Millwood I, Yu C, He P, et al. Association physical activity and risk of major cardiovascular diseases in Chinese men and women. *JAMA Cardiol*. 2017;2:1349-1358.

3. Lear SA, Hu W, Rangarajan S, Gasevic D, Leong D, Iqbal R, Casanova A, Swaminathan S, Anjana RM, Kumar R, et al. The effect of physical activity on mortality and cardiovascular disease in 130 000 people from 17 high-income, middle-income, and low-income countries: the PURE study. *Lancet.* 2017;390:2643-2654.

4. Ford ES, Caspersen CJ. Sedentary behaviour and cardiovascular disease: a review of prospective studies. *Int J Epidemiol.* 2012;41:1338-1353.

5. Warburton DE, Nicol CW, Bredin SS. Health benefits of physical activity: the evidence. *CMAJ.* 2006;174:801-809.

6. Fung TT, Hu FB, Yu J, Chu NF, Spiegelman D, Tofler GH, Willett WC, Rimm EB. Leisure-

time physical activity, television watching, and plasma biomarkers of obesity and cardiovascular disease risk. *Am J Epidemiol.* 2000;152:1171-1178.

7. Mora S, Lee IM, Buring JE, Ridker PM. Association of physical activity and body mass index with novel and traditional cardiovascular biomarkers in women. *JAMA.* 2006;295:1412-1419.

8. Kujala UM, Mäkinen VP, Heinonen I, Soininen P, Kangas AJ, Leskinen TH, Rahkila P, Würtz P, Kovanen V, Cheng S, et al. Long-term leisure-time physical activity and serum metabolome. *Circulation.* 2013;127:340-348.

9. Würtz P, Havulinna AS, Soininen P, Tynkkynen T, Prieto-Merino D, Tillin T, Ghorbani A, Artati A, Wang Q, Tiainen M, et al. Metabolite profiling and cardiovascular event risk: a prospective study of 3 population-based cohorts. *Circulation.* 2015;131:774-785.

10. Holmes MV, Millwood IY, Kartsonaki C, Hill MR, Bennett DA, Boxall R, Guo Y, Xu X, Bian Z, Hu R, et al. Lipids, lipoproteins, and metabolites and risk of myocardial infarction and stroke. *J Am Coll Cardiol.* 2018;71:620-632.

11. Piercy KL, Troiano RP, Ballard RM, Carlson SA, Fulton JE, Galuska DA, George SM, Olson RD. The physical activity guidelines for Americans. *JAMA.* 2018;320:2020-2028.

12. Du H, Bennett D, Li L, Whitlock G, Guo Y, Collins R, Chen J, Bian Z, Hong L, Feng S, et al. Physical activity and sedentary leisure time and their associations with BMI, waist circumference, and percentage body fat in 0.5 million adults: the China Kadoorie Biobank study. *Am J Clin Nutr.* 2013;97:487-496.

13. Hallal PC, Andersen LB, Bull FC, Guthold R, Haskell W, Ekelund U; Lancet Physical Activity Series Working Group Global physical activity levels: surveillance progress, pitfalls, and prospects. *Lancet.* 2012;380:247-257.

14. Emerging Risk Factor Collaboration. Major lipids, apolipoproteins, and risk of vascular disease. *JAMA.* 2009;302:1993-2000.

15. Xiao Q, Moore SC, Keadle SK, Xiang YB, Zheng W, Peters TM, Leitzmann MF, Ji BT, Sampson JN, Shu XO, et al. Objectively measured physical activity and plasma metabolomics in the Shanghai Physical Activity Study. *Int J Epidemiol.* 2016;45:1433-1444.

16. Fukai K, Harada S, Iida M, Kurihara A, Takeuchi A, Kuwabara K, Sugiyama D, Okamura T, Akiyama M, Nishiwaki Y, et al. Metabolic profiling of total physical activity and sedentary behavior in community-dwelling men. *PloS One.* 2016;11:e0164877. doi: 10.1371/journal.pone.0164877.

17. Halverstadt A, Phares DA, Wilund KR, Goldberg AP, Hagberg JM. Endurance exercise training raises high-density lipoprotein cholesterol and lowers small low-density lipoprotein and very low-density lipoprotein independent of body fat phenotypes in older men and women. *Metabolism.* 2007;56:444-450.

American Heart Association
Circulation: Genomic and Precision Medicine

18. Khan AA, Mundra PA, Straznicky NE, Nestel PJ, Wong G, Tan R, Huynh K, Ng TW, Mellett NA, Weir JM, et al. Weight loss and exercise alter the high-density lipoprotein lipidome and improve high-density lipoprotein functionality in metabolic syndrome. *Arterioscler Thromb Vasc Biol.* 2018;38:438-447.

19. Batch BC, Hyland K, Svetkey LP. Branch chain amino acids: biomarkers of health and disease. *Curr Opin Clin Nutr Metab Care.* 2014;17:86-89.

20. Mora S, Cook N, Buring JE, Ridker PM, Lee IM. Physical activity and reduced risk of cardiovascular events. *Circulation.* 2007;116:2110-2118.

21. Holmes MV, Ala-Korpela M, Smith GD. Mendelian randomization in chardiometabolic disease: challenges in evaluating causality. *Nat Rev Cardiol.* 2017;14:577-590.

22. Interleukin-6 Receptor Mendelian Randomisation Analysis (IL6R MR) Consortium. The Interleukin-6 receptor as a target for prevention of coronary heart disease: a mendelian randomisation analysis. *Lancet.* 2012;379:1214-1224.

23. Engstrom G, Hedblad B, Stavenow L, Jonsson S, Lind P, Janzon L, Lindgärde F. Incidence of obesity-associated cardiovascular disease is related to inflammation-sensitive plasma proteins: a population-based cohort study. *Arterioscler Thromb Vasc Biol.* 2004;24:1498-1502.

24. Chen Z, Peto R, Collins R, MacMahon S, Lu J, Li W. Serum cholesterol concentration and coronary heart disease in population with low cholesterol concentrations. *BMJ.* 1991;303:276-282.

25. Study of the Effectiveness of Additional Reductions in Cholesterol and Homocysteine (SEARCH) Collaborative Group. Intensive lowering of LDL cholesterol with 80 mg versus 20 mg simvastatin daily in 12,064 survivors of myocardial infarction: a double-blind randomised trial. *Lancet.* 2010;379:1658-1669.

26. Arnett DK, Blumenthal RS, Albert MA, Buroker AB, Goldberger ZD, Hahn EJ, Himmelfarb CD, Khera A, Lloyd-Jones D, McEvoy JW, et al. 2019 ACC/AHA guideline on the primary prevention of cardiovascular disease. *Circulation.* 2019 Mar 17. doi: 10.1161/CIR.0000000000000678.

27. Ministry of Health of the People's Republic of China. Physical activity guidelines for Chinese adults (on trial). Beijing: People's Medical Publishing House; 2011.

28. Bell JA, Hamer M, Richmond RC, Timpson NJ, Carslake D, Davey Smith G. Associations of device-measured physical activity across adolescence with metabolic traits: prospective cohort study. *PLoS Med.* 2018;15:e1002649. doi:10.1371/journal.pmed.1002649.

Circulation: Genomic and Precision Medicine

Table 1. Baseline characteristics of participants in the nested case-control study among participants without prior CVD

Variable*	MI/IS cases (n=2057)	ICH cases (n=1138)	Controls (n=1465)	All (n=4660)
Age (SD), year	46.2 (8.2)	47.0 (6.9)	45.0 (8.3)	46.2 (8.0)
Female, %	49.7	52.2	52.3	50.2
**Socioeconomic and lifestyle factors, %**				
Urban residents	33.7	23.5	24.9	29.1
≥9 years of education	21.1	21.1	24.4	22.1
Household income ≥35,000 RMB/year	12.9	10.1	10.4	11.5
Ever regular smoking				
Male	76.3	69.8	67.8	72.5
Female	2.1	2.0	1.8	2.1
Weekly drinking				
Male	32.3	36.8	33.3	34.4
Female	2.1	2.6	1.6	2.0
Total physical activity (SD), MET h/day	22.5 (14.0)	23.0 (14.6)	24.6 (14.5)	23.0 (14.4)
Sedentary leisure time (SD), h/d	3.1 (1.4)	3.1 (1.5)	3.0 (1.4)	3.1 (1.5)
**Blood pressure, blood glucose and anthropometry**				
SBP (SD), mmHg	136.9 (24.9)	150.7 (29.0)	126.8 (17.8)	137.3 (25.7)
RPG (SD), mmol/L	6.3 (3.1)	6.2 (3.1)	5.6 (1.9)	6.1 (2.8)
BMI (SD), kg/m^2	24.2 (3.7)	24.0 (3.6)	23.4 (3.2)	24.0 (3.5)
**Daily consumption, %**				
Fresh fruit	12.8	11.9	15.1	13.3
Red meat	26.8	24.6	23.6	25.8
Fish/seafood	1.6	1.2	2.0	1.7
Fresh eggs	13.8	13.1	14.5	14.1
Wheat product	57.0	57.3	54.0	56.2
**Prior disease history, %**				
Hypertension	15.0	22.7	5.5	14.3
Diabetes	8.3	6.3	3.1	6.5
**Family disease history, %**				
Diabetes	6.9	5.6	5.4	6.3
CVD	14.3	12.4	17.3	14.2

* Results were standardised by age, sex, and area (where appropriate). Values are means unless otherwise stated. Abbreviations: %BF=percent body fat, BMI=body mass index, CHD=coronary heart disease, CVD=cardiovascular disease, ICH=intracerebral haemorrhage, IS=ischemic stroke, MET=metabolic equivalent of task, MI=myocardial infarction, RPG=random plasma glucose, SBP=systolic blood pressure, TIA=transient ischaemic attack.

American Heart Association
Circulation: Genomic and Precision Medicine

Figure Legends:

Figure 1. Associations of usual total physical activity (PA) and sedentary leisure time (SLT) with lipoprotein particle concentration, cholesterol and triglycerides and of these metabolic markers with risks of occlusive CVD. Column (a) shows adjusted SD differences (95% CI) of log-transformed metabolic markers per 1-SD higher usual total physical activity, and column (b) shows corresponding estimates per 1-SD higher usual sedentary leisure time. Column (c) shows adjusted ORs (95% CI) of CVD risk (MI and IS) per 1-SD higher log-transformed metabolic markers. Models were adjusted for age, sex, fasting time, region, smoking status, education, income, self-rated health, intake of fruit and meat, sedentary leisure time (for total physical activity), and total physical activity (for sedentary leisure time). The SD was 14 MET-h/day for physical activity and 1.5 h/day for sedentary leisure time. The regression dilution ratio was 0.52 for physical activity and 0.34 for sedentary leisure time. Significance: * $p<0.05$; ** $p<0.01$; *** $p<0.001$ (FDR-adjusted p-values using the Benjamini-Hochberg method).

American Heart Association
Circulation: Genomic and Precision Medicine

Figure 2. Associations of usual total physical activity and sedentary leisure time with mean particle diameter, cholesterol and triglycerides and of these metabolic markers with risks of occlusive CVD. Conventions as in Figure 1.

Figure 3. Associations of usual total physical activity and sedentary leisure time with other metabolic traits and of these metabolic markers with risks of occlusive CVD. Conventions as in Figure 1.

Figure 4. Global comparison of 1-SD differences of 225 metabolic markers associated with 1-SD higher usual (a) physical activity and (b) sedentary leisure time *versus* logORs for occlusive CVD associated with 1-SD higher log-transformed metabolic markers. Estimates on the x-axis are the coefficients of linear regression of log-transformed metabolic markers on (a) total physical activity and (b) sedentary leisure time. Estimates on the y-axis are the coefficients of logistic regression of occlusive CVD risk (MI and IS) on log-transformed metabolic markers. r denotes Pearson's correlation coefficient.

American Heart Association.
Circulation: Genomic and Precision Medicine

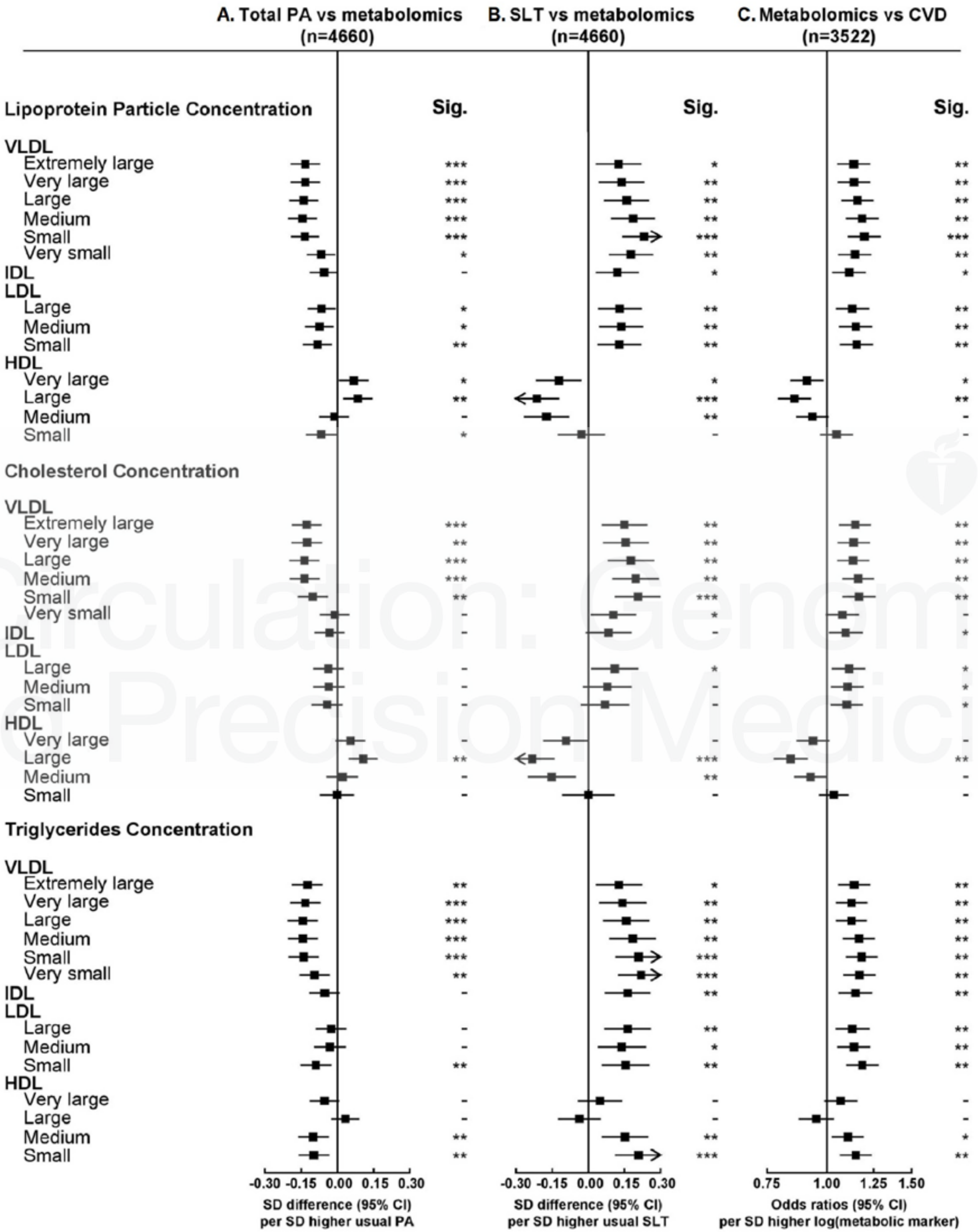

Circulation: Genomic and Precision Medicine
American Heart Association

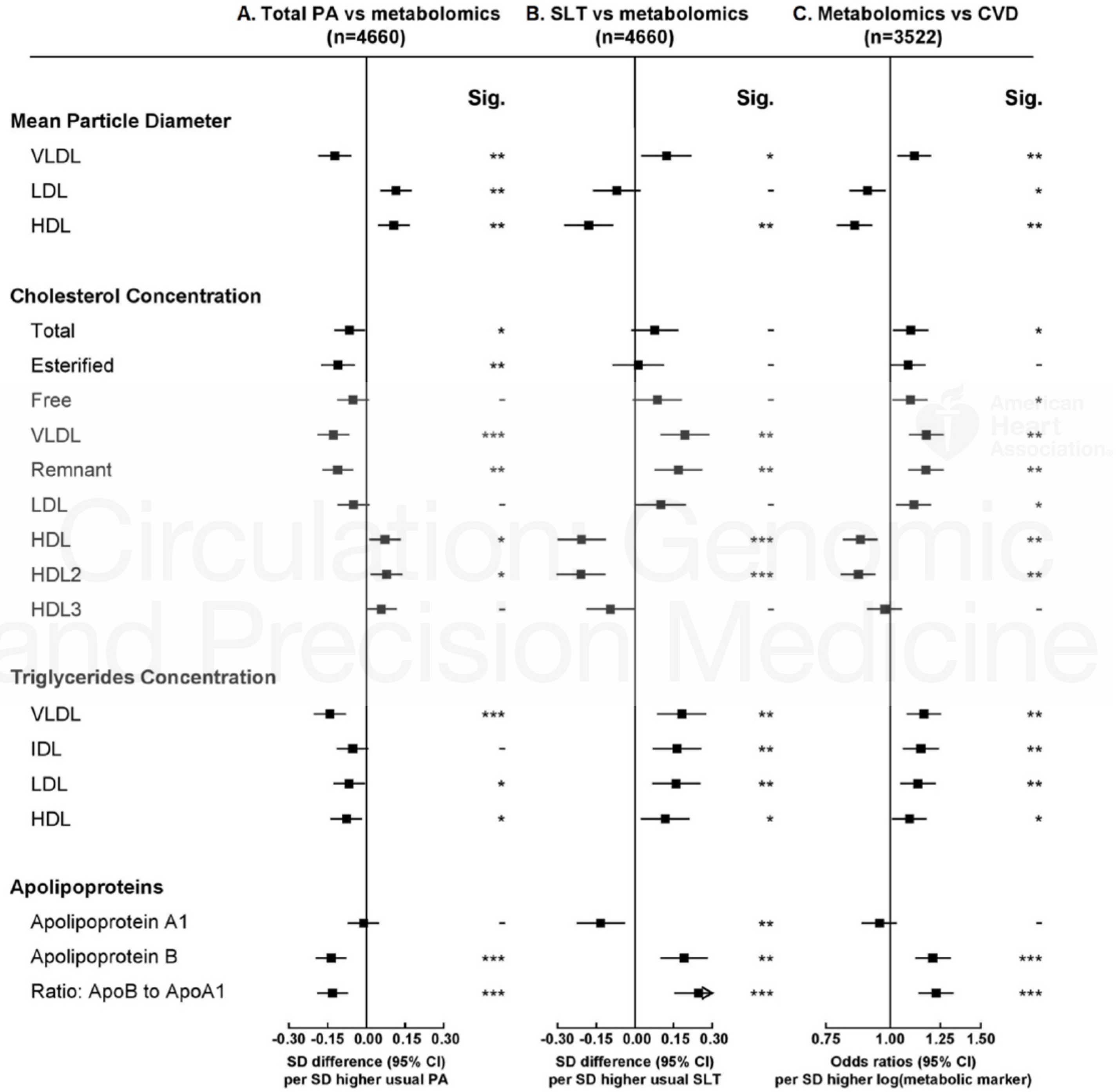

Circulation: Genomic and Precision Medicine
American Heart Association

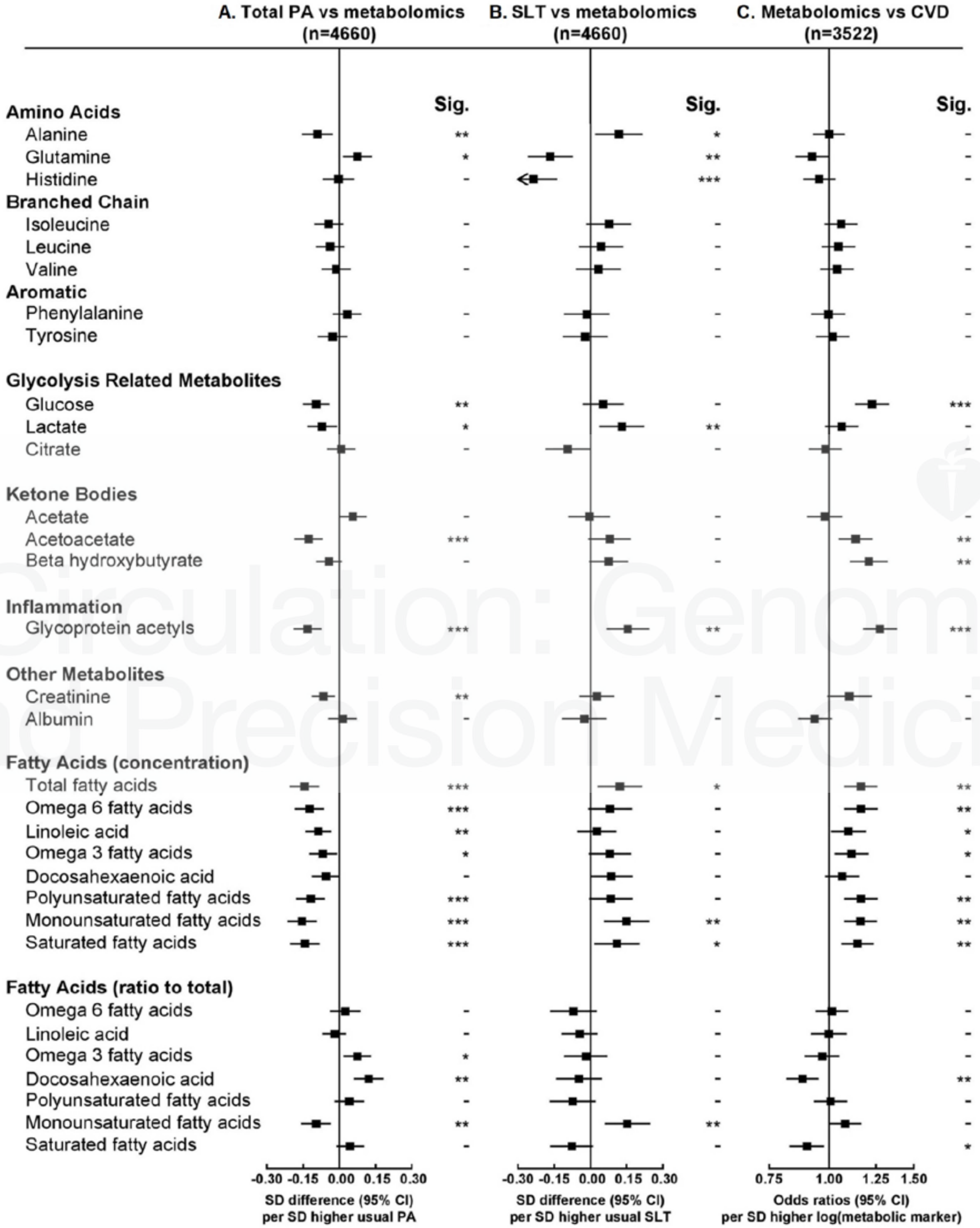

Circulation: Genomic and Precision Medicine
American Heart Association

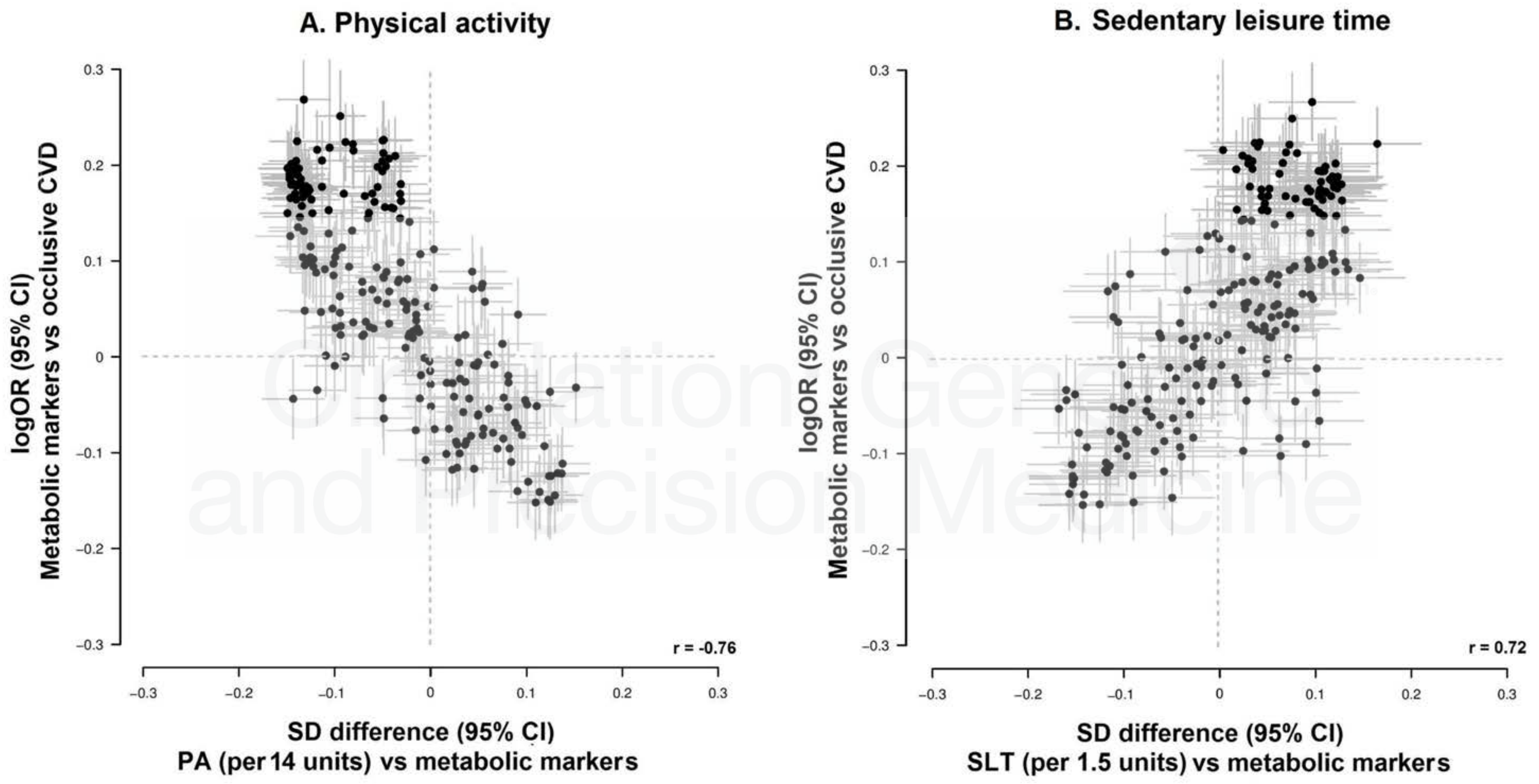
A. Physical activity
B. Sedentary leisure time
logOR (95% CI)
Metabolic markers vs occlusive CVD
r = -0.76
r = 0.72
SD difference (95% CI)
PA (per 14 units) vs metabolic markers
SLT (per 1.5 units) vs metabolic markers
0.3
0.2
0.1
0
-0.1
-0.2
-0.3
