# Supplementary material for: Physical Activity, Sedentary Leisure Time, Circulating Metabolic Markers, and Risk of Major Vascular Diseases
Source: Circ Genom Precis Med. 2019 Sep 17;12(9):e002527. doi: 10.1161/CIRCGEN.118.002527 (PMC6752700; doi:10.1161/CIRCGEN.118.002527)
Supplement: Supplementary file 3 [file hcg-12-e002527-s003.pdf]

Figure Permission

Supplementary Figure 30 is adapted from the paper referenced below. This paper is an open access article distributed under the terms of the CC-BY license, which permits unrestricted use, distribution, and reproduction in any medium.

Reference:

Bennett D, Du H, Clarke R, Guo Y, Yang L, Bian Z, Chen Y, Millwood I, Yu C, He P, et al. Association physical activity and risk of major cardiovascular diseases in Chinese men and women. *JAMA Cardiol*. 2017;2:1349-1358.
